# Oxidoreduction potential controlling for increasing the fermentability of enzymatically hydrolyzed steam-exploded corn stover for butanol production

**DOI:** 10.1186/s12934-022-01824-2

**Published:** 2022-06-27

**Authors:** Menglei Xia, Di Wang, Yiming Xia, Haijiao Shi, Zhongyu Tian, Yu Zheng, Min Wang

**Affiliations:** State Key Laboratory of Food Nutrition and Safety. Key Laboratory of Industrial Fermentation Microbiology, Ministry of Education. College of Biotechnology, Tianjin University of Science & Technology, Tianjin, 300457 People’s Republic of China

**Keywords:** *Clostridium acetobutylicum*, Steam-explosion, Fermentability, Oxidoreduction potential controlling, Mechanism analysis

## Abstract

**Background:**

Lignocellulosic biomass is recognized as an effective potential substrate for biobutanol production. Though many pretreatment and detoxification methods have been set up, the fermentability of detoxicated lignocellulosic substrate is still far lower than that of starchy feedstocks. On the other hand, the number of recent efforts on rational metabolic engineering approaches to increase butanol production in *Clostridium* strains is also quite limited, demonstrating the physiological complexity of solventogenic clostridia. In fact, the strain performance is greatly impacted by process control. developing efficient process control strategies could be a feasible solution to this problem.

**Results:**

In this study, oxidoreduction potential (ORP) controlling was applied to increase the fermentability of enzymatically hydrolyzed steam-exploded corn stover (SECS) for butanol production. When ORP of detoxicated SECS was controlled at − 350 mV, the period of fermentation was shortened by 6 h with an increase of 27.5% in the total solvent (to 18.1 g/L) and 34.2% in butanol (to 10.2 g/L) respectively. Silico modeling revealed that the fluxes of NADPH, NADH and ATP strongly differed between the different scenarios. Quantitative analysis showed that intracellular concentrations of ATP, NADPH/NADP^+^, and NADH/NAD^+^ were increased by 25.1%, 81.8%, and 62.5%. ORP controlling also resulted in a 2.1-fold increase in butyraldehyde dehydrogenase, a 1.2-fold increase in butanol dehydrogenase and 29% increase in the cell integrity.

**Conclusion:**

ORP control strategy effectively changed the intracellular metabolic spectrum and significantly improved *Clostridium* cell growth and butanol production. The working mechanism can be summarized into three aspects: First, Glycolysis and TCA circulation pathways were strengthened through key nodes such as pyruvate carboxylase [EC: 6.4.1.1], which provided sufficient NADH and NADPH for the cell. Second, sufficient ATP was provided to avoid “acid crash”. Third, the key enzymes activities regulating butanol biosynthesis and cell membrane integrity were improved.

**Supplementary Information:**

The online version contains supplementary material available at 10.1186/s12934-022-01824-2.

## Introduction

Biobutanol may play a pivotal role in the overall success of the biofuels industry and is considered as a promising next-generation liquid fuel because of its superior characteristics over ethanol [1]. However, the high cost of conventional substrates (such as maize and molasses) forms one of the main bottlenecks for economic viability. Therefore, lignocellulosic biomass is recognized as an effective potential substrate for biobutanol production because of its abundance, renewability, and cost-effective characteristics [2].

Plant biomass has evolved complex structural and chemical mechanisms for resisting the assault on its structural sugars from the microbial and animals [3]. Hence, pretreatment and hydrolysis of lignocellulosic biomass before fermentation are essential to convert the complex structure of cellulose and hemicelluloses into simple sugars [4]. During pretreatment, some fractions of cellulose and hemicellulose are converted into fermentable sugars, which can further be converted to acetone-butanol-ethanol (ABE). However, sugar and lignin degradation compounds including weak acids, furan derivatives, and phenolic compounds are also formed, which have severely inhibitory effects on the *Clostridium* [5]*.* They damage the cell membrane to maintain internal pH and make it permeable to adenosine diphosphate and some ions, inhibit glucose uptake, and, subsequently, cause cell lysis [5, 6]. Over the past years, several detoxification protocols have been introduced, including physical (e.g., adsorption with activated carbon or ion-exchange resins), chemical (e.g., lime or alkali treatment, ionic liquids, mixtures of cationic and anionic salts that melt mostly below 100 °C) or biological (e.g., laccase or peroxidase) measures [5]. To date, most of the detoxification protocols are far from satisfactory [1, 5]. And it is not feasible to remove all the inhibitors at the expense of high investment. It is of importance to explore new strategies for solving this urgent problem.

Redox potential, known as oxidation–reduction or oxidoreduction potential (ORP), reflects the overall electron transfer and redox balance involved in intracellular metabolism. Many biological functions of cells are affected by ORP levels through gene expression and enzyme synthesis, which consequently affect signal sensing and transduction, and ultimately metabolic profiles, particularly under stress conditions associated with industrial production [7]. Since extracellular ORP can be detected conveniently by the ORP electrode, it has been successfully applied to altering intracellular ORP conditions and cell metabolism [7, 8]. Vasconcelos et al. [9] proved that changing the overall degree of reduction of the substrate, using mixtures of glucose and glycerol, generated significance on the enzymatic pattern of *C. acetobutylicum*. Wang et al. [8] reported that the biphasic metabolism of *C. acetobutylicum* could be changed by ORP regulation. When using air to control the ORP of the fermentation broth at − 290 mV, earlier initiation of solventogenesis was achieved. Li et al. [10] indicated that an increase in butanol/acetone ratio and NADH regeneration could be realized when enriching the reductive environment by using the cassava-based substrate. However, to date, it remains unclear whether ORP controlling can increase the fermentability of enzymatically hydrolyzed steam-exploded corn stover (SECS) for butanol production.

The genome-scale metabolic (GSM) model analysis is a powerful tool for understanding the metabolic capacities of an organism and developing metabolic engineering strategies for strain development. By integrating all of the experimentally determined metabolic reactions taking place in an organism of interest, it can generate accurate predictions and informative hypotheses for cellular metabolism [11, 12]. It has been widely used in molecular mechanism study [13], exogenous pathway designed [14] and so on. Compared with the traditional omics methodology, it lets the researchers exactly calculate the generation and distribution of energy and electron-mediating organic cofactors NAD(P)H [15].

In this study, the effect of inhibitors on butanol biosynthesis was firstly investigated by comparing fermentation performance of detoxicated SECS and synthesized medium. Secondly, combined with the efficient detoxification method built previously [1], the effects of controlling the ORP on the fermentability of detoxicated SECS at different levels were studied. Lastly, the enhancement mechanism of ORP controlling was investigated using genome-scale metabolic flux analysis, energy status detection, enzymes activity measurement and cell membrane integrity evaluation.

## Methods

### Steam explosion pretreatment

Corn stover was obtained from a local farm in Tianjin, China. Steam explosion pretreatment was carried out in a 7.5-L batch reactor as described in our previous work [16]. About 200 g air-dried chipped corn stover was soaked in 200 mL distilled water for 15 min, fed into the reactor at 1.1 MPa (with a temperature at 183.2 °C) for 4 min. After the steam explosion, the material was washed with 1 L of 80 °C water and filtered by nylon cloth (200 meshes) and then dried at 65 °C until constant weight (< 3% of moisture content) for enzymatic hydrolysis.

### Enzymatic hydrolysis and detoxification pretreatment

Enzymatic hydrolysis experiments were conducted with the method of Wang and Chen [1]. Solid substrate concentration was set at 10% (w/v) in 50 mm acetic acid buffer with an enzyme loading of 0.2 mL/g solid substrates. The enzyme preparation, containing cellulase activities of 110 FPU/mL and xylanase activities of 1200 IU/mL, was provided by Sunson Ltd., China. The initial pH of the mixture was adjusted to 4.8 ± 0.2 using 1 M H_2_SO_4_. The hydrolysis reaction was conducted at 50 °C, with shaking at 150 rpm for 48 h. The hydrolysate was separated from the mixture by vacuum filtration, and then concentrated in a rotary vacuum evaporator at 60 °C. The total sugar in the hydrolysate was about 60 g/L. The concentrated hydrolysate of SECS was treated with 7.5% (w/v) of granular activated charcoal (No. GH-6, Beijing Guanghua Wood Factory, Beijing, China) at 30 °C with shaking at 150 rpm for 12 h and no pH adjustment. The activated charcoals were separated from SECS hydrolysate by vacuum filtration. Prior to fermentation, the concentrated hydrolysate was adjusted to pH 6.5 ± 0.5 with Ca(OH)_2_. The precipitate formed was removed by centrifugation at 8000×*g* for 15 min.

### Microorganism and culture conditions

The working strain *C. acetobutylicum* ATCC 824 was purchased from China General Microbiological Culture Collection Center and repetitively domesticated using the method of Yu et al. [17]. Stock cultures were stored at − 80 °C as 15% (v/v) glycerol stocks of cells, which were grown to an OD_600_ of 0.8–1.0. After being removed from the freezer, the strain was heat-shocked at 70 °C for 2 min, and then inoculated into a glass tube (diameter, 3 cm; 15 cm height) containing 25 mL of 7% (w/v) corn meal used as the seed medium, followed by an incubation period of 30 h at 37 °C. 10 mL of the resulting seeding suspension was inoculated into 250-mL serum bottle with 100 mL fermentation medium. After an incubation period of 12 h at 37 °C, the secondary seed broth was inoculated a 2 L flask with a working volume of 1 L fermentation medium. There are two kinds of fermentation medium used in our study: enzymatically hydrolyzed steam-exploded corn stover (SECS) medium and synthesized medium (SM). Most of the components are the same except the sugars. The sugars in SECS are from concentrated hydrolysate with a concentration of 60 g/L. The components of synthetic medium include 30 g/L pure glucose, 20 g/L xylose, 10 g/L cellobiose. The same components are: 6 g/L (NH_4_)_2_SO_4_, 1.768 g/L KH_2_PO_4_, 2.938 g/L K_2_HPO_4_, 2 g/L CaCO_3_, 10 mg/L *p*-aminobenzoic acid, and 10 mg/L biotin. In all experiments, the initial pH of the medium was adjusted to 6.5 with 1 M NaOH and heat sterilized at 115 °C for 30 min. Glucose was autoclaved separately and mixed in an anaerobic chamber. *p*-aminobenzoic acid was also added separately as a filter sterilized solution. Methyl viologen and rutin stock were sterilized by filtration and added into the all the mediums to a final concentration of 200 μM and 490 μM respectively*.* Trace flavonoids from *Rutin *[18] and Methyl viologen can help *Clostridium* cell growth and butanol biosynthesis [19]*.* All chemicals used in this study were purchased from Beijing Chemicals Factory, Beijing, China. All the experiments were carried out in an anaerobic incubator (YQX-II, Xinmiao, China), which was purged with 99.9% N_2_ to ensure 100% anaerobic conditions during the whole process.

### Analytical procedures

Acetone-butanol-ethanol (ABE) and acids (acetate and butyrate) were measured with the methods in our previous study [16]. Glucose, xylose, cellobiose, furfural, and 5-hydroxymethylfurfural were determined by high-performance liquid chromatography (Agilent 1200 HPLC, Agilent Technologies, USA) with an Aminex HPX-87H column (300 mm × 7.8 mm, Bio-Rad Laboratories Inc.) and a refractive index detector. Soluble lignin was detected by ultraviolet spectra and estimated by the method of Mussatto SI and Roberto IC [20]. All the standards were purchased from Sigma-Aldrich (St. Louis, MO). To determine the dry cell weight, 10 mL culture sample was taken from the fermentation vessel and centrifuged at 2500×*g* for 3 min. Then, cells were washed with ice-cold phosphate-buffered saline (PBS) five times and kept at 60 °C until the constant weight. The H_2_ and CO_2_ concentrations in the exhaust gas were determined using the MultiRAE IR gas monitor PGM 54 (RAE system Inc., San Jose, USA).[21].

### ORP detection and control strategy

**Fig. 1:**
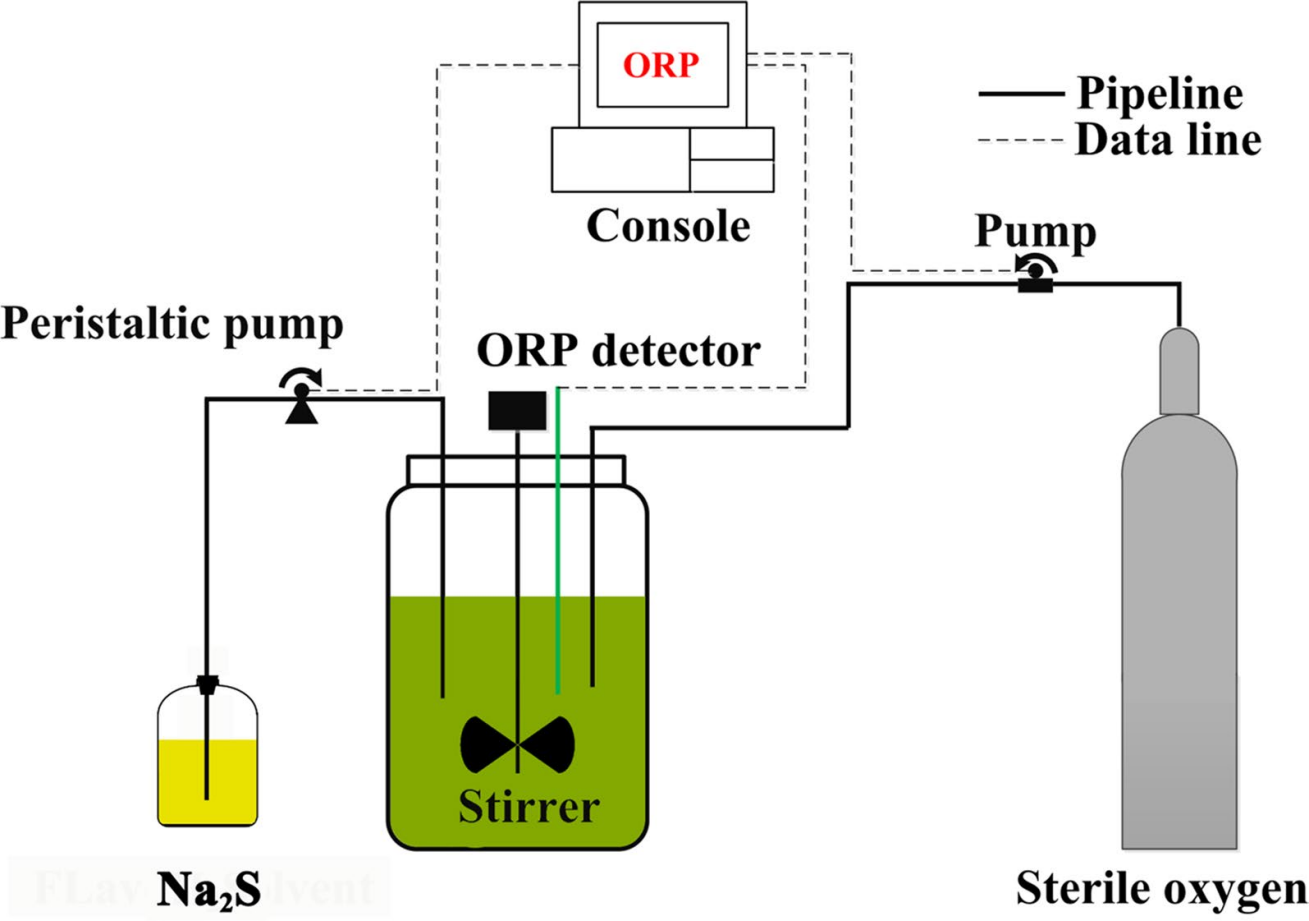
Schematic of the ORP detection and control strategy using sterile oxygen and Na2S

The ORP controlling instrument is shown in Fig. 1. An ORP electrode (Pt4805-DPAS-SC-K85; Mettler-Toledo, Switzerland) was connected with ORP console (a relay) and peristaltic pumps. ORP level of the fermentation broth was controlled at the set value by pumping the sterilized air (to increase the ORP level) or through input of 30 g/L Na_2_S (to decrease the ORP level). Before measurement, the electrode was calibrated with redox standard solution. The standard solution of ORP is 3.3 mol/L KCl solution, and the standard potential of this solution is 256 ± 2 mV at 25 °C.

### Genome scale metabolic flux analysis

The genome-scale model for *C. acetobutylicum* was adopted from the previously published model by Lee et al. [22] and the full information was given in Additional file 1. Briefly, the reconstructed metabolic network was represented in a mathematical format in a stoichiometric matrix S, where the rows correspond to the metabolites and columns correspond to the reactions in the network [23]. The specific rate for glucose uptake and the specific formation rates of lactate, acetate, butyrate, ethanol, CO_2_, H_2_, and acetone was used as the constraints. The rates were calculated as follows.

1 $$ \mu { = }\frac{{d_{Biamoss} }}{dt} $$

2 $$ r_{substrate\;or\;product} = \frac{{dc_{{substrate\;{\text{or}}\;product}} }}{dt \cdot biomass} $$

Flux balance analysis simulations were carried out using the COBRA Toolbox [14, 24]. Because the *C. acetobutylicum* strain has two distinct phases of product formation: acidogenesis and solventogenesis, the objective function was set differently. Maximizing growth was used to calculate the flux distribution in the acidogenesis phase. The analysis can be described as follows:

3 $$ \begin{gathered} Maximaze\;v_{growth} \hfill \\ s.t.\;\;\;\;\;\;S \times v = 0 \hfill \\ \qquad l \le v \le u \hfill \\ \end{gathered} $$

where *S* is the stoichiometric matrix, *l* and *u* are the lower and upper bounds on the variable vector *v* of reaction rates (fluxes).

In the solventogenesis phase, Minimization of metabolic adjustment (MOMA) method proposed by Lee et al. [22] was employed. The calculation process can be described as Eq. 4.

4 $$ \begin{gathered} {\text{Minimize:}}\;\; \hfill \\ \left\| {v^{acid} - v^{sol} } \right\|^{2} + (v_{vu\;up}^{sol} v_{ac\;out}^{acid} - 0.315v_{ac\;up}^{sol} v_{{bu\;{\text{out}}}}^{acid} )^{2} \hfill \\ {\text{Subject:}} \hfill \\ S \times v = 0,\;v_{\min } \le v \le v_{\max } \hfill \\ \end{gathered} $$

where the first term of objective function is a sum of least-squared residuals and the other term is a nonlinear constraint. V^*acid*^ is the flux vector in acidogenic phase which is calculated under the objective function as maximizing cell growth rate; V^*sol*^ is the desired flux vector in solventogenic phase; *Vsol bu up*up is the desired butyrate uptake flux in solventogenic phase; Vacid ac out ac out is the calculated acetate secretion flux in acidogenic phase; vsol ac up is the desired acetate uptake flux in solventogenic phase; vacid bu out is the calculated butyrate secretion flux in acidogenic phase; S denotes the stoichiometric matrix; v denotes the flux vector; and v_min_ and v_max_ correspond to the upper and lower bounds of v.

### Determination of enzyme activity

Preparation of cell extracts were carried out following the method of Vasconcelos et al. [9]. The samples were centrifuged at 9000×*g* at 4 °C for 30 min, and the cells were re-suspended with 3 mL 100 mM Tris hydrochloride buffer (pH 7.6) containing 2 mM DTT. The cell suspension was ultrasonically broken 4 times at 0 °C for 30 s each. The cells crushed by ultrasound were centrifuged at 13,000×*g* for 2 min to remove cell fragments. Butanol and butyraldehyde dehydrogenases (EC:1.1.1- and EC.1.2.1.10) were carried out by the method of Dürre P, Kuhn A, Gottwald M, Gottschalk GJAm and biotechnology [25] except that substrate concentrations were as following: acetaldehyde, 20 mM, butyraldehyde, 11 mM; acetyl coenzyme A (acetyl-CoA), 0.5 mM; and butyryl-CoA, 0.5 mM. Phosphotransacetylase (EC 2.3.1.8) was essayed was described by Andersch W, Bahl H, Gottschalk GJEjoam and biotechnology [26] except that crude extract was used instead of dialyzed extract. The CoA liberated was determined with DTNB [5,5′-dithiobis (2-nitrobenzoic acid)] at 410 nm (ε_410_ = 13.8 mM^−1^ cm^−1^).

### Detection of cell membrane integrity

Detection of cell membrane integrity followed the approach of Peeters [27]. 0.5 mL culture sample was taken from the fermentation vessel and centrifuged at 2500×*g* for 1 min. Then, cells were washed with 37 °C phosphate-buffered salines (PBS). After being washed two times, cells were mixed with 0.5 mL of PBS containing 2.5 mg of fluorescein diacetate (FDA) and incubated for 10 min at 37 °C in the dark. Then, 0.3 of the pretreated broth was transferred into 96-well plate. The relative levels of fluorescence were quantified in fluorospectro-photometer (F-4600, Hitachi, Japan) at an excitation wavelength of 495 nm and an emission wavelength of 535 nm respectively.

### Statistical analysis

All experiments were performed independently at least three times, and the average values with standard errors were reported. Statistical analysis was performed using the *Student’s* t-test. p values of less than 0.05 were considered statistically significant. The principal component analysis (PCA) was conducted using Python Software with *sklearn* tool kit. Before PCA, the data was firstly standardized using the function of *StandardScaler.* The PCA results were visualized by Origin software (2021b, academic version, OriginLab Corporation, Northampton, MA, USA).

## Results and discussion

### Comparison of the fermentability between detoxicated SECS and starchy feedstocks for butanol production

As Table 1 shows, the main components of SECS are glucose (59.3%), xylose (23.4%) and cellulose (11.35%); during the pretreatment process, a complex mixture of microbial inhibitors is also generated, which are mainly soluble lignin (3.55 g/L), vanillin and ferulic acid together with other aromatic compounds (see Additional file 2).

**Table 1:** Components of enzymatically hydrolyzed steam-exploded corn stover

Materials	Concentration (g/L)
Total sugar	63.75 ± 4.33
Glucose	37.83 ± 2.52
Xylose	14.92 ± 1.41
Cellobiose	7.24 ± 0.23
Acetic Acid	0.16 ± 0.03
Furfural	0.046 ± 0.011
5-hydroxymethyl furfural	0.011 ± 0.0002
Soluble lignin	3.55 ± 0.33
Other components^a^	< 0. 1 ± 0.004

^a^See Additional file 2

**Fig. 2:**
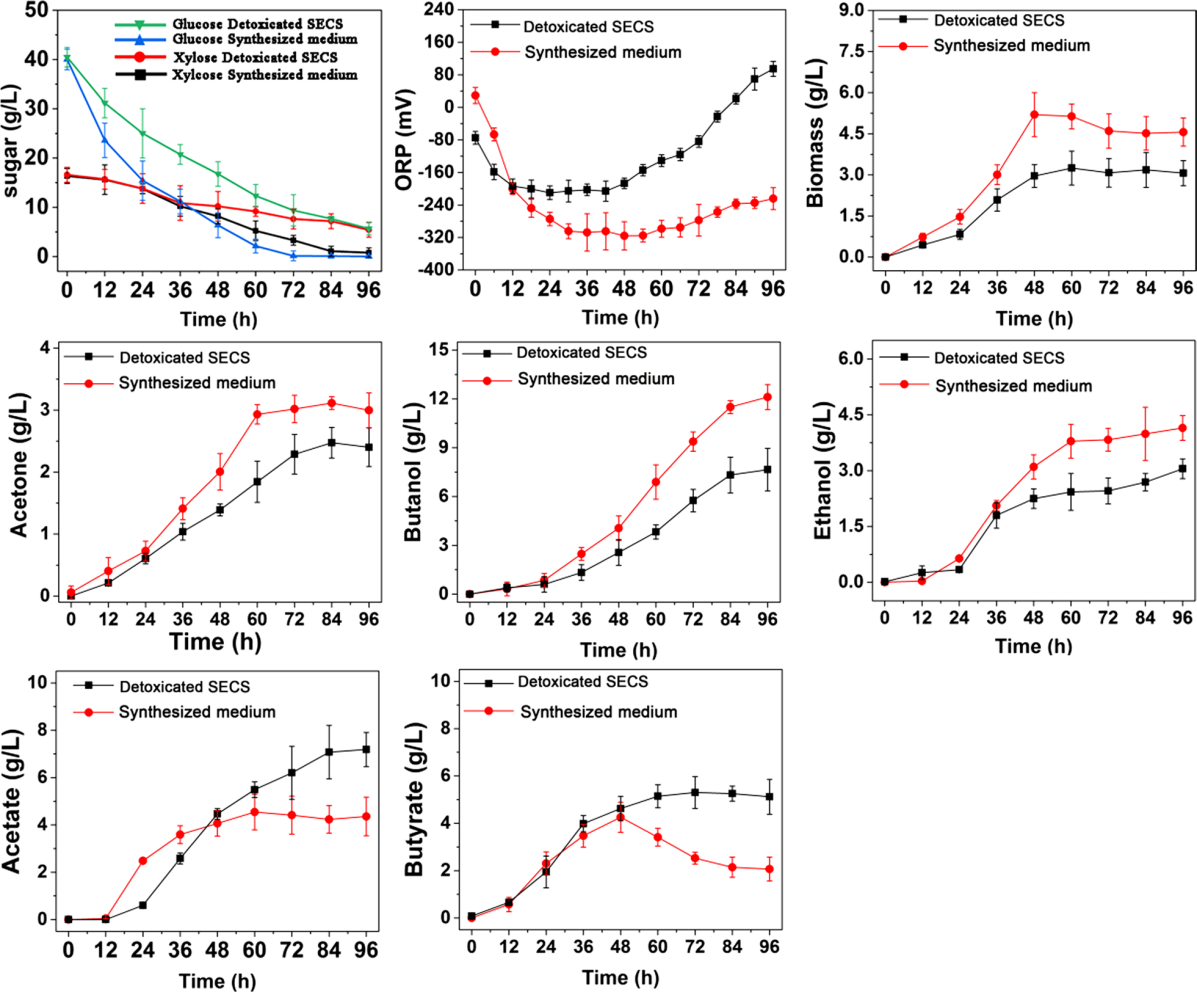
Fermentation profiles of *C. acetobutylicum* ATCC 824 with synthesized medium and detoxificated SECS

Figure 2 compares the fermentation performances of *C. acetobutylicum* ATCC 824 in SECS and synthesized medium (as reference). The order of sugar preference by *C. acetobutylicum* ATCC 824 can be summarized as glucose > xylose > cellulose. Due to inhibitors, strains showed low sugar utilization ability when in the SECS medium. The utilization rates of glucose, xylose and cellulose were 87.5%, 31.0%, and 33.3% of those in synthesized medium respectively. As a result, cell growth was inhibited, which was only 65.7% of that in synthesized medium. Wang and Chen [1] proved soluble lignin as the main fermentation inhibitor for *C. acetobutylicum* strains by supplementing inhibitors into the synthesized medium. It is because soluble lignin is phenolic compound, which causes increase in membrane fluidity, a property known to affect membrane permeability [28] causing leakage of cellular contents or even cell death [29]. There also shows significant difference in product profiles between the two groups: cells in SECS produced less alcohols (acetone, butanol and ethanol) but higher level of acids (butyrate and acetate), suggesting cell in SECS shifts metabolism to favor the biosynthesis of acids, such similar pattern was also found in our previous study [30]. It is known that biosynthesis of acids will generate more ATP than butanol for *Clostridium* cells [30–33]. A typical biphasic fermentation can be observed in synthesized medium: acetate and butyrate were accumulated mainly in the first 48 h; butyrate was then reused and solvents were produced rapidly with final production of butanol, acetone, and ethanol of 12.1 g/L, 2.9 g/L and 4.1 g/L, respectively. Different from described above, instead of absorbed by cells for butanol biosynthesis [34], butyrate in SECS kept being produced during 48-96 h, resulting in lower final alcohol productions with 7.6 g/L butanol, 2.4 g/L acetone, and 3.0 g/L ethanol. The ORP profiles also showed significant difference: the ORP of SECS decreased rapidly from the initial − 80 mV to − 170 mV within 12 h (lag phase) and then gradually rose to 157 mV until the end of the fermentation. As a contrast, the ORP in the synthesized medium decreased to − 360 mV at 36 h and kept relatively stable during the fermentation process (36–72 h), and then increased to − 220 mV during the following phase. It thus can be concluded that the fermentability of detoxificated SECS is still far lower than that of synthetic medium. The tiny inhibitors remained can still inhibit the metabolism behaviors of *C. acetobutylicum* ATCC 824 during the butanol fermentation.

### Enhancement of the fermentability of detoxificated SECS by ORP controlling

**Fig. 3:**
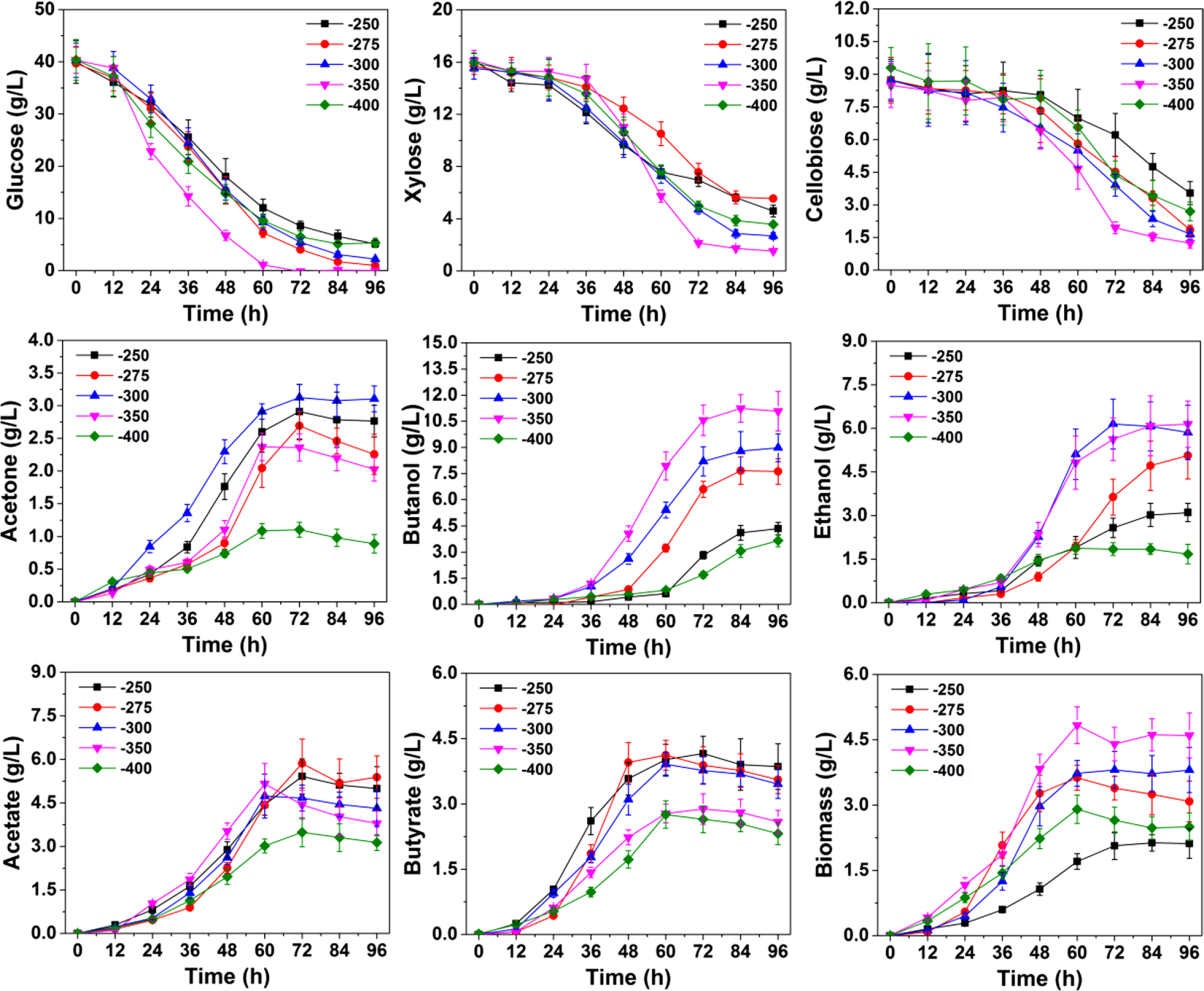
Fermentation profiles of *C. acetobutylicum* ATCC 824 under different ORP levels

To enhance the fermentability of detoxificated SECS, ORP controlling strategy was designed and carried out. The fermentation performances under different controlling strategies were summarized in Table 2 and Fig. 3 (The fermentation profiles under ORP controlling which started at 0 h were given in Additional file 3).

**Table 2:** Comparison of cell growth and the yield of acetone, butanol and ethanol using different ORP controlling strategies

ORP value	Starting time for controlling	Fermentation time (h)	End product formed (g/L)					Final Biomass (g/L)	Sugar consumed (%)		
			Ethanol	Acetate	Butanol	Butyrate	Acetone		glucose	xylose	cellobiose
Uncontrolled		96 h	4.2	7.2	7.6	4.1	2.4	3.1	94.3	80.2	81.5
− 250	0 h	96 h	1.6	2.7	3.2	2.9	0.9	1.7	40.3	30.6	34.1
− 300	0 h	96 h	2.4	3.7	4.1	2.7	1.4	2.2	74.9	50.3	50.4
− 350	0 h	96 h	2.2	4.1	3.2	3.1	2.6	2.5	84.1	64.3	55.8
− 400	0 h	96 h	0.9	1.9	2.6	1.7	1.1	1.8	31.4	26.4	22.1
− 250	24 h	96 h	3.1	5.0	4.4	4.9	2.8	2.8	87.4	80.7	79.7
− 275	24 h	90 h	5.1	5.4	7.6	3.7	2.6	3.4	96.4	84.4	80.6
− 300	24 h	90 h	5.6	4.2	8.8	3.5	3.1	3.8	97.2	84.2	82.1
− 350	24 h	90 h	5.9	3.9	10.2	2.7	2.0	4.6	98.7	86.5	88.1
− 400	24 h	96 h	1.7	3.1	3.7	2.3	0.4	2.9	86.4	76.4	70.4

When the ORP controlling was applied from the beginning (0 h) with different values (− 250, − 300, − 350, − 400 mV) respectively, cell growth was even worse than the ORP uncontrolled fermentation, indicating the optimal ORP in lag phase (0–24 h) was not a stable value. (Unless specially indicated, the discussion below refers to the ORP controlling which started at 24 h). Within the range of – 270 to − 350 mV, cell growth and sugar utilization increased with the decrease of ORP. At ORP of − 270 mV, − 300 mV, and − 350 mV, the biomass was increased by 9.6%, 22.5%, and 38.7% compared with the uncontrolled group. When ORP is − 270mv, − 300 mV, and − 350 mV, the utilization rates of glucose, xylose, and cellobiose were also increased (shown in Table 2). However, cell growth and sugar utilization were significantly reduced when the ORP was controlled at a more reductive level (less than − 400 mV), suggesting too reductive stress causes toxic effects. Such side effects of reductive stress can also be found in the studies of Wang et al. [8] and Du et al. [35]. It is reasonable because ORP is a function of pH, dissolved oxygen, equilibrium constant, and reducibility of a number of compounds dissolved in the medium [36]. Unsuitable ORP regulation would provide a more complicated and adverse effect on the metabolism of *C. acetobutylicum*, even cell death [8, 35]. When ORP was controlled at − 350 mV, butanol reached the maximal production of 10.2 g/L, 34.2% higher than that of the ORP-uncontrolled. Meanwhile, the period of fermentation was shortened from 96 to 90 h. Ethanol production also increased significantly within the ORP range of – 300 to − 350, from 4.2 g/L for uncontrolled to 5.6 g/L at − 300 mV and 5.9 g/L at − 350 mV. Compared to the uncontrolled, the maximal ethanol production was increased by 40.5%. The effect of ORP controlling on butyrate was diametrically opposite to that on butanol and ethanol. ORP at − 250 mV presented a slight promotion effect by 19.5% whereas butyrate was inhibited by 11.9%, 16.7%, 35.7% and 45.2% at − 275 mV, − 300 mV, − 350 mV and − 400 mV respectively. ORP controlling also showed inhibition effect on acetate production. The final acetate production decreased from 7.2 g/L (without ORP controlling) to 4.2 g/L at − 300 mV, 3.9 g/L at − 350 mV and 3.1 g/L at − 400 mV respectively. The increased solvents/acids ratio under ORP controlling within − 250 mV to − 350 mV indicated that the product profiles were shifted to favor solvents over acids production [35]. ORP controlling also inhibits the hydrogen production from 4.5 L of uncontrolled to 3.1 L at − 300 mV and 1.7 L at − 350 mV (the production profiles of hydrogen and carbon dioxide were given in Additional file 4). According to the previous studies conducted by [35], the decreased hydrogen production help to produce additional NADH that favors butanol biosynthesis. Strikingly, the ORP control did not affect the carbon dioxide production with a final production about 5.6 L, indicating that the carbon dioxide production is relatively rigid and not easily disturbed by ORP.

### Effect of redox regulation on intracellular metabolic distribution

**Fig. 4:**
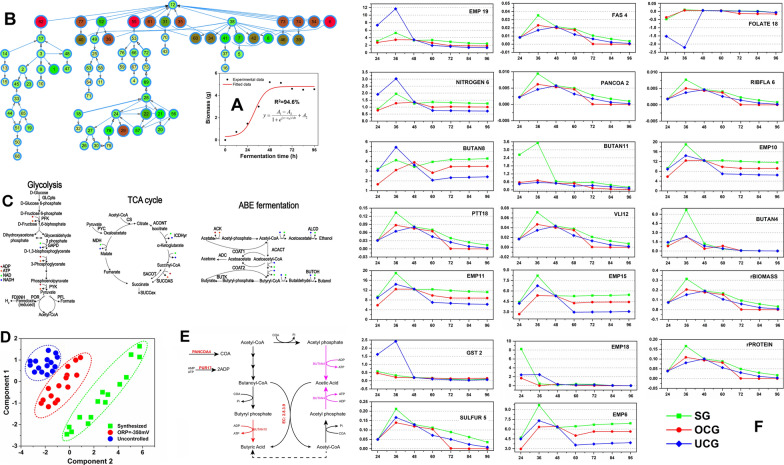
Metabolic flux distribution of *C. acetobutylicum* ATCC 824 in different culture conditions. **A** The fitted result of biomass of SG with *Boltzmann* model for the specific growth rate calculation; **B** The topology structure of the metabolic networks with the nodes with significant differences calculated (p < 0.05) among the three groups at 36 h; **C** The major redox reactions in acetone-butanol-ethanol fermentation of the bacterium *Clostridium*; **D** Principal component analysis (PCA) among the three groups with regard to metabolic flux distribution; **E** The metabolic circuit resulting in acid accumulation; **F** The flux distributions among reactions of intracellular force reduction and energy metabolism under the three conditions

Obviously, the broth ORP enhances fermentability of the detoxicated SECS by affecting the cells' metabolism. To better understand the working mechanism, genome-scale metabolic flux analysis (MFA) was performed to compare the flux profiles of *C. acetobutylicum* cells under three different culture conditions: the synthesized medium group (SG), detoxicated SECS medium with ORP control at − 350 mV (OCG) and without ORP control (UCG). The analysis result was given in Fig. 4.

Figure 4 compared the difference in flux distributions at the typical time point of 36 h (cell growth in acidogenesis phase) and 60 h (solventogenesis phase) among the three groups. Figure 4A showed the fitted result of biomass of SG with *Boltzmann* model for the specific growth rate calculation. The other two groups and the detailed calculation process together with the calculation code were given in Additional file 5. The genome-scale metabolic model used in our study consists of 432 genes, 502 reactions and 479 metabolites. Calculation was carried out using FBA constrained by experimental data [30, 37]. To assess the diversity among the three groups with regard to metabolic flux distribution, principal component analysis (PCA) was carried out (Fig. 4D). We can see that samples from the three groups can be clearly separated, indicating that the metabolic characteristic differences of *C. acetobutylicum* ATCC 824 under different fermentation.

Figure 4B showed the topology structure of the metabolic networks, which only preserves the nodes with significant differences calculated (p < 0.05) among the three groups at 36 h. The detained information of each metabolite and reaction in Fig. 4B was given in Additional file 6. The metabolic fluxes of the three groups at different time points were given in Additional file 7. The substrates with the most significant differences are succinyl-CoA (No.62, KEGG: C00091), pyruvate (No. 55, KEGG: C00022) and N-Acetyl-l-glutamate 5-semialdehyde (No. 6, KEGG: C01250). Pyruvate and succinyl-CoA are among the 12 basic biosynthetic precursor compounds that are used to build macromolecules such as nucleic acids and proteins [38]. Their differences may be related to cell growth. N-acetyl-l-glutamate 5-semialdehyde is one of the essential precursors for arginine synthesis (Module ID: M00028 in KEGG database). Previous studies have shown that the synthesis of arginine is an energetically expensive process. The cell has to supply high amounts of ATP for this process. Therefore, N-acetyl-l-glutamate 5-semialdehyde might be closed with the differences in the availability of ATP [30, 39]. However, it is difficult to determine the potential bottlenecks limiting the production based on such information. Hence, it seems highly important to get further insight into the underpinning metabolism. Since ORP directly affects intracellular electron transfer and redox balance involved in intracellular metabolism, the analysis on NADPH, NADH and ATP was carried out. Figure 4C summarized the major redox reactions in acetone-butanol-ethanol fermentation by the bacterium *Clostridium* [40]. Figure 4F, Tables 3, and 4 exhibited the flux distributions among reactions of intracellular force reduction and energy metabolism under the three conditions. In our model, there are 23 reactions involving NADPH, 16 reactions involved NADH and 61 reactions involving ATP. The metabolism of NADPH in the three groups is given in Table 3. At 36 h, UCG owned the most active metabolism of NADPH with a total NADPH flux as high as 11.44 mmol/g/h, which is 2.1 and 3.26-fold of SG and OCG. The contributions of each reaction in SG and OCG were similar, except the total flux. In these two groups, respiratory chain was the main source of NADPH, accounting for about 97.7%. Then folate (CA_C2083) and riboflavin synthesis (CA_C0590) account for about 3.09%. However, in the UCG group, the folate synthesis pathway consumed 19.32% of NADPH instead of generation, suggesting the cells in this group consume a large amount of folic acid. As an important cofactor, folic acid is involved in numerous intracellular reactions, including single carbon transfer reactions, the synthesis of purine, pyrimidine, amino acid [41, 42]. They are also the reactions which cells tend to intensify when under stress [4, 43, 44]. That might explain the greater consumption of folic acid in UCG. Compared with 36 h, the NADPH related fluxes were similar among the three groups at 60 h. The main source of NADPH were respiratory chain (EMP19, 94.99–95.68%) and TCA cycle (PYR3, 2.65–3.00%). Amino acid metabolism (CA_C0510) and carbohydrate metabolism (SULFUR5, CA_C2390) at 60 h tended to decrease. The reaction of fatty acids (CA_C1589, CA_C0764) and COA synthesis (CA_C3254) was significantly enhanced.

**Table 3:** Simulation of metabolic flux of the NADPH generation/consumption reaction of *C. acetobutylicum* ATCC 824 strain under the different conditions

Reaction	36 h						60 h					
	SG		OGG		UCG		SG		OGG		UCG	
	Flux	Percentage	Flux	Percentage	Flux	Percentage	Flux	Percentage	Flux	Percentage	Flux	Percentage
EMP19	5.28	97.91	3.43	97.82	11.44	99.97	9.30	94.99	7.95	95.68	5.28	95.52
AMSU8	− 0.03	− 0.63	− 0.02	− 0.55	− 0.02	− 0.14	0	0	0	0	0	0
NITROGEN6	− 1.95	− 36.19	− 1.25	− 35.83	− 3.03	− 26.51	0	0	0	0	0	0
SULFUR5	− 0.64	− 11.78	− 0.45	− 12.72	− 0.31	− 2.70	0	0	0	0	0	0
GST2	− 0.33	− 6.09	− 0.21	− 5.88	− 2.42	− 21.16	− 0.12	− 1.26	− 0.11	− 1.38	− 0.08	− 1.43
GST3	− 0.23	− 4.34	− 0.15	− 4.20	− 2.38	− 20.76	− 0.05	− 0.56	− 0.05	− 0.61	− 0.04	− 0.63
VLI3	− 0.07	− 1.35	− 0.05	− 1.40	− 0.04	− 0.31	− 0.04	− 0.39	− 0.04	− 0.43	− 0.02	− 0.44
VLI7	− 0.28	− 5.12	− 0.17	− 4.83	− 0.13	− 1.17	− 0.15	− 1.49	− 0.14	− 1.63	− 0.09	− 1.69
LYS2	− 0.09	− 1.75	− 0.06	− 1.65	− 0.05	− 0.40	− 0.05	− 0.51	− 0.05	− 0.56	− 0.03	− 0.58
PRO4	− 0.08	− 1.41	− 0.05	− 1.33	− 0.04	− 0.32	0.01	0.12	0.01	0.13	0.01	0.14
PTT4	− 0.17	− 3.18	− 0.11	− 3.11	− 0.08	− 0.73	− 0.09	− 0.93	− 0.08	− 1.01	− 0.06	− 1.05
UREA3	− 0.1	− 1.82	− 0.06	− 1.65	− 0.05	− 0.42	0	0	0	0	0	0
PYRM16	− 0.24	− 4.42	− 0.16	− 4.43	− 0.12	− 1.01	0	0	0	0	0	0
PL7	− 0.2	− 3.76	− 0.13	− 3.68	− 0.10	− 0.86	0.10	1.03	0.09	1.12	0.06	1.16
FAS3	− 0.06	− 1.1	− 0.04	− 1.07	− 0.03	− 0.25	− 0.22	− 2.29	− 0.21	− 2.50	− 0.14	− 2.59
FAS4	− 0.49	− 9.16	− 0.32	− 9.14	− 0.24	− 2.10	− 0.04	− 0.45	− 0.04	− 0.49	− 0.03	− 0.50
FAS5	− 0.08	− 1.42	− 0.05	− 1.49	− 0.04	− 0.33	− 0.02	− 0.17	− 0.02	− 0.19	− 0.01	− 0.19
FAS6	− 0.04	− 0.72	− 0.03	− 0.82	− 0.02	− 0.17	− 0.16	− 1.66	− 0.15	− 1.81	− 0.10	− 1.87
FAS7	− 0.29	− 5.33	− 0.20	− 5.70	− 0.14	− 1.22	− 0.01	− 0.12	− 0.01	− 0.13	− 0.01	− 0.14
PANCOA2	− 0.01	− 0.17	− 0.01	− 0.27	0.00	− 0.04	0.00	− 0.05	0.00	− 0.06	0.00	− 0.06
RIBFLA6	0.01	0.14	0.01	0.28	0.00	0.03	0.00	0.04	0.00	0.05	0.00	0.05
FOLATE13	− 0.01	− 0.26	− 0.01	− 0.26	− 0.01	− 0.06	0	0	0	0	0	0
FOLATE18	0.11	1.95	0.07	1.90	− 2.21	− 19.32	0.01	0.12	0.01	0.13	0.01	0.13
FOLATE12	0	0	0	0	0	0	− 0.01	− 0.08	− 0.01	− 0.08	0.00	− 0.09
NITROGEN4	0	0	0	0	0	0	− 0.01	− 0.14	− 0.01	− 0.15	− 0.01	− 0.16
PRO2	0	0	0	0	0	0	− 0.04	− 0.41	− 0.04	− 0.45	− 0.03	− 0.46
PYR3	0	0	0	0	0	0	0.26	2.65	0.24	2.90	0.17	3.00

The unit of the flux was mmol/g/h. The abbreviations of the reactions were provided in Additional file 1

*SG* the synthetized medium group, *OCG* detoxicated SECS medium with ORP control at − 350 mV, *UCG* detoxicated SECS medium without ORP control (UCG)

**Table 4:** Simulation of metabolic flux of the NADH generation/consumption reaction of *C. acetobutylicum* ATCC 824 strain under the different conditions

Reaction	36 h						60 h					
	SG		OGG		UCG		SG		OGG		UCG	
	Flux	Percentage	Flux	Percentage	Flux	Percentage	Flux	Percentage	Flux	Percentage	Flux	Percentage
EMP10	19.01	94.47	12.43	94.81	14.42	78.25	12.35	74.56	9.84	76.88	6.99	75.38
EMP18	0.41	2.03	0.45	3.4	2.48	13.44	3.39	20.48	2.63	20.53	1.94	20.88
BUTAN1	− 0.26	− 1.28	− 1.03	− 8	1.37	7.41	− 0.36	− 2.19	− 0.35	− 2.75	− 0.32	− 3.43
BUTAN2	− 0.22	− 1.09	− 1.01	− 7.81	− 0.88	− 4.76	− 0.35	− 2.10	− 0.84	− 6.53	− 0.41	− 4.38
BUTAN6	− 4.13	− 20.54	− 3.11	− 24.08	− 5.44	− 29.55	− 3.95	− 23.84	− 2.83	− 22.13	− 2.08	− 22.40
BUTAN8	− 8.26	− 41.08	− 6.21	− 48.17	− 10.89	− 59.10	− 6.80	− 41.03	− 4.70	− 36.72	− 3.57	− 38.52
BUTAN11	− 3.52	− 17.49	− 0.7	− 5.44	− 0.56	− 3.01	− 0.55	− 3.32	− 0.48	− 3.78	− 0.29	− 3.14
BUTAN12	− 3.52	− 17.49	− 0.7	− 5.44	− 0.56	− 3.01	− 0.15	− 0.88	− 0.63	− 4.96	− 0.20	− 2.11
TCA2	− 0.07	− 0.33	− 0.04	− 0.33	− 0.03	− 0.17	0.16	0.94	0.00	0.02	0.02	0.25
VLI12	0.07	0.36	0.03	0.36	0.03	0.19	0.04	0.23	0.04	0.27	0.02	0.26
HIS9	0.02	0.12	0.02	0.12	0.01	0.06	0.01	0.08	0.01	0.09	0.01	0.09
HIS10	0.02	0.12	0.02	0.12	0.01	0.06	0.00	0.00	0.00	0.00	0.00	0.00
PTT18	0.13	0.66	0.07	0.68	0.06	0.35	0.00	0.00	0.00	0.00	0.00	0.00
PUR27	0.03	0.17	0.02	0.17	0.02	0.09	0.15	0.91	0.02	0.15	0.05	0.59
PYRM4	0.05	0.26	0.02	0.26	0.03	0.14	0.03	0.17	0.03	0.20	0.02	0.19
FOLATE16	− 0.14	− 0.72	− 0.09	− 0.73	− 0.07	− 0.38	0.06	0.34	0.05	0.40	0.04	0.38
EMP16	0	0	0	0	0	0	− 9.29	− 56.09	− 7.99	− 62.41	− 5.42	− 58.43
GST9	0	0	0	0	0	0	0.38	2.28	0.18	1.41	0.18	1.95
LIMPIN3	0	0	0	0	0	0	0.00	0.03	0.00	0.03	0.00	0.03
PL6	0	0	0	0	0	0	− 0.11	− 0.65	− 0.10	− 0.78	− 0.07	− 0.74
TCA7	0	0	0	0	0	0	− 0.10	− 0.58	− 0.09	− 0.69	− 0.06	− 0.66

The unit of the flux was mmol/g/h

*SG* the synthetized medium group, *OCG* detoxicated SECS medium with ORP control at − 350 mV, *UCG* detoxicated SECS medium without ORP control (UCG)

Table 4 shows the metabolism of NADH in the three groups. At 36 h, the NADH fluxes of the three groups were 19.74, 13.06, and 18.43 mmol/g/h respectively. In SG and OCG, the contribution of EMP pathway to NADH was 94%, while that in UCG was only 78.25%. On the contrary, the respiratory chain intensity of the latter group was 6.05 times and 5.51 times that of the first two groups, respectively. It is suggested that the inhibitor decreased the metabolic intensity of EMP. As a compensation mechanism, cells enhanced the metabolic intensity of the respiratory chain. In the UCG, reaction **Butan1 (**acetaldehyde → acetyl coenzyme A) and reaction **Butan8** (acetyl coenzyme A → 3-hydroxybutyryl COA) were significantly improved. This change can decrease the synthesis of butyryl COA and ethanol, so as to save the NADH consumption. At 60H, not surprisingly, SG and OCG owned high flux in almost all the NADH-involved reactions. The total NADH flux of the three groups was 16.57, 12.80, and 9.27 mmol/g/h respectively. Because NADH is the major limiting factor for butanol synthesis [45–47]. High level of NADH in SG and OCG can drive the generation of more butanol in these two groups [48].

Additional file 8 shows the metabolism of ATP in the three groups. In the genome-scale model, there are 61 reactions involved in ATP metabolism, accounting for 12.15% of the total reactions, indicating that ATP metabolism has a very wide impact on cell metabolism. The total ATP flux of the three groups were 44.91, 35.89, and 29.11 mmol/g/h respectively. ATP is mainly used for bacterial synthesis at 36 h, accounting for 42.32% in SG, 42.72% in OCG and 40.16% in UCG of the total ATP respectively (seen Additional file 8). Compared with UCG, four reactions in OCG has significantly enhanced, which were FOLATE19 (CA_C3201/[EC:6.3.4.3]) increased by 159 times, GST4 (CA_C1235/[EC:2.7.1.39]) increased by 116 times, GST1 (CA_C0278/[EC:2.7.2.4] or CA_C1810/[EC:2.7.2.4]) increased by 11 times and TCA1 (CA_C2660/[EC:6.4.1.1]) increased by 6.24 times. FOLATE19 (CA_C3201/[EC:6.3.4.3]) is the synthesis of 10-Formyltetrahydrofolate. This substance is the precursor of many cofactors, suggesting, again, ORP induced high-speed synthesis of cofactors of *C. acetobutyricum.* GST4 and GST1 represent the reactions catalyzed by ***homoserine kinase*** [EC:2.7.1.39] and ***aspartate kinase*** [EC:2.7.2.4] respectively. They are the key enzymes in aspartate metabolic pathway, which controls the biosynthesis of lysine, methionine, threonine, and isoleucine. TCA1 is the reaction catalyzed by pyruvate carboxylase [EC:6.4.1.1]. As the key enzyme of oxaloacetate replenishment pathway in bacteria, it serves as the gate of carbon flow into TCA cycle. In other words, folate, amino acid, and TCA cycle of cells were greatly improved under the controlling of oxidoreduction potential. At 60 h the fluxes of four reactions in UCG were at a higher level. it showed 3.23 times higher of PUR17 (CA_C3112/EC: 2.7.4.3), 1.67 times higher of PANCOA4 (CA_C3200/[EC:2.7.1.33]), 1.66 times higher of BUTAN10 (CA_C3075/[EC:2.7.2.7]) and 1.53 times higher of BUTAN4 (CA_C1743/[EC:2.7.2.1]) of that in OCG. PUR17 [EC: 2.7.4.3] represents the conversion reaction of ATP to ADP, indicating that UCG has a higher ADP generation rate. PANCOA4 [EC:2.7.1.33] is a key enzyme that catalyzes COA synthesis. BUTAN4[EC:2.7.2.1] and BUTAN10[EC:2.7.2.7] are the key enzymes that catalyze butyryl phosphate to butyric acid and butanoyl-COA to butyryl phosphate, respectively. These four key enzymes formed a reaction circuit resulting in acid accumulation. The illustration of the metabolic circuit is given in Fig. 4E. The circuit includes three parts: (I) PANCOA4 and PUR17 supply COA and ADP respectively; (II) COA and ADP are catalyzed by Butan4 [EC: 2.7.2.1] and butan10 [EC: 2.7.2.7] for butyric acid and acetic acid biosynthesis; and (III) Under the catalysis of butyryl-CoA-acetoacetate CoA-transferase (EC: 2.8.3.9), acetic acid can capture the CoA group from butanoyl-CoA and convert the latter into butyric acid. The resulted acetoacetyl-CoA can further convert into butyric acid in this circuit. Wang et al. [49] and Maddox et al. [50] studied the cause of *“acid crash”* by adding acid to the culture medium. For the first time, we found a new metabolism circuit that may cause butyric acid accumulation by metabolic pathway analysis method.

### Effect of redox regulation on intracellular redox state

**Fig. 5:**
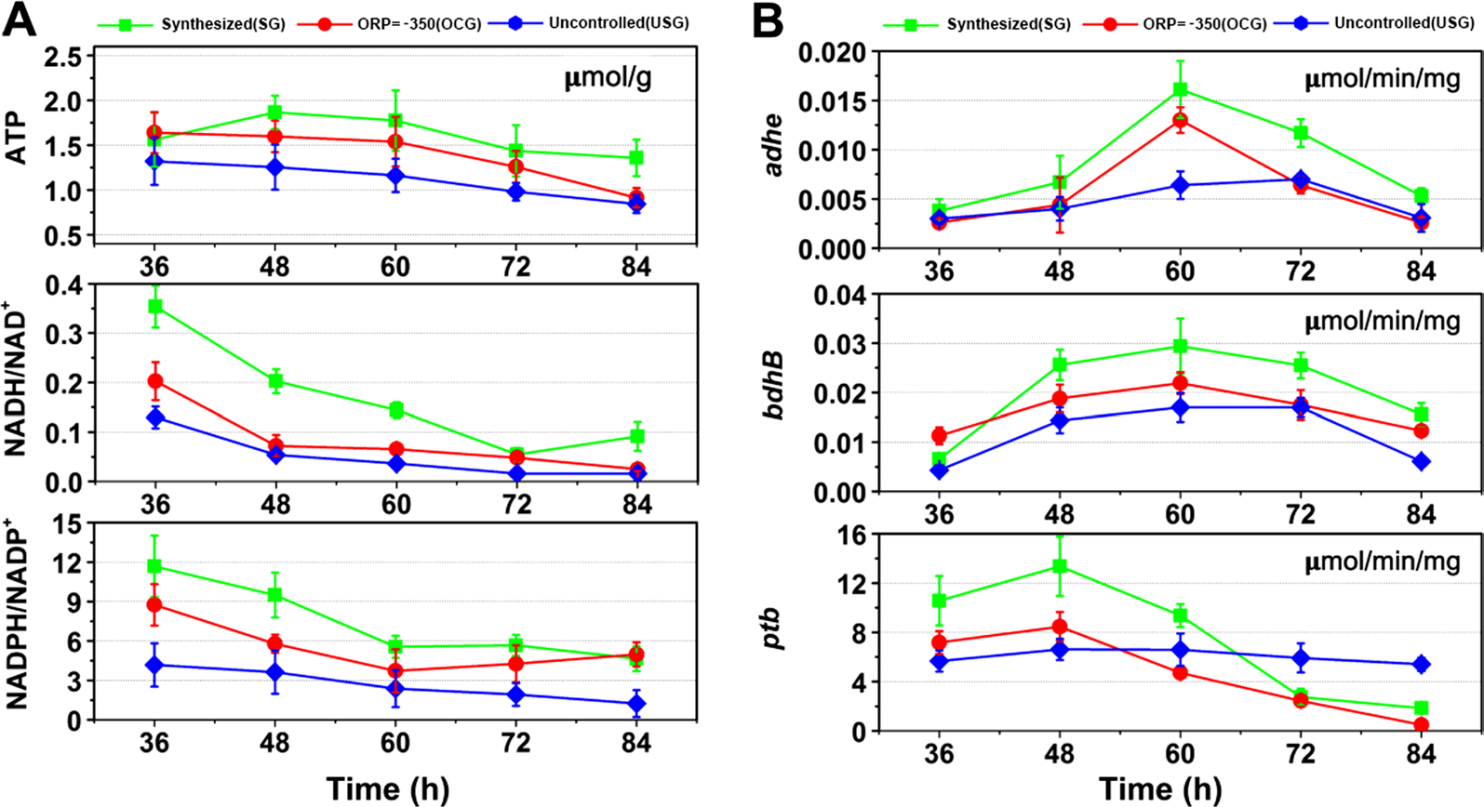
The key components analysis for the performance distinctions of *C. acetobutylicum* ATCC 824 under different culture conditions. **A** The concentrations of key metabolites responsible for intercellular energy status; **B** The activities of key enzymes responsible for butanol biosynthesis

Metabolic flux reflects the instantaneous change in cell metabolism. In order to further confirm the real state of cells, we measured the key metabolites, including ATP concentration, NADH/NAD^+^ and NADPH/NADP^+^, within *C. acetobutylicum* ATCC 824 from the three groups during the solvent-producing phase. As shown in Fig. 5a, all the factors measured in SG kept the highest level among the three groups, followed by those in OCG. Taking the time point of 60 h as an example (when the butanol biosynthesis rate was the highest at this point), the ATP concentration in SG is 1.2-fold and 1.5-fold of that in OCG and UCG, respectively; the NADH/NAD + ratio in SG is 2.2-fold of that in OCG and 4.0-fold of that in UCG; and the NADPH/NADP^+^ ratio in SG is 1.4-fold of that in OCG and 2.3-fold of that in UCG correspondingly. High energy and reduced power availability form one of the main reasons for high butanol production in SG and OCG. Meanwhile, we also detected the activities of key enzymes in the butanol biosynthesis and the result was shown in Fig. 5b. The high activities of butyraldehyde and butanol dehydrogenase in SG explained its high butanol production in fermentation. Compared with the ORP uncontrolled group, the activities of butyraldehyde and butanol dehydrogenase in OCG were increased by 2.1-fold and 1.2-fold. Meanwhile, the phosphotransbutyrylase activity was decreased by 29%, indicating that ORP controlling shifts more butyryl-CoA towards butanol biosynthesis at the expense of butyrate. It is interesting to note that phosphotransbutyrylase in UCG kept at a stable level, suggesting butyrate was produced continuously during the whole process. This result is quite consistent with our above analysis.

### ORP regulation changes cell membrane and cell wall integrity

A major drawback of solvent production by microorganisms is the toxic effect of the alcohols, especially n-butanol, on the cells themselves [51]. The accepted dogma is that toxicity in the ABE fermentation is due to chaotropic effects of butanol on the cell membranes and cell walls of the fermenting micro-organisms [34, 52, 53]. Therefore, it is necessary to verify the effects of ORP on them.

The changes of the cell wall of *C. acetobutylicum ATCC 824* under different culture conditions were observed using scanning electron microscopy (SEM). The experiment method and result were given in Additional file 9. SEM results showed that the worst cell wall damage occurred on the cells grown in UCG, followed by OCG control and SG. Obviously, cell wall integrity was improved by ORP.

**Fig. 6:**
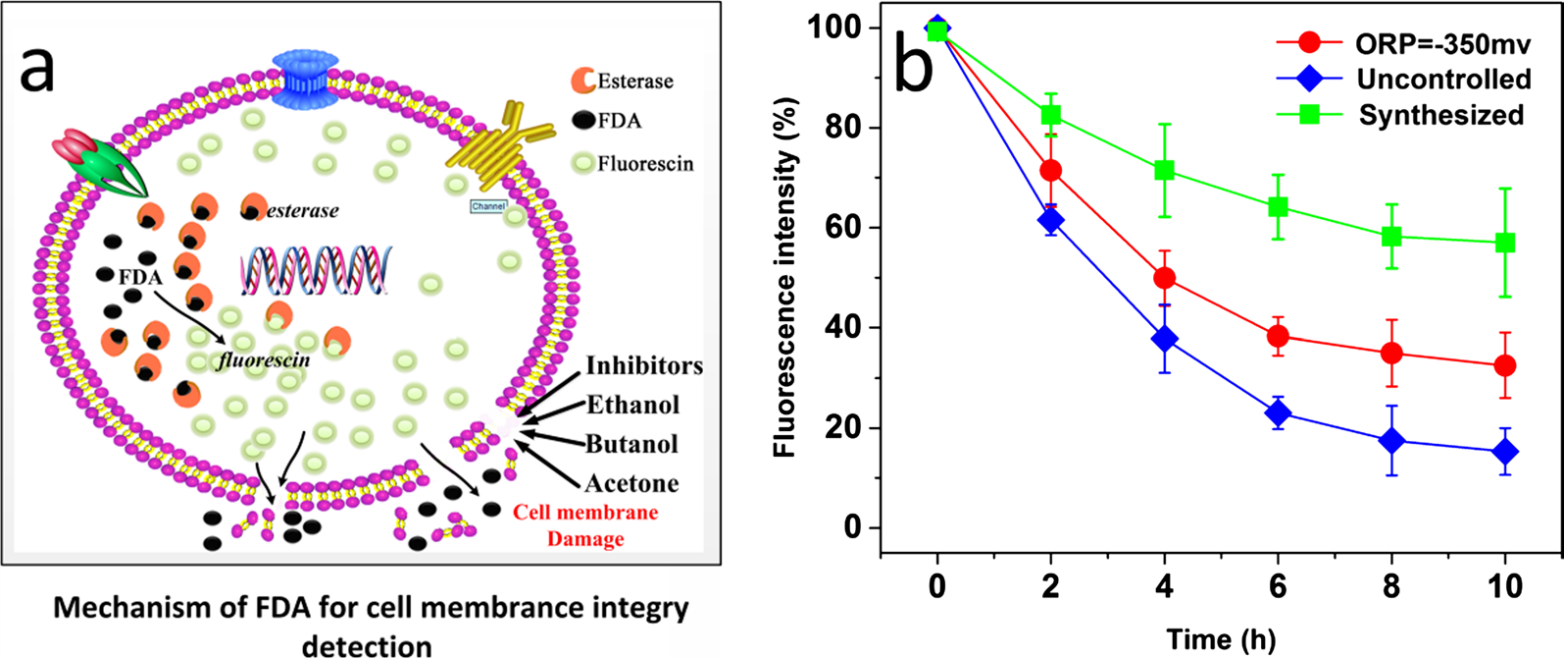
The cell membrane integrity measurement of *C. acetobutylicum* ATCC 824 under 20 g/L butanol with different culture conditions. ** A** The working mechanism of fluorescein diacetate (FDA) for detection of cell membrane integrity; ** B** The changes of cell membrane integrity of C. acetobutylicum ATCC 824 under butanol stress

Cell membrane integrity was studied using the Fluorescein diacetate (FDA) method. FDA is a cell-permeant esterase substrate. As Fig. 6a shows, when absorbed, it can be converted into green fluorescent compound “*fluorescin*” by esterase in the living cell, which can be detected by measuring the fluorescence or absorbance of the sample [54]. When the cell membrane was damaged and the permeability increases, FAD will leak from the cell, insulting the decrease of fluorescence intensity. Therefore, it can be used to judge the cell membrane integrity by the change in fluorescence intensity [55]. In the comparison experiments, butanol was supplemented into the three groups (SG, OCG, and UCG) to keep at a final concentration of 20 g/L and samples were withdrawn every 2 h for cell membrane detection. The result was given in Fig. 6b. We can see that the fluorescence intensity dropped quickly during the first 6 h, with a percentage of 35.1% in SG, 61.7% in OCG and 77.3% in UCG, suggesting there exists differences in the cell membrane integrity among the three groups. Compared with UCG, the cell membrane integrity was significantly increased in OCG. It also can be found that the decrease of fluorescence intensity trended smaller with processing time increased, especially after 6 h, indicating cells began to adapt to the butanol stress. Similar findings have been reviewed in the studies [52].

Butanol transport across the cell membrane is governed by diffusion [34, 56]. When the amount of butanol exceeds the adsorption capacity of phospholipid bilayer of cell membrane for butanol, the interaction between lipid-lipid is weakened, and the stability of phospholipid bilayer will be seriously damaged. This will result in the leakage of cellular contents and subsequently cell death. Previous researches showed that cell membrane has complex mechanism to cope with butanol stress [34]. It includes increasing the saturation of cellular lipids to decrease content leakage [57, 58], synthesizing membrane plasmalogens with protective effect [58], activating the transporter proteins to pump out butanol and maintain the intracellular pH [59].

## Conclusions

This paper applied the ORP controlling strategy to enhance the butanol production with enzymatically hydrolyzed steam-exploded corn stover (SECS). At the optimal ORP level, solvent and butanol production reached 18.1 g/L and 10.2 g/L, an 27.5% and 34.2% increase compared with the ORP uncontrolled group, respectively. In-depth analysis showed three issues that resulted in significant improvement in cell growth and butanol production: First, Glycolysis and TCA circulation pathway are strengthened through key nodes such as pyruvate carboxylase [EC: 6.4.1.1], which provides sufficient NADH and NADPH for the cell. Second, sufficient ATP is provided to avoid “*acid crash*”. Third, the key enzymes activities regulating butanol biosynthesis and cell membrane integrity were improved.

## Supplementary Information

**Additional file 1. ** Gene–Protein–Reaction realationship, metabolite abbreviation, and whole reaction set used in the model.

**Additional file 2.** Low molecular weight compounds released from lignin due to degradation.

**Additional file 3. ** The fermentation profiles of *C. acetobutylicum* ATCC 824 under different ORP levels which started at 0 h.

**Additional file 4.** The gas production profiles of *C. acetobutylicum* ATCC 824 under different ORP levels.

**Additional file 5.** Calculation process of the specific growth rates (μ), specific substrate consumption rates, and specific product secretion rates.

**Additional file 6.** Details of compounds in the model.

**Additional file 7.** The flux of each reaction at different times under different conditions.

**Additional file 8.** Simulation of metabolic flux of the ATP generation/consumption reaction of *C. acetobutylicum* ATCC 824 strain under the different conditions.

**Additional file 9.** The changes of cell wall of *C. acetobutylicum* ATCC 824 under different culture conditions.

## Data Availability

All data generated or analyzed in this study are included in the published article.
