# Supplementary material for: Oxidoreduction potential controlling for increasing the fermentability of enzymatically hydrolyzed steam-exploded corn stover for butanol production
Source: Microb Cell Fact. 2022 Jun 27;21:130. doi: 10.1186/s12934-022-01824-2 (PMC9238237; doi:10.1186/s12934-022-01824-2)
Supplement: Supplementary file 1 — Additional file 1. Gene–Protein–Reaction realationship, metabolite abbreviation, and whole reaction set used in the model. [file 12934_2022_1824_MOESM1_ESM.pdf]

Additional file 1. Gene-Protein-Reaction relationship, metabolite abbreviation, and whole reaction set used in the model

List of Genes

ORF	Gene Name	Definition	Part of Complex	E.C. Number	Reaction 1	Reaction 2	Reaction 3	Reaction 4	Reaction 5	Reaction 6
CAC0015	*serA*	D-3-phosphoglycerate dehydrogenase	No	1.1.1.95	3PG + NAD -> 3PHP + NADH					
CAC0022	*asd*	aspartate-semialdehyde dehydrogenase	No	1.2.1.11	4PASP + NADPH -> ASPSA + Pi + NADP					
CAC0025	*dcd*	deoxycytidine triphosphate deaminase	No	3.5.4.13	CTP -> UTP + NH3	dCTP -> dUTP + NH3				
CAC0027	*pyrE*	orotate phosphoribosyltransferase	No	2.4.2.10	OROT + PRPP -> OROT5P + PPi					
CAC0028	*hydA*	hydrogene dehydrogenase	No		Fd(Red) -> Fd(Ox) + H2					
CAC0031	*psdD*	phosphatidylserine decarboxylase	No	4.1.1.65	PS -> PE + CO2					
CAC0089	*serA*	D-3-phosphoglycerate dehydrogenase	No	1.1.1.95	3PG + NAD -> 3PHP + NADH					
CAC0091	*ilvC*	ketol-acid reductoisomerase	No	1.1.1.86	2AHBUT <-> 3H3MOP	3H3MOP + NADPH <-> 23DHMP + NADP	ACLAC <-> 3H3MOB	3H3MOB + NADPH <-> 23DHMB + NADP		
CAC0094		ferredoxin-nitrite reductase	No	1.7.7.1	NO2 + 6 Fd(Red) -> NH3 + 6 Fd(Ox)					
CAC0095	*hemA*	glutamyl-tRNA reductase	No	1.2.1.70	LGLU + NADPH + ATP -> GLU1SA + AMP + NADP + PPi					

CAC0096	*hemW*	precorrin-2 oxidase / ferrochelatase	No	1.3.1.76/4.99.1.4	PRCR2 + NAD -> SHCL + NADH	Fe2 + SHCL -> SHEME
CAC0097	*hemC*	hydroxymethylbilane synthase	No	2.5.1.61	4 PPBNG -> HMBIL + 4 NH3	
CAC0098	*hemD*	uroporphyrinogen III synthase	No	2.1.1.107/4.2.1.75	HMBIL -> UPPG3	2 AMET + UPPG3 -> 2 AHCYS + PRCR2
CAC0099	*hemL*	glutamate-1-semialdehyde 2,1-aminomutase	No	5.4.3.8	GLU1SA -> 5AOP	
CAC0100	*hemB*	porphobilinogen synthase	No	4.2.1.24	2 5AOP -> PPBNG	
CAC0102		O-acetylhomoserine (thiol)-lyase	No	2.5.1.49	ACHMS + S -> LHCYS + AC	
CAC0103	*cysC*	adenylylsulfate kinase	No	2.7.1.25	APS + ATP -> PAPS + ADP	
CAC0104		adenylylsulfate reductase, subunit A	Yes	1.8.99.2	SO3 + 3 NADPH -> S + 3 NADP	
CAC0109	*cysD*	sulfate adenylyltransferase subunit 2	Yes	2.7.7.4	SO4 + ATP -> APS + PPi	
CAC0110	*cysN*	adenylylsulfate kinase / sulfate adenylyltransferase subunit 1	Yes	2.7.1.25/2.7.7.4	SO4 + ATP -> APS + PPi	APS + ATP -> PAPS + ADP
CAC0116		carbone-monoxide dehydrogenase, beta chain	No	1.2.99.2	CO2 + MECORR -> ACCOA + CORR	
CAC0154	*mtlA*	PTS system, mannitol-specific IIBC component (gene MtlA)	Yes	2.7.1.69	MNL(Ext) + PEP -> MNL1P + PYR	
CAC0156	*mltF*	PTS system, mannitol-specific IIA domain (Ntr-type) (gene MltF)	Yes	2.7.1.69	MNL(Ext) + PEP -> MNL1P + PYR	

CAC0157	*mtlD*	mannitol-1-phosphate 5-dehydrogenase	No	1.1.1.17	MNL1P + NAD <-> F6P + NADH	
CAC0158	*glmS*	glucosamine--fructose-6-phosphate aminotransferase (isomerizing)	No	2.6.1.16	F6P + LGLN -> GAM6P + LGLU	
CAC0187	*nagB*	glucosamine-6-phosphate isomerase (glucosamine-6-phosphate	No	3.5.99.6	F6P + NH3 <-> GAM6P	
CAC0188	*nagA*	N-acetylglucosamine-6-phosphate deacetylase (gene nagA)	No	3.5.1.25	GAM6P + AC <-> ACGAM6P	
CAC0217	*pheA*	prephenate dehydrotase (pheA)	No	4.2.1.51	PPHN -> PHPYR + CO2	
CAC0232	*fruB*	1-phosphofructokinase (fructoso 1-phosphate kinase)	No	2.7.1.56	F1P + ATP <-> FDP + ADP	
CAC0253	*nifH*	nitrogenase iron protein (nitrogenase component II) gene nifH	Yes	1.18.6.1	N2 + 16 ATP + 8 Fd(Red) -> 16 Pi + 16 ADP + 8 Fd(Ox) + 2 NH3 + H2	
CAC0256	*nifD*	nitrogenase molybdenum-iron protein, alpha chain (nitrogenase component I) gene nifD	Yes	1.18.6.1	N2 + 16 ATP + 8 Fd(Red) -> 16 Pi + 16 ADP + 8 Fd(Ox) + 2 NH3 + H2	
CAC0257	*nifK*	nitrogenase molibdenum-iron protein, beta chain, gene nifK	Yes	1.18.6.1	N2 + 16 ATP + 8 Fd(Red) -> 16 Pi + 16 ADP + 8 Fd(Ox) + 2 NH3 + H2	
CAC0263	*serB*	phosphoserine phosphatase related protein	No	3.1.3.3	LPSER -> LSER + Pi	
CAC0267	*ldh*	L-lactate dehydrogenase	No	1.1.1.27	PYR + NADH <-> LAC + NAD	2HBUT + NAD -> 2OBUT + NADH
CAC0273		2-isopropylmalate synthase	No	2.3.3.13	ACCOA + 3MOB -> 2IPPMAL + COA	
CAC0274	*ansB*	aspartate ammonia-lyase (aspartase) gene ansB(aspA)	No	4.3.1.1	LASP -> FUM + NH3	

CAC0278		aspartate kinase	No	2.7.2.4	LASP + ATP -> 4PASP + ADP				
CAC0282		cytosine/guanine deaminase related protein	No	3.5.4.3	GUA -> XAN + NH3				
CAC0316	*argF, argI*	ornithine carbomoyltransferase	No	2.1.3.3	CBP + LORN <-> LCITR + Pi				
CAC0329	*spoVD*	sporulation specific penicillin-binding protein	No	2.4.1.129	1.064 UAMR + 1.064 UACGAM + 1.106 LALA + 1.106 LGLU + 1.106 DALADALA + 1.106 26DAP-M + 4.425 ATP -				
CAC0368		4 animobutyrate aminotransferase	No	2.6.1.19	4ABUT + AKG <-> SUCCSA + LGLU				
CAC0385		beta-glucosidase	No	3.2.1.21	CLB(Ext) -> 2 bDGLC(Ext)				
CAC0390		cystathionine gamma-synthase	No	2.5.1.48	SUCHMS + LCYS -> CYST + SUCC	SUCHMS <-> 2OBUT + SUCC + NH3	CYST + AC <-> ACHMS + LCYS	ACHMS + S -> LHCYS + AC	SUCHMS + S <-> LHCYS + SUCC
CAC0391		cystathionine beta-lyase	No	4.4.1.8	CYST -> LHCYS + NH3 + PYR	S + PYR + NH3 -> LCYS			
CAC0394	*kdgA*	deoxyphosphogluconate aldolase (gene kdgA)	No	4.1.2.14/4.1.3.16	2DDG6P <-> GA3P + PYR				
CAC0395	*kdgK*	2-keto-3-deoxygluconate kinase (gene kdgK)	No	2.7.1.45	2DDGLCN + ATP -> 2DDG6P + ADP				
CAC0423		fusion: PTS system, beta-glucosides specific IIABC component	Yes	2.7.1.69	SUCR(Ext) + PEP -> SUC6P + PYR				
CAC0424		fructokinase	No	2.7.1.4	FRU + ATP -> F6P + ADP				
CAC0425	*sacA*	sucrase-6-phosphate hydrolase (gene sacA)	No	3.2.1.26	SUC6P -> FRU + G6P				

CAC0434	*ispF*	putative 2-C-methyl-D-erythritol 2,4-cyclodiphosphate synthase	No	4.6.1.12	CDPMERY2P -> MERYcDP + CMP			
CAC0480	*nrdD*	oxygen-sensitive ribonucleoside-triphosphate reductase nrdD	No	1.17.4.2	ATP + TRD(Red) -> dATP + TRD(Ox)	GTP + TRD(Red) -> dGTP + TRD(Ox)	CTP + TRD(Red) -> dCTP + TRD(Ox)	UTP + TRD(Red) -> dUTP + TRD(Ox)
CAC0484		phosphomannomutase	No	5.4.2.10	GAM6P -> GAM1P			
CAC0492	*alr*	alanine racemase	No	5.1.1.1	LALA <-> DALA			
CAC0501	*bacA*	undecaprenyl-diphosphatase	No	3.6.1.27	1.064 UAMR + 1.064 UACGAM + 1.106 LALA + 1.106 LGLU + 1.106 DALADALA + 1.106 26DAP-M + 4.425 ATP - [illegible]			
CAC0510	*murB*	UDP-N-acetylenolpyruvoylglucosamine reductase (murB)	No	1.1.1.158	UACCG + NADPH -> UAMR + NADP			
CAC0517	*pfk*	6-phosphofructokinase	No	2.7.1.11	F6P + ATP -> FDP + ADP	TAG6P + ATP <-> TAGDP + ADP		
CAC0518	*pykA*	pyruvate kinase (pykA)	No	2.7.1.40	PEP + ADP -> PYR + ATP	dATP + PYR -> dADP + PEP	GDP + PEP <-> GTP + PYR	dGTP + PYR <-> dGDP + PEP
CAC0519	*pyrC*	dihydroorotase	No	3.5.2.3	CBASP <-> DHOR-S			
CAC0523		SAM-dependent methyltransferase related to tRNA(uracyl-5-)-methyltransferase (trmA family)	No	2.1.1.-	LHIS + AMET -> MLHIS + AHCYS			
CAC0532		PTS system, maltose-specific enzyme IIBC component	No	2.7.1.69	MALT(Ext) + PEP -> MALT6P + PYR	ARBT(Ext) + PEP -> ARPT6P + PYR		
CAC0533	*glvA*	maltose-6'-phosphate glucosidase (glvA)	No	3.2.1.122	MALT6P -> GLC + G6P			
CAC0534	*pps*	phosphoenolpyruvate synthase (gene pps)	No	2.7.9.2	PYR + ATP -> PEP + AMP + Pi			

CAC0566		malate dehydrogenase	No	1.1.1.37	OAA + NADH <-> MAL + NAD	
CAC0568	*asd*	aspartate semialdehyde dehydrogenase (gene asd)	No	1.2.1.11	4PASP + NADPH -> ASPSA + Pi + NADP	GTP -> FOR + 25DRAPP + PPi
CAC0570		PTS system, glucose-specific IIABC component	No	2.7.1.69	GLC(Ext) + PEP -> G6P + PYR	GLC + ATP <-> G6P + ADP
CAC0578	*metH*	cobalamine-dependent methionine synthase I (methyltransferase and cobalamine-binding domain)	No	2.1.1.13	LHCYS + 5MTHF -> LMET + THF	
CAC0582		cobyrinic acid a,c-diamide synthase CobB/CbiA (CBIB protein)	No	6.3.1.10	ATP + ACBRNHA + 1APROH -> ADP + Pi + ACBA	ACBRNHA + APROHP + ATP -> ACBAP + ADP + Pi
CAC0584		precorrin-6B methylase 1 CobL1/CbiE	No		CDHPRCR6 + AMET -> CPRCR7 + AHCYS	
CAC0590	*ribD*	diaminohydroxyphosphoribosylaminopyrimidine deaminase / 5-amino-6-(5-phosphoribosylamino)uracil reductase	No	3.5.4.26/1.1.1.193	25DRAPP -> 5APRBU + NH3	5APRBU + NADP -> 5APRU + NADPH
CAC0591	*ribB*	riboflavin synthase alpha chain	No	2.5.1.9	2 DMLZ -> RIBFLA + 4R5AU	
CAC0592	*ribA*	riboflavin biosynthes protein RIBA (GTPcyclohydrolase/3,4-dihydroxy-2-butanone 4-phosphate synthase)	No	3.5.4.25	DRU5P -> DB4P + FOR	
CAC0593	*ribH*	riboflavin synthase beta chain	No		4R5AU + DB4P -> DMLZ + Pi	
CAC0608	*lisA*	diaminopimelate decarboxilase, lisA	No	4.1.1.20	26DAP-M -> LLYS + CO2	
CAC0673		L-serine dehydratase, beta chain	Yes	4.3.1.17	LSER -> PYR + NH3	
CAC0674		L-serine dehydratase, alpha chain	Yes	4.3.1.17	LSER -> PYR + NH3	

CAC0676	*pssA*	phosphatidylserine synthase	No	2.7.8.8	CDP-DAG + LSER -> CMP + PS
CAC0682	*nrgA*	ammonium transporter (membrane protein nrgA)	No		NH3(Ext) -> NH3
CAC0686		spore cortex-lytic enzyme prepeptide; peptodoglycan-binding domain	No	3.5.1.28	1.064 UAMR + 1.064 UACGAM + 1.106 LALA + 1.106 LGLU + 1.106 DALADALA + 1.106 26DAP-M + 4.425 ATP -
CAC0687	*cysE*	serine acetyltransferase	No	2.3.1.30	LSER + ACCOA -> ACSER + COA
CAC0709	*gapC*	glyceraldehyde 3-phosphate dehydrogenase, gene gapC	No	1.2.1.12	GA3P + Pi + NAD <-> 13DPG + NADH
CAC0710	*pgk*	phosphoglycerate kinase	No	2.7.2.3	13DPG + ADP <-> 3PG + ATP
CAC0711	*tpi*	triosephosphate isomerase (TIM)	No	5.3.1.1	DHAP <-> GA3P
CAC0712	*pgm*	2,3-bisphosphoglycerate-independent phosphoglycerate mutase gene	No	5.4.2.1	3PG <-> 2PG
CAC0713	*eno*	enolase	No	4.2.1.11	2PG <-> PEP
CAC0726		ribose 5-phosphate isomerase RpiB	No	5.3.1.6	R5P <-> DRU5P
CAC0737		NADP-specific glutamate dehydrogenase	No	1.4.1.4	AKG + NH3 + NADPH <-> LGLU + NADP
CAC0764		NADPH-dependent glutamate synthase beta chain	Yes	1.4.1.13	LGLN + AKG + NADPH -> 2 LGLU + NADP
CAC0770		glycerol uptake facilitator protein, permease	No		GLYC <-> GLYC(Ext)

CAC0792		D-amino acid aminotransferase	No	2.6.1.21	PYR + DGLU <-> AKG + DALA					
CAC0794		nucleoside-diphosphate-sugar epimerase (UDP-glucose 4-epimerase)	No	5.1.3.2	UDPGAL <-> UDPGLC	TDPGLC <-> TDPGAL				
CAC0798		phosphatidylserine synthase	No	2.7.8.8	CDP-DAG + LSER -> CMP + PS					
CAC0799	*psd*	phosphatidylserine decarboxylase	No	4.1.1.65	PS -> PE + CO2					
CAC0814		3-oxoacyl-[acyl-carrier-protein] synthase III	No	2.3.1.180	ACCOA + ACP <-> ACACP + COA	ACACP + 6 MALACP + 12 NADPH -> 12 NADP + C140-ACP + 6 CO2 + 6 ACP	ACACP + 7 MALACP + 14 NADPH -> 14 NADP + C160-ACP + 7 CO2 + 7 ACP	ACACP + 7 MALACP + 13 NADPH -> 13 NADP + C161-ACP + 7 CO2 + 7 ACP	ACACP + 8 MALACP + 16 NADPH -> 16 NADP + C180-ACP + 8 CO2 + 8 ACP	ACACP + 8 MALACP + 15 NADPH -> 15 NADP + C181-ACP + 8 CO2 + 8 ACP
CAC0819		phosphoribosylpyrophosphate synthetase	No	2.7.6.1	R5P + ATP -> PRPP + AMP					
CAC0827		fructose-bisphosphate aldolase	No	4.1.2.13	FDP -> DHAP + GA3P	F1P -> DHAP + GLYALD				
CAC0857		glucan phosphorylase	No	2.4.1.1	Glycogen + Pi -> G1P					
CAC0869		thioredoxine reductase	No	1.8.1.9	TRD(Ox) + NADPH -> TRD(Red) + NADP					
CAC0887	*adeC*	adenine deaminase	No	3.5.4.2	ADE -> HXAN + NH3					
CAC0892		phospho-2-dehydro-3-deoxyheptonate aldolase	No	2.5.1.54	PEP + E4P -> 2DDA7P + Pi					
CAC0893		prephenate dehydrogenase	No	1.3.1.12	PPHN + NAD -> 34HPP + CO2 + NADH					
CAC0894	*aroB*	3-dehydroquinate synthetase	No	4.2.3.4	2DDA7P -> 3DHQ + Pi					

CAC0895	*aroA*	5-enolpyruvylshikimate-3-phosphate synthase	No	2.5.1.19	SKM3P + PEP <-> 3PSME + Pi				
CAC0896	*aroC*	chorismate synthase	No	4.2.3.5	3PSME -> CHOR + Pi				
CAC0897	*aro*	fusion: chorismate mutase and shikimate 5-dehydrogenase	No	1.1.1.25	3DHSK + NADPH <-> SKM + NADP				
CAC0898	*aroK*	shikimate kinase	No	2.7.1.71	SKM + ATP -> SKM3P + AD				
CAC0899		3-dehydroquinate dehydratase II	No	4.2.1.10	3DHQ <-> 3DHSK				
CAC0930	*metB*	cystathionine gamma-synthase	No	2.5.1.48	SUCHMS + LCYS -> CYST + SUCC	SUCHMS <-> 2OBUT + SUCC + NH3	CYST + AC <-> ACHMS + LCYS	ACHMS + S -> LHCYS + AC	SUCHMS + S <-> LHCYS + SUCC
CAC0931		cysteine synthase	No	2.5.1.47	S + ACSER -> LCYS + AC				
CAC0936	*hisG*	ATP phosphoribosyltransferase	No	2.4.2.17	PRPP + ATP -> PRBATP + PPi				
CAC0937	*hisD*	histidinol dehydrogenase	No	1.1.1.23	HISTD + NAD -> HISTDAL + NADH	HISTDAL + NAD -> LHIS + NADH			
CAC0938	*hisB*	imidazoleglycerol-phosphate dehydratase	No	4.2.1.19	EIG3P -> IMACP	HISP -> HISTD + Pi			
CAC0939	*hisH*	glutamine amidotransferase	No	2.4.2.-	PRLP + LGLN -> AICAR + LGLU + EIG3P				
CAC0940	*hisA*	phosphoribosylformimino-5-aminoimidazole carboxamide ribonucleotide (ProFAR) isomerase	No	5.3.1.16	PRFP -> PRLP				
CAC0941	*hisF*	imidazoleglycerol-phosphate synthase cyclase	No	4.1.3.-	PRLP + LGLN -> AICAR + LGLU + EIG3P				

CAC0942	*hisI_1*	phosphoribosyl-AMP cyclohydrolase	No	3.5.4.19	PRBAMP -> PRFP	
CAC0943	*his_2*	phosphoribosyl-ATP pyrophosphohydrolase	No	3.6.1.31	PRBATP -> PRBAMP + PPi	
CAC0944	*tkt*	transketolase	No	2.2.1.1	F6P + GA3P <-> E4P + DXU5P	R5P + DXU5P <-> S7P + GA3P
CAC0963	*bacA*	undecaprenyl-diphosphatase	No	3.6.1.27	1.064 UAMR + 1.064 UACGAM + 1.106 LALA + 1.106 LGLU + 1.106 DALADALA + 1.106 26DAP-M + 4.425 ATP -> PEPTIDO + 1.106 DALA + 1.106 UDP + 1.106 UMP + 4.425 ADP + 4.425 Pi	
CAC0965		1-acyl-sn-glycerol-3-phosphate acyltransferase	No	2.3.1.51	1-Acyl-GLYC3P + 0.073 C140-ACP + 0.521 C160-ACP + 0.065 C161-ACP + 0.036 C180-ACP + 0.102 C181-ACP + 0.022 C17CYC-ACP + 0.181 C19CYC-ACP -> PA + ACP	
CAC0971	*citB*	aconitase A	No	4.2.1.3	CIT <-> ICIT	
CAC0972	*citC*	isocitrate dehydrogenase	No	1.1.1.41	ICIT + NAD <-> AKG + CO2 + NADH	
CAC0973	*argG*	argininosuccinate synthase	No	6.3.4.5	LASP + ATP + LCITR -> AMP + PPi + ARGSUC	
CAC0974	*argH*	argininosuccinate lyase	No	4.3.2.1	ARGSUC -> FUM + LARG	
CAC0980	*pflB*	pyruvate-formate lyase	No	2.3.1.54	PYR + COA -> ACCOA + FOR	2OBUT + COA -> PROCOA + FOR
CAC0990	*gltX*	glutamyl-tRNA synthetase	No	6.1.1.17	LGLU + NADPH + ATP -> GLU1SA + AMP + NADP + PPi	

CAC0998		homoserine dehydrogenase	No	1.1.1.3	ASPSA + NADPH <-> LHMS + NADP			
CAC0999	*thrC*	threonine synthase	No	4.2.3.1	PHOM -> LTHR + Pi			
CAC1001		aspartate aminotransferase	No	2.6.1.1	AKG + LASP <-> OAA + LGLU	PHPYR + LGLU <-> LPHE + AKG	34HPP + LGLU <-> LTYR + AKG	
CAC1002		nicotinic acid phosphoribosyltransferase	No	2.4.2.11	NA + PRPP -> NAMN + PPi			
CAC1003		superfamily I DNA helicase (rep-like helicase)	No	3.6.1.-	AHETHPDHPTP -> DHNPP + PPi	DHNPP -> DHNP + Pi		
CAC1009	*coaE*	P-loop kinase (uridine kinase family)	No	2.7.1.24	ATP + DPCOA -> ADP + COA			
CAC1023	*nadC*	nicotinate-nucleotide pyrophosphorylase	No	2.4.2.19	QULN + PRPP -> NAMN + PPi + CO2			
CAC1024	*nadB*	aspartate oxidase	No	1.4.3.16	LASP + O2 -> OAA + NH3 + H2O2			
CAC1025	*nadA*	quinolinate synthase	No		LASP + FOR + ACCOA -> QULN			
CAC1036	*pykA*	pyruvate kinase	No	2.7.1.40	PEP + ADP -> PYR + ATP	dATP + PYR -> dADP + PEP	GDP + PEP <-> GTP + PYR	
CAC1047		ribonucleotide reductase, vitamin B12-dependent	No	1.17.4.1	ADP + TRD(Red) -> dADP + TRD(Ox)	GDP + TRD(Red) -> dGDP + TRD(Ox)	CDP + TRD(Red) -> dCDP + TRD(Ox)	UDP + TRD(Red) -> dUDP + TRD(Ox)
CAC1050	*nadE*	NH(3)-dependent NAD(+) synthetase	No	6.3.5.1	LGLN + ATP + DNAD -> LGLU + AMP + PPi + NAD			
CAC1054		arginase	No	3.5.3.1	LARG -> LORN + UREA			

CAC1075		beta-glucosidase family protein	No	3.2.1.21	CLB(Ext) -> 2 bDGLC(Ext)			
CAC1084		beta-glucosidase family protein	No	3.2.1.21	CLB(Ext) -> 2 bDGLC(Ext)			
CAC1088	*glpX*	GlpX-like protein (Fructose-1,6-bisphosphatase related protein)	No	3.1.3.11	FDP -> F6P + Pi			
CAC1090		5-formyltetrahydrofolate cyclo-ligase	No	6.3.3.2	ATP + 5FTHF -> ADP + Pi + METHF			
CAC1209	*nrdD*	anaerobic ribonucleotide reductase	No	1.17.4.2	ATP + TRD(Red) -> dATP + TRD(Ox)	GTP + TRD(Red) -> dGTP + TRD(Ox)	CTP + TRD(Red) -> dCTP + TRD(Ox)	UTP + TRD(Red) -> dUTP + TRD(Ox)
CAC1210	*dut*	deoxyuridine 5'triphosphate nucleotidohydrolase (DUPTase)	No	3.6.1.23	dUTP -> dUMP + PPi			
CAC1234	*pheB*	chorismate mutase PheB of B.subtilis ortholog	No	5.4.99.5	CHOR <-> PPHN			
CAC1235	*thrB*	homoserine kinase (thrB)	No	2.7.1.39	LHMS + ATP -> PHOM + ADP			
CAC1262	*nadD*	predicted nucleotidyltransferases of NarD/TagD family (N-term. domain) , yqeJ ortholog	No	2.7.7.18	ATP + NAMN -> PPi + DNAD	ATP + NMN -> PPi + NAD		
CAC1294	*dgkA, pgpB*	diacylglycerol kinase (dgkA) fused to phosphatase B domain (pgpB)	No	2.7.1.107	ATP + 1,2-Diacyl-GLYC -> ADP + PA			
CAC1319	*glpF*	glycerol uptake facilitator protein, GLPF	No		GLYC <-> GLYC(Ext)			
CAC1321	*glpK*	glycerol kinase, GLPK	No	2.7.1.30	ATP + GLYC -> ADP + GLYC3P			
CAC1341	*araD*	ribulose-5-phosphate 4-epimerase family protein	No	5.1.3.4	LRU5P <-> DXU5P			

CAC1342	*araA*	L-arabinose isomerase	No	5.3.1.4	LARAB <-> LRBL		
CAC1344	*xylB*	sugar kinase, possible xylulose kinase	No	2.7.1.17	DXYLU + ATP <-> DXU5P + ADP		
CAC1346	*araA*	L-arabinose isomerase	No	5.3.1.4	LARAB <-> LRBL		
CAC1347		transaldolase	No	2.2.1.2	S7P + GA3P <-> E4P + F6P		
CAC1348		transketolase, TKT	No	2.2.1.1	F6P + GA3P <-> E4P + DXU5P	R5P + DXU5P <-> S7P + GA3P	
CAC1349	*galM*	aldose-1-epimerase	No	5.1.3.3	bDGLC <-> GLC		
CAC1353		phosphotransferase system IIC component, possibly N-acetylglucosamine-specific	Yes	2.7.1.69	ACGAM + PEP -> ACGAM6P + PYR		
CAC1354		PTS system, N-acetylglucosamine-specific IIA component, putative	Yes	2.7.1.69	ACGAM + PEP -> ACGAM6P + PYR		
CAC1369	*hisC*	histidinol-phosphate aminotransferase	No	2.6.1.9	IMACP + LGLU <-> HISP + AKG	PHPYR + LGLU <-> LPHE + AKG	34HPP + LGLU <-> LTYR + AKG
CAC1370	*cbiG*	cobalamin biosynthesis protein CbiG	No		CPRCR5A -> CPRCR5B + ACAL		
CAC1372	*cobT*	cobalamin biosynthesis enzyme CobT	No	2.4.2.21	NAMN + DMBZID -> NA + 5PRDMBZ		
CAC1373	*cbiK*	anaerobic Cobalt chelatase, cbiK	No	4.99.1.3	SHCL + COBALT -> CPRCR2		
CAC1374	*cbiP*	cobyric acid synthase CbiP	No	6.3.5.10	ACBRNDA + 4 LGLN + 4 ATP -> ACBRNHA + 4 GLU + 4 Pi + 4 ADP		

CAC1375	*cobB*	cobyrinic acid a,c-diamide synthase CobB	No	6.3.1.-	CBRN + 2 LGLN + 2 ATP -> CBRNDA + 2 LGLU + 2 ADP + 2 P		
CAC1376	*cbiC, cobH*	precorrin isomerase, cbiC	No	5.4.1.2	CPRCR8 -> CBRN	PRCR8 -> HGBRN	
CAC1377	*cbiD*	cobalamin biosynthesis protein CbiD	No		CPRCR5B + AMET -> CPRCR6 + AHCYS		
CAC1378	*cbiT*	precorrin-6B methylase CbiT	No		CPRCR7 + AMET -> CPRCR8 + AHCYS + CO2		
CAC1379	*cobI, cbiL*	precorrin-2 methylase CobI/CbiL	No	2.1.1.151	CPRCR2 + AMET -> CPRCR3 + AHCYS		
CAC1380	*cbiF, cobM*	precorrin-4 methylase cbiF	No	2.1.1.133	CPRCR4 + AMET -> CPRCR5A + AHCYS	AMET + PRCR4 -> AHCYS + PRCR5	
CAC1381	*cbiJ, cobK*	precorrin-6x reductase	No	1.3.1.54	CPRCR6 + NADPH -> CDHPRCR6 + NADP	PRCR6A + NADPH -> PRCR6B + NADP	
CAC1382	*cbiH, cobJ*	precorrin-3 methylase	No	2.1.1.131	CPRCR3 + AMET -> CPRCR4 + AHCYS	AMET + PRCR3B -> AHCYS + PRCR4	
CAC1383	*CobU, CobP*	adenosyl cobinamide kinase/adenosyl cobinamide phosphate	No	2.7.1.156/2.7.7.62	ACBA + ATP -> ACBAP + ADP	ACBA + GTP -> ACBAP + GDP	ACBAP + GTP -> AGDPCBA + PPi
CAC1384	*cobS*	cobalamin-5-phosphate synthase	No	2.7.8.26	AGDPCBA + ARBZL -> CACO + GMP		
CAC1385	*cobC*	alpha-ribazole-5'-phosphate phosphatase, CobC	No	3.1.3.73	ARBZL5P -> ARBZL + Pi		
CAC1390	*purE*	phosphoribosylcarboxyaminoimidazole (NCAIR) mutase	No	4.1.1.21	AIR + HCO3 <-> PRAIC		
CAC1391	*purC*	phosphoribosylaminoimidazolesuccinocarboxamide (SAICAR) synthase	No	6.3.2.6	PRAIC + LASP + ATP -> SAICAR + ADP + Pi		

CAC1392	*purF*	glutamine phosphoribosylpyrophosphate amidotransferase	No	2.4.2.14	PRPP + LGLN -> PRAM + PPi + LGLU	
CAC1393	*purM*	phosphoribosylaminoimidazol (AIR) synthetase	No	6.3.3.1	FGAM + ATP -> AIR + ADP + Pi	
CAC1394	*purN*	folate-dependent phosphoribosylglycinamide formyltransferase	No	2.1.2.2	GAR + 10FTHF -> FGAR + THF	
CAC1395	*purH*	AICAR transformylase/IMP cyclohydrolase	No	2.1.2.3/3.5.4.10	AICAR + 10FTHF -> FPRICA + THF	FPRICA <-> IMP
CAC1396	*purD*	phosphoribosylamine-glycine ligase	No	6.3.4.13	PRAM + GLY + ATP -> GAR + ADP + Pi	
CAC1405	*bglA*	beta-glucosidase	No	3.2.1.21	CLB(Ext) -> 2 bDGLC(Ext)	
CAC1427	*gabT*	4-aminobutyrate aminotransferase (PLP-dependent)	No	2.6.1.19	4ABUT + AKG <-> SUCCSA + LGLU	
CAC1429	*galE*	UDP-glucose 4-epimerase	No	5.1.3.2	UDPGAL <-> UDPGLC	TDPGLC <-> TDPGAL
CAC1431	*rpiA*	ribose 5-phosphate isomerase	No	5.3.1.6	R5P <-> DRU5P	
CAC1432		undecaprenyl pyrophosphate synthase related enzyme	No	2.5.1.31	FRDP + IPDP -> GGRDP + PPi	GGRDP + 7 IPDP -> UDCPDP + 7 PPi
CAC1435		S-adenosylmethionine-dependent methyltransferases	No	2.1.1.-	LHIS + AMET -> MLHIS + AHCYS	
CAC1457		PTS system, fructose(mannose)-specific IIA component	Yes	2.7.1.69	FRU(Ext) + PEP -> PYR + F1P	MAN(Ext) + PEP -> MAN6P + PYR
CAC1458		PTS system, fructose(mannose)-specific IIB	Yes	2.7.1.69	FRU(Ext) + PEP -> PYR + F1P	MAN(Ext) + PEP -> MAN6P + PYR

CAC1459		PTS system, fructose(mannose)-specific IIC	Yes	2.7.1.69	FRU(Ext) + PEP -> PYR + F1P	MAN(Ext) + PEP -> MAN6P + PYR	
CAC1460		PTS system, fructose(mannose)-specific IID	Yes	2.7.1.69	FRU(Ext) + PEP -> PYR + F1P	MAN(Ext) + PEP -> MAN6P + PYR	
CAC1479	*ilvE*	branched-chain-amino-acid transaminase (ilvE)	No	2.6.1.42	3MOP + LGLU -> LILE + AKG	3MOB + LGLU -> LVAL + AKG	4MOP + LGLU -> LLEU + AKG
CAC1513	*asrA*	anaerobic sulfite reductase (Fe-S subunit)	Yes		SO3 + 3 NADPH -> S + 3 NADP		
CAC1514	*asrB*	anaerobic sulfite reductase subunit B	Yes		SO3 + 3 NADPH -> S + 3 NADP		
CAC1515	*asrC*	anaerobic sulfite reduction protein C, reductase	Yes		SO3 + 3 NADPH -> S + 3 NADP		
CAC1523		fructokinase	No	2.7.1.4	FRU + ATP -> F6P + ADP		
CAC1549	*bsaA*	glutathione peroxidase	No	1.11.1.9	H2O2 + 2 GTH(Red) -> GTH(Ox)		
CAC1570	*bsaA*	glutathione peroxidase	No	1.11.1.9	H2O2 + 2 GTH(Red) -> GTH(Ox)		
CAC1571		glutathione peroxidase	No	1.11.1.9	H2O2 + 2 GTH(Red) -> GTH(Ox)		
CAC1572		fructose-1,6-bisphosphatase	No	3.1.3.11	FDP -> F6P + Pi		
CAC1589	*malS*	malic enzyme	No	1.1.1.38/1.1.1.40	MAL + NAD -> PYR + CO2 + NADH	MAL + NADP -> PYR + CO2 + NADPH	
CAC1596	*malS*	malate dehydrogenase (oxaloacetate-decarboxylating)	No	1.1.1.38	MAL + NAD -> PYR + CO2 + NADH		

CAC1625		phosphoserine phosphatase family enzyme	No	3.1.3.3	LPSER -> LSER + Pi	
CAC1655	*purQ, purL*	bifunctional enzyme phosphoribosylformylglycinamidine (FGAM) synthase (synthetase domain/glutamine [illegible]	No	6.3.5.3	FGAR + LGLN + ATP -> FGAM + LGLU + ADP + Pi	
CAC1652	*aspA*	aspartate ammonia-lyase	No	4.3.1.1	LASP -> FUM + NH3	
CAC1664	*glgP*	glycogen phosphorylase	No	2.4.1.1	Glycogen + Pi -> G1P	
CAC1673	*gltA*	large subunit of NADH-dependent glutamate synthase	Yes	1.4.1.13	LGLN + AKG + NADPH -> 2 LGLU + NADP	
CAC1674	*gltB*	small subunit of NADPH-dependent glutamate synthase	Yes	1.4.1.13	LGLN + AKG + NADPH -> 2 LGLU + NADP	
CAC1705		periplasmic phosphate-binding protein	Yes	3.6.3.27	Pi(Ext) + ATP -> ADP + 2 Pi	
CAC1706		phosphate permease	Yes	3.6.3.27	Pi(Ext) + ATP -> ADP + 2 Pi	
CAC1707		permease component of ATP-dependent phosphate uptake system	Yes	3.6.3.27	Pi(Ext) + ATP -> ADP + 2 Pi	
CAC1708		ATPase component of ABC-type phosphate transport system	Yes	3.6.3.27	Pi(Ext) + ATP -> ADP + 2 Pi	
CAC1712	*gpsA*	glycerol 3-phosphate dehydrogenase	No	1.1.1.94	GLYC3P + NAD <-> DHAP + NADH	GLYC3P + NADP <-> DHAP + NADPH
CAC1714	*ansA*	L-asparaginase	No	3.5.1.1	LASN -> LASP + NH3	
CAC1718		guanylate kinase, YLOD B.subtilis ortholog	No	2.7.4.8	GMP + ATP <-> GDP + ADP	

CAC1720		flavoprotein involved in panthothenate metabolism, YLOI B.subtilis ortholog	No	4.1.1.36/6.3.2.5	ATP + 4PPAN + LCYS -> ADP + Pi + 4PPCYS	CTP + 4PPAN + LCYS -> CDP + Pi + 4PPCYS	4PPCYS -> PAN4P + CO2
CAC1730		pentose-5-phosphate-3-epimerase	No	5.1.3.1	DXU5P <-> DRU5P		
CAC1738	*kdtB*	phosphopantetheine adenylyltransferase	No	2.7.7.3	ATP + PAN4P -> PPi + DPCOA		
CAC1742	*pta*	phosphate acetyltransferase	No	2.3.1.8	ACCOA + Pi -> ACTP + COA	PROCOA + Pi -> PROP + COA	
CAC1743	*askA*	acetate kinase	No	2.7.2.1	ACTP + ADP -> AC + ATP	PROP + ADP -> PROPAC + ATP	
CAC1780		nicotinic acid phosphoribosyltransferase	No	2.4.2.11	NA + PRPP -> NAMN + PPi		
CAC1782	*nadE*	NH(3)-dependent NAD(+) synthase (nadE) fused to amidohydrolase domain	No	6.3.5.1	LGLN + ATP + DNAD -> LGLU + AMP + PPi + NAD		
CAC1789	*smbA, pyrH*	uridylate kinase	No	2.7.4.22	UMP + ATP <-> UDP + ADP		
CAC1791		undecaprenyl pyrophosphate synthase	No	2.5.1.31	FRDP + IPDP -> GGRDP + PPi		
CAC1792	*cdsA*	CDP-diglyceride synthetase	No	2.7.7.41	PA + CTP -> CDP-DAG + PPi	GGRDP + 7 IPDP -> UDCPDP + 7 PPi	
CAC1795		1-deoxy-D-xylulose 5-phosphate reductoisomerase	No	1.1.1.267	dXYLU5P + NADPH -> MERYTH4P + NADP		
CAC1797	*gcpE*	1-hydroxy-2-methyl-2-(E)-butenyl 4-diphosphate synthase	No	1.17.4.3	MERYcDP + ProDTH -> HMB4DP + ProDS		
CAC1806		riboflavin kinase/FAD synthase	No	2.7.1.26/2.7.7.2	ATP + RIBFLA -> ADP + FMN	ATP + FMN -> PPi + FAD	

CAC1810	*dapG*	Aspartokinase	No	2.7.2.4	LASP + ATP -> 4PASP + ADP					
CAC1814	*pgsA*	phosphatidylglycerophosphate synthase	No	2.7.8.5	CDP-DAG + GLYC3P -> CMP + PGP					
CAC1819	*aspB*	aspartate aminotransferase	No	2.6.1.1	AKG + LASP <-> OAA + LGLU	PHPYR + LGLU <-> LPHE + AKG	34HPP + LGLU <-> LTYR + AKG			
CAC1820		phosphocarrier protein (Hpr)	Yes		All PTS reactions					
CAC1821	*purB*	adenylosuccinate lyase	No	4.3.2.2	SAICAR <-> FUM + AICAR	DCAMP -> AMP + FUM				
CAC1825	*metB*	homoserine trans-succinylase	No	2.3.1.46	LHMS + SUCCOA -> SUCHMS + COA					
CAC1848	*cmk*	cytidylate kinase	No	2.7.4.14	CDP + ADP <-> CMP + ATP	UMP + ATP <-> UDP + ADP	dCDP + ADP <-> dCMP + ATP			
CAC1958		predicted aldo/keto reductase, YTBE/YVGN B.subtilis ortholog	No	1.1.1.21	DXYL + NADPH <-> XOL + NADP					
CAC2008	*pksF*	3-oxoacyl-(acyl-carrier-protein) synthase	No	2.3.1.41	ACCOA + ACP <-> ACACP + COA	ACACP + 6 MALACP + 12 NADPH -> 12 NADP + C140-ACP + 6 CO2 + 6 ACP	ACACP + 7 MALACP + 14 NADPH -> 14 NADP + C160-ACP + 7 CO2 + 7 ACP	ACACP + 7 MALACP + 13 NADPH -> 13 NADP + C161-ACP + 7 CO2 + 7 ACP	ACACP + 8 MALACP + 16 NADPH -> 16 NADP + C180-ACP + 8 CO2 + 8 ACP	ACACP + 8 MALACP + 15 NADPH -> 15 NADP + C181-ACP + 8 CO2 + 8 ACP
CAC2064	*deoD*	purine nucleoside phosphorylase	No	2.4.2.1	ADE + 2DR1P <-> dADN + Pi	dINS + Pi <-> HXAN + 2DR1P	HXAN + R1P <-> INS + Pi	ADN + Pi <-> ADE + R1P	XANT + Pi <-> XAN + R1P	NAMNs + Pi <-> NA + R1P
CAC2065	*deoB*	phosphopentomutase	No	5.4.2.7	R5P <-> R1P					
CAC2075		predicted kinase	No	2.7.1.23	ATP + NAD <-> ADP + NADP					
CAC2077		deoxyxylulose-5-phosphate synthase	No	2.2.1.7	PYR + GA3P -> dXYLU5P + CO2					

CAC2080		predicted geranylgeranyl pyrophosphate synthase	No	2.5.1.10	DMPP + IPDP -> GRDP + PPi	GRDP + IPDP -> FRDP + PPi
CAC2083	*folD*	tetrahydrofolate dehydrogenase/cyclohydrolase, FolD	No	1.5.1.5/3.5.4.9	MLTHF + NADP <-> METHF + NADPH	10FTHF <-> METHF
CAC2117	*pfs*	nucleoside phosphorylase	No	3.2.2.9	AHCYS -> RHCYS + ADE	METADN -> ADE + 5METRIB
CAC2127	*mraY*	phospho-N-acetylmuramoyl-pentapeptide transferase, MraY	No	2.7.8.13	1.064 UAMR + 1.064 UACGAM + 1.106 LALA + 1.106 LGLU + 1.106 DALADALA + 1.106 26DAP-M + 4.425 ATP -> PEPTIDO + 1.106 DALA + 1.106 UDP + 1.106 UMP + 4.425 ADP + 4.425 Pi	
CAC2128	*murF*	UDP-N-acetylmuramoylalanyl-D-glutamyl-2,6-diaminopimelate--D-alanyl-D-alanine ligase	No	6.3.2.10	1.064 UAMR + 1.064 UACGAM + 1.106 LALA + 1.106 LGLU + 1.106 DALADALA + 1.106 26DAP-M + 4.425 ATP -> PEPTIDO + 1.106 DALA + 1.106 UDP + 1.106 UMP + 4.425 ADP + 4.425 Pi	
CAC2129	*murE*	UDP-N-acetylmuramyl tripeptide synthase, MurE	No	6.3.2.13	1.064 UAMR + 1.064 UACGAM + 1.106 LALA + 1.106 LGLU + 1.106 DALADALA + 1.106 26DAP-M + 4.425 ATP -> PEPTIDO + 1.106 DALA + 1.106 UDP + 1.106 UMP + 4.425 ADP + 4.425 Pi	
CAC2137		cation transport P-type ATPase	No	3.6.1.-	AHETHPDHPTP -> DHNPP + PPi	DHNPP -> DHNP + Pi
CAC2138		exopolyphosphatase	No	3.6.1.1	PPi -> 2 Pi	
CAC2227		phosphoserine phosphatase family enzyme	No	3.1.3.3	LPSER -> LSER + Pi	

CAC2229		pyruvate:ferredoxin oxidoreductase	No	1.2.7.-	PYR + COA + Fd(Ox) -> ACCOA + CO2 + Fd(Red)	
CAC2231	*murG*	undecaprenyl-PP-MurNAc-pentapeptide-UDPGlcNAc GlcNAc transferase, MurG	No	2.4.1.227	1.064 UAMR + 1.064 UACGAM + 1.106 LALA + 1.106 LGLU + 1.106 DALADALA + 1.106 26DAP-M + 4.425 ATP -	
CAC2235	*cysK*	cysteine synthase/cystathionine beta-synthase, CysK	No	2.5.1.47	S + ACSER -> LCYS + AC	
CAC2237	*glgC*	ADP-glucose pyrophosphorylase	No	2.7.7.27	G1P + ATP -> ADPGLC + PPi	
CAC2238	*glgC*	ADP-glucose pyrophosphorylase	No	2.7.7.27	G1P + ATP -> ADPGLC + PPi	
CAC2239	*glgA*	glycogen synthase, glgA	No	2.4.1.21	ADPGLC -> ADP + Glycoge	
CAC2243	*asnB*	N-terminal domain of asparagine synthase	No	6.3.5.4	LASP + LGLN + ATP -> LASN + LGLU + AMP + PPi	
CAC2250		UDP-glucose pyrophosphorylase	No	2.7.7.9	G1P + UTP -> UDPGLC + PPi	
CAC2264	*glyA*	glycine hydroxymethyltransferase	No	2.1.2.1	GLY + MLTHF <-> THF + LSER	
CAC2275	*apt*	adenine phosphoribosyltransferase; Apt	No	2.4.2.7	AMP + PPi <-> ADE + PRPP	GMP + PPi <-> GUA + PRPP
CAC2315		DTDP-4-dehydrorhamnose reductase, rfbD ortholog	No	1.1.1.133	GDPoRHAM + NADPH -> GDPRHAM + NADP	TDPoRHAM + NADPH -> TDPRHAM + NADP
CAC2331		DTDP-4-dehydrorhamnose 3,5-epimerase	No	5.1.3.13	TDPDHdGLC -> GDPoRHAM	TDPDHdGLC -> TDPoRHAM
CAC2332	*spsJ*	DTDP-D-glucose 4,6-dehydratase	No	4.2.1.46	TDPGLC -> TDPDHdGLC	

CAC2333	*spsI*	DTDP-glucose pyrophosphorylase	No	2.7.7.24	dTTP + G1P -> TDPGLC + PPi	
CAC2334		UDP-glucose 4-epimerase	No	5.1.3.2	UDPGAL <-> UDPGLC	TDPGLC <-> TDPGAL
CAC2335		UTP-glucose-1-phosphate uridylyltransferase	No	2.7.7.9	G1P + UTP -> UDPGLC + PPi	
CAC2338		lysine decarboxylase	No	4.1.1.18	LLYS -> CDV + CO2	
CAC2378	*dapA*	dihydrodipicolinate synthase	No	4.2.1.52	ASPSA + PYR -> 23DHDP	
CAC2379	*dapB*	dihydrodipicolinate reductase	No	1.3.1.26	23DHDP + NADPH <-> THDP + NADP	
CAC2380		PLP-dependent aminotransferase	No	2.6.1.17	SL2A6O + LGLU <-> SL26DA + AKG	
CAC2381	*dapD*	tetrahydrodipicolinate N-succinyltransferase	No	2.3.1.117	THDP + SUCCOA -> SL2A6O + COA	
CAC2388	*argD*	N-acetylornithine aminotransferase	No	2.6.1.11	ACGLU5SA + LGLU <-> ACORN + AKG	
CAC2389	*argB*	acetylglutamate kinase	No	2.7.2.8	ACGLU + ATP -> ACGLU5P + ADP	
CAC2390	*argC*	N-acetyl-gamma-glutamyl-phosphate reductase	No	1.2.1.38	ACGLU5P + NADPH -> ACGLU5SA + Pi + NADP	
CAC2391	*argJ*	amino-acid N-acetyltransferase / glutamate N-acetyltransferase	No	2.3.1.1/2.3.1.35	LGLU + ACCOA -> ACGLU + COA	ACORN + LGLU <-> LORN + ACGLU
CAC2398	*folC*	folylpolyglutamate synthase	No	6.3.2.17	ATP + DHPT + LGLU -> ADP + Pi + DHF	

CAC2458		2-oxoacid ferredoxin oxidoreductase, beta subunit	Yes	1.2.7.3	Fd(Ox) + AKG + COA <-> Fd(Red) + SUCCOA + CO2				
CAC2459		2-oxoacid ferredoxin oxidoreductase, alpha subunit	Yes	1.2.7.3	Fd(Ox) + AKG + COA <-> Fd(Red) + SUCCOA + CO2				
CAC2498		carbon monoxide dehydrogenase, catalytic subunit (cooS)	No	1.2.99.2	CO2 + MECORR -> ACCOA + CORR				
CAC2499		pyruvate ferredoxin oxidoreductase	No	1.2.7.-	PYR + COA + Fd(Ox) -> ACCOA + CO2 + Fd(Red)				
CAC2601		S-adenosylmethionine decarboxylase	No	4.1.1.50	AMET -> AMETA + CO2				
CAC2602		spermidine synthase	No	2.5.1.16	AMETA + PTRC -> METADN + SPERMD				
CAC2612	*xylB*	xylulose kinase	No	2.7.1.17	DXYLU + ATP <-> DXU5P + ADP	LRBL + ATP <-> LRU5P + ADP			
CAC2613	*glcK*	transcriptional regulators of NagC/XylR family	No	2.7.1.2	bDG6P + ADP <-> ATP + bDGLC				
CAC2614		beta-phosphoglucomutase	No	5.4.2.6	bDG1P <-> bDG6P				
CAC2624	*dapF*	diaminopimelate epimerase	No	5.1.1.7	26DAP-LL <-> 26DAP-M				
CAC2626	*fabG*	possible 3-ketoacyl-acyl carrier protein reductase	No	1.1.1.100	ACACP + 6 MALACP + 12 NADPH -> 12 NADP + C140-ACP + 6 CO2 + 6 ACP	ACACP + 7 MALACP + 14 NADPH -> 14 NADP + C160-ACP + 7 CO2 + 7 ACP	ACACP + 7 MALACP + 13 NADPH -> 13 NADP + C161-ACP + 7 CO2 + 7 ACP	ACACP + 8 MALACP + 16 NADPH -> 16 NADP + C180-ACP + 8 CO2 + 8 ACP	ACACP + 8 MALACP + 15 NADPH -> 15 NADP + C181-ACP + 8 CO2 + 8 ACP
CAC2644	*carB*	carbamoylphosphate synthase large subunit	Yes	6.3.5.5	LGLN + 2 ATP + HCO3 -> LGLU + CBP + 2 ADP + Pi				
CAC2645	*carA*	carbamoylphosphate synthase small subunit	Yes	6.3.5.5	LGLN + 2 ATP + HCO3 -> LGLU + CBP + 2 ADP + Pi				

CAC2650	*pyrD*	dihydroorotate dehydrogenase	No	1.3.3.1	DHOR-S + NAD <-> OROT + NADH		
CAC2652	*pyrF*	orotidine-5'-phosphate decarboxylase	No	4.1.1.23	OROT5P -> UMP + CO2		
CAC2653	*pyrI*	aspartate carbamoyltransferase regulatory subunit	Yes	2.1.3.2	CBP + LASP -> CBASP + Pi		
CAC2654	*pyrB*	aspartate carbamoyltransferase catalytic subunit	Yes	2.1.3.2	CBP + LASP -> CBASP + Pi		
CAC2658	*glnA*	glutamine synthetase type III	No	6.3.1.2	LGLU + ATP + NH3 -> LGLN + ADP + Pi		
CAC2660	*pykA*	pyruvate carboxylase, PYKA	No	6.4.1.1	PYR + ATP + HCO3 -> ADP + Pi + OAA		
CAC2680	*pgi*	glucose-6-phosphate isomerase	No	5.3.1.9	G6P <-> F6P	bDG6P <-> F6P	G6P <-> bDG6P
CAC2684		sugar kinase, ribokinase family	No	2.7.1.45	2DDGLCN + ATP -> 2DDG6P + ADP		
CAC2685		trehalose/maltose hydrolase (phosphorylase)	No	2.4.1.8	MALT + Pi -> bDGLC + bDG1P		
CAC2700	*guaA*	GMP synthase	No	6.3.5.2	XMP + LGLN + ATP -> GMP + PPi + LGLU + AMP	XMP + NH3 + ATP -> GMP + PPi + AMP	
CAC2701	*guaB*	IMP dehydrogenase	No	1.1.1.205	IMP + NAD -> XMP + NADH		
CAC2708	*hbd*	3-hydroxybutyryl-CoA dehydrogenase	No	1.1.1.157	ACTACCOA + NADH -> 3HBCOA + NAD		
CAC2709	*etfA*	electron transfer flavoprotein alpha-subunit	Yes		CRTCOA + 2 NADH + Fd(Ox) -> BUCOA + 2 NAD + Fd(Red)		

CAC2710	*etfB*	electron transfer flavoprotein beta-subunit	Yes		CRTCOA + 2 NADH + Fd(Ox) -> BUCOA + 2 NAD + Fd(Red)		
CAC2711	*bcd*	butyryl-CoA dehydrogenase	Yes	1.3.99.2	CRTCOA + 2 NADH + Fd(Ox) -> BUCOA + 2 NAD + Fd(Red)		
CAC2712	*crt*	3-hydroxybutyryl-CoA dehydratase	No	4.2.1.55	3HBCOA -> CRTCOA		
CAC2723		deacethylase/dipeptidase/desuccinylase family of Zn-dependent hydrolases	No	3.5.1.18	SL26DA -> SUCC + 26DAP-LL		
CAC2727		putative histidinol-phosphatase	No	3.1.3.15	HISP -> HISTD + Pi		
CAC2783	*cysD*	O-acetylhomoserine (thiol)-lyase	No	2.5.1.49	ACHMS + S -> LHCYS + AC		
CAC2819	*murE*	UDP-N-acetylmuramyl tripeptide synthase, MURE	No	6.3.2.13	1.064 UAMR + 1.064 UACGAM + 1.106 LALA + 1.106 LGLU + 1.106 DALADALA + 1.106 26DAP-M + 4.425 ATP -> PEPTIDO + 1.106 DALA + 1.106 UDP + 1.106 UMP + 4.425 ADP + 4.425 Pi		
CAC2830		acylphosphatases, ACYP	No	3.6.1.7	13DPG -> 3PG + Pi	ACTP -> AC + Pi	
CAC2832		PLP-dependent aminotransferase	No	2.6.1.1	AKG + LASP <-> OAA + LGLU	PHPYR + LGLU <-> LPHE + AKG	34HPP + LGLU <-> LTYR + AKG
CAC2834		glycerate kinase	No	2.7.1.31	ATP + GLYCAC -> ADP + 3PG		
CAC2844	*galT*	galactose-1-phosphate uridylyltransferase	No	2.7.7.10	UDPGLC + GAL1P <-> G1P + UDPGAL		
CAC2856	*metK*	S-adenosylmethionine synthetase	No	2.5.1.6	ATP + LMET -> AMET + Pi + PPi		

CAC2862	*murA*	UDP-N-acetylglucosamine 1-carboxyvinyltransferase	No	2.5.1.7	UACGAM + PEP <-> UACCG + Pi	
CAC2873		acetyl-CoA acetyltransferase	No	2.3.1.9	2 ACCOA -> ACTACCOA + COA	
CAC2876		deoxycytidylate deaminase	No	3.5.4.12	dCMP -> dUMP + NH3	
CAC2880		ribose 5-phosphate isomerase, RpiB	No	5.3.1.6	R5P <-> DRU5P	
CAC2891		fusion of alpha-glucosidase (family 31 glycosyl hydrolase) and	No	3.2.1.20	MALT -> 2 GLC	
CAC2892	*ctrA*	CTP synthase (UTP-ammonia lyase)	No	6.3.4.2	UTP + NH3 + ATP -> CTP + ADP + Pi	UTP + LGLN + ATP -> CTP + LGLU + ADP + Pi
CAC2895	*ddlA*	D-alanine-D-alanine ligase	No	6.3.2.4	2 DALA + ATP -> DALADALA + ADP + Pi	
CAC2902	*ipk*	4-diphosphocytidyl-2-C-methyl-D-erythritol kinase	No	2.7.1.148	CDPMERYTH + ATP -> CDPMERY2P + ADP	
CAC2914	*panB*	ketopantoate hydroxymethyltransferase	No	2.1.2.11	3MOB + MLTHF -> THF + 2DHP	
CAC2915	*panC*	pantoate--beta-alanine ligase	No	6.3.2.1	ATP + PANT + bALA -> AMP + PPi + PNTO	
CAC2916	*panD*	aspartate 1-decarboxylase	No	4.1.1.11	LASP -> bALA + CO2	
CAC2918	*pmi*	mannose-6 phospate isomerase	No	5.3.1.8	MAN6P <-> F6P	
CAC2926	*sul*	dihydropteroate synthase	No	2.5.1.15	ADHHP + PABA -> PPi + DHPT	

CAC2927	*folA, folK*	dihydroneopterin aldolase fused to 7,8-dihydro-6-hydroxymethylpterin-pyrophosphokinase	No	2.7.6.3/4.1.2.25	DHNP -> GLYCALD + AHHMDHP	ATP + AHHMDHP -> AMP + ADHHP
CAC2937		ketopantoate reductase PanE/ApbA	No	1.1.1.169	2DHP + NADPH -> PANT + NADP	
CAC2942		uncharacterized conserved protein fron YGAG family, predicted metal-dependent enzyme	No	4.4.1.21	RHCYS -> LHCYS + DRIB	
CAC2945		3-phosphoserine aminotransferase (Possible phosphoglycerate dehydrogenase)	No	2.6.1.52	3PHP + LGLU -> LPSER + AKG	
CAC2951	*lacC*	tagatose-6-phosphate kinase	No	2.7.1.144	TAG6P + ATP <-> TAGDP + ADP	
CAC2953	*lacB*	galactose-6-phosphate isomerase	No	5.3.1.26	GAL6P <-> TAG6P	
CAC2954	*lacA*	galactose-6-phosphate isomerase	No	5.3.1.26	GAL6P <-> TAG6P	
CAC2956		PTS system, galactitol-specific IIC component	Yes	2.7.1.69	GLCTT(Ext) + PEP -> GLCTT1P + PYR	
CAC2957		PTS system, galactitol-specific IIB component	Yes	2.7.1.69	GLCTT(Ext) + PEP -> GLCTT1P + PYR	
CAC2958		PTS system, galactitol-specific IIA component, putative	Yes	2.7.1.69	GLCTT(Ext) + PEP -> GLCTT1P + PYR	
CAC2959	*galK*	galactokinase	No	2.7.1.6	GAL + ATP -> GAL1P + ADP	
CAC2960	*galE*	UDP-galactose 4-epimerase	No	5.1.3.2	UDPGAL <-> UDPGLC	TDPGLC <-> TDPGAL
CAC2963	*lacG*	6-phospho-beta-D-galactosidase	No	3.2.1.85	LCTS6P <-> GLC + GAL6P	

CAC2964	*lacE*	PTS system lactose-specific enzyme IIBC	Yes	2.7.1.69	LCTS(Ext) + PEP -> LCTS6P + PYR		
CAC2965	*lacF*	PTS system lactose-specific enzyme IIA	Yes	2.7.1.69	LCTS(Ext) + PEP -> LCTS6P + PYR		
CAC2967		alpha-acetolactate decarboxylase	No	4.1.1.5	ACLAC -> ACETOIN + CO2		
CAC2973	*kdgA*	2-keto-3-deoxy-6-phosphogluconate aldolase, eda/kdgA	No	4.1.2.1	2DDG6P <-> GA3P + PYR		
CAC2995		PTS system (Glucose-specific) component IIA	YES	2.7.1.69	GLC(Ext) + PEP -> G6P + PYR		
CAC3003	*thyA*	thymidylate synthase	No	2.1.1.45	dUMP + MLTHF -> dTMP + DHF		
CAC3004	*folA*	dihydrofolate reductase	No	1.5.1.3	DHF + NADP <-> FOL + NADPH	THF + NADP <-> DHF + NADPH	
CAC3005	*add*	adenosine deaminase	No	3.5.4.4	dADN -> dINS + NH3	ADN -> INS + NH3	
CAC3020	*argJ*	amino-acid N-acetyltransferase / glutamate N-acetyltransferase	No	2.3.1.1/2.3.1.35	LGLU + ACCOA -> ACGLU + COA		
CAC3031	*hisC*	histidinol-phosphate aminotransferase	No	2.6.1.9	IMACP + LGLU <-> HISP + AKG	PHPYR + LGLU <-> LPHE + AKG	34HPP + LGLU <-> LTYR + AKG
CAC3075	*buk*	butyrate kinase, BUK	No	2.7.2.7	BUP + ADP -> BU + ATP		
CAC3076	*ptb*	phosphate butyryltransferase	No	2.3.1.19	BUCOA + Pi -> BUP + COA		
CAC3087		phosphoenolpyruvate-protein kinase (PTS system enzyme I)	Yes	2.7.3.9	All PTS reactions		

CAC3090		fumarate hydratase, subunit B (C-terminal domain of FumA E.coli)	Yes	4.2.1.2	MAL <-> FUM		
CAC3091		fumarate hydratase, subunit A (N-terminal domain of FumA E.coli)	Yes	4.2.1.2	MAL <-> FUM		
CAC3092	*231*	amidase, germination specific (cwlC/cwlD B.subtilis ortholog)	No	3.5.1.28	1.064 UAMR + 1.064 UACGAM + 1.106 LALA + 1.106 LGLU + 1.106 DALADALA + 1.106 26DAP-M + 4.425 ATP -		
CAC3112	*adk*	adenylate kinase	No	2.7.4.3	AMP + ATP <-> 2 ADP	dADP + ADP <-> dAMP + ATP	
CAC3157	*trpA*	tryptophan synthase alpha chain	Yes	4.2.1.20	3IG3P -> INDOLE + GA3P	LSER + INDOLE -> LTRP	
CAC3158	*trpB*	tryptophan synthase beta chain	Yes	4.2.1.20	3IG3P -> INDOLE + GA3P	LSER + INDOLE -> LTRP	
CAC3159	*trpF*	phosphoribosylanthranilate isomerase	No	5.3.1.24	PRAN <-> 2CPR5P		
CAC3160	*trpC*	indole-3-glycerol phosphate synthase	No	4.1.1.48	2CPR5P -> 3IG3P + CO2		
CAC3161	*trpD*	anthranilate phosphoribosyltransferase	No	2.4.2.18	ANTH + PRPP -> PRAN + PPi		
CAC3162	*pabA*	putative anthranilate synthase component II	Yes	4.1.3.27	CHOR + LGLN -> ANTH + PYR + LGLU		
CAC3163	*parB*	para-aminobenzoate synthase component I	Yes	4.1.3.27	CHOR + LGLN -> ANTH + PYR + LGLU		
CAC3169	*ilvB*	acetolactate synthase large subunit	Yes	2.2.1.6	THMPP + PYR -> HETHMPP + CO2	HETHMPP + PYR -> ACLAC + THMPP	2OBUT + HETHMPP -> 2AHBUT + THMPP
CAC3170	*ilvD*	dihydroxyacid dehydratase	No	4.2.1.9	23DHMP -> 3MOP	23DHMB -> 3MOB	

CAC3171	*leuB*	isopropylmalate dehydrogenase	No	1.1.1.85	3IPPMAL + NAD <-> 2IPPOSUCC + NADH			
CAC3172	*leuD*	3-isopropylmalate dehydratase, small subunit	Yes	4.2.1.33	2IPPMAL <-> 2IPPM	2IPPM <-> 3IPPMAL		
CAC3173	*leuC*	3-isopropylmalate dehydratase, large subunit	Yes	4.2.1.33	2IPPMAL <-> 2IPPM	2IPPM <-> 3IPPMAL		
CAC3174	*leuA*	2-isopropylmalate synthase	No	2.3.3.13	ACCOA + 3MOB -> 2IPPMAL + COA			
CAC3176	*ilvN*	acetolactate synthase small subunit	Yes	2.2.1.6	THMPP + PYR -> HETHMPP + CO2	HETHMPP + PYR -> ACLAC + THMPP	2OBUT + HETHMPP -> 2AHBUT + THMPP	
CAC3184	*ispD*	4-diphosphocytidyl-2-methylerithritol synthase (sugar nucleotide phosphorylase family)	No	2.7.7.60	MERYTH4P + CTP -> CDPMERYTH + PPi			
CAC3194	*murD*	UDP-N-acetylmuramoylalanine D-glutamate ligase	No	6.3.2.9	1.064 UAMR + 1.064 UACGAM + 1.106 LALA + 1.106 LGLU + 1.106 DALADALA + 1.106 26DAP-M + 4.425 ATP -> PEPTIDO + 1.106 DALA + 1.106 UDP + 1.106 UMP + 4.425 ADP + 4.425 Pi			
CAC3200		predicted transcriptional regulator, homolog of Bvg accessory factor	No	2.7.1.33	ATP + PNTO -> ADP + 4PPAN	ATP + 4PCYS -> ADP + 4PPCYS	ATP + PAN -> ADP + PAN4P	
CAC3201		formate--tetrahydrofolate ligase	No	6.3.4.3	THF + FOR + ATP -> ADP + Pi + 10FTHF			
CAC3203	*hprT*	hypoxanthine-guanine phosphoribosyltransferase	No	2.4.2.8	AMP + PPi <-> ADE + PRPP	HXAN + PRPP <-> IMP + PPi	GMP + PPi <-> GUA + PRPP	XAN + PRPP <-> XMP + PPi
CAC3221	*prs*	phosphoribosylpyrophosphate synthetase	No	2.7.6.1	R5P + ATP -> PRPP + AMP			
CAC3222	*gcaD*	glucosamine-1-phosphate N-acetyltransferase /		2.3.1.157/2.7.7.23	GAM1P + ACCOA -> ACGAM1P + COA	ACGAM1P + UTP <-> UACGAM + PPi		

CAC3225	*murC*	UDP-N-acetylmuramate-alanine ligase	No	6.3.2.8	1.064 UAMR + 1.064 UACGAM + 1.106 LALA + 1.106 LGLU + 1.106 DALADALA + 1.106 26DAP-M + 4.425 ATP - [illegible]			
CAC3250		possible glutamate racemase	No	5.1.1.3	LGLU <-> DGLU			
CAC3252	*proC*	pyrroline-5-carboxylate reductase	No	1.5.1.2	1PYR5C + NADPH <-> LPRO + NADP			
CAC3253	*proB*	glutamate 5-kinase	No	2.7.2.11	LGLU + ATP -> GLU5P + ADP			
CAC3254	*proA*	gamma-glutamyl phosphate reductase	No	1.2.1.41	GLU5P + NADPH <-> GLU5SA + NADP + Pi			
CAC3276	*nrdB*	ribonucleotide reductase beta subunit	Yes	1.17.4.1	ADP + TRD(Red) -> dADP + TRD(Ox)	GDP + TRD(Red) -> dGDP + TRD(Ox)	CDP + TRD(Red) -> dCDP + TRD(Ox)	UDP + TRD(Red) -> dUDP + TRD(Ox)
CAC3277	*nrdA*	ribonucleotide reductase alpha subunit	Yes	1.17.4.1	ADP + TRD(Red) -> dADP + TRD(Ox)	GDP + TRD(Red) -> dGDP + TRD(Ox)	CDP + TRD(Red) -> dCDP + TRD(Ox)	UDP + TRD(Red) -> dUDP + TRD(Ox)
CAC3298	*bdhB*	NADH-dependent butanol dehydrogenase B (BDH II)	No	1.1.1.-	BUAL + NADH <-> BUOH + NAD	BUAL + NADPH <-> BUOH + NADP		
CAC3299	*bdhA*	NADH-dependent butanol dehydrogenase A (BDH I)	No	1.1.1.-	BUAL + NADH <-> BUOH + NAD	BUAL + NADPH <-> BUOH + NADP		
CAC3316		possible cardiolipin synthase (phospholipase D family)	No	2.7.8.-	2 PG -> CDL + GLYC	PG + CDP-DAG -> CDL + CMP		
CAC3331		alanine racemase	No	5.1.1.1	LALA <-> DALA			
CAC3348	*mmuM*	possible homocysteine S-methyltransferase	No	2.1.1.10	LHMS + AMET -> LMET + AHCYS			
CAC3375		alcohol dehydrogenase	No	1.1.1.1	ACAL + NADH <-> ETOH + NAD	GLYC + NAD <-> GLYALD + NADH	XOL + NAD <-> DXYLU + NADH	

CAC3392		NADH-dependent butanol dehydrogenase	No	1.1.-.-	HIPCOA + NAD -> IPCHCCOA + NADH	MTNOL + O2 + NAD -> MTNAL + NADH	HDMHCOA + NAD -> DMMOHCOA + NADH		
CAC3420		low specificity L-threonine aldolase	No	4.1.2.5	LTHR <-> GLY + ACAL				
CAC3425	*glvC*	PTS system, (possibly glucose-specific) IIBC component	YES	2.7.1.69	GLC(Ext) + PEP -> G6P + PYR				
CAC3426	*glvG*	6-phospho-alpha-glucosidase	No	3.2.1.86	ARBT6P -> BZDO + bDG6P				
CAC3427		PTS system, (possibly glucose-specific) IIA component	YES	2.7.1.69	GLC(Ext) + PEP -> G6P + PYR				
CAC3462	*fabG*	3-oxoacyl-acyl carrier protein reductase	No	1.1.1.100	ACACP + 6 MALACP + 12 NADPH -> 12 NADP + C140-ACP + 6 CO2 + 6 ACP	ACACP + 7 MALACP + 14 NADPH -> 14 NADP + C160-ACP + 7 CO2 + 7 ACP	ACACP + 7 MALACP + 13 NADPH -> 13 NADP + C161-ACP + 7 CO2 + 7 ACP	ACACP + 8 MALACP + 16 NADPH -> 16 NADP + C180-ACP + 8 CO2 + 8 ACP	ACACP + 8 MALACP + 15 NADPH -> 15 NADP + C181-ACP + 8 CO2 + 8 ACP
CAC3471		GMP reductase	No	1.7.1.7	GMP + NADPH -> IMP + NH3 + NADP				
CAC3539	*murA*	UDP-N-acetylglucosamine enolpyruvyl transferase	No	2.5.1.7	UACGAM + PEP <-> UACCG + Pi				
CAC3552		lactate dehydrogenase	No	1.1.1.27	PYR + 2 NADH <-> LAC + 2 NAD				
CAC3568	*accA*	acetyl-CoA carboxylase alpha subunit	Yes	6.4.1.2	ACCOA + ATP + HCO3 <-> MALCOA + ADP + Pi				
CAC3569	*accD*	acetyl-CoA carboxylase beta subunit	Yes	6.4.1.2	ACCOA + ATP + HCO3 <-> MALCOA + ADP + Pi				
CAC3570	*accC*	biotin carboxylase	Yes	6.3.4.14/6.4.1.2	ACCOA + ATP + HCO3 <-> MALCOA + ADP + Pi				
CAC3571	*fabZ*	hydroxymyristoyl-(acyl carrier protein) dehydratase	No	4.2.1.60	ACACP + 6 MALACP + 12 NADPH -> 12 NADP + C140-ACP + 6 CO2 + 6 ACP	ACACP + 7 MALACP + 14 NADPH -> 14 NADP + C160-ACP + 7 CO2 + 7 ACP	ACACP + 7 MALACP + 13 NADPH -> 13 NADP + C161-ACP + 7 CO2 + 7 ACP	ACACP + 8 MALACP + 16 NADPH -> 16 NADP + C180-ACP + 8 CO2 + 8 ACP	ACACP + 8 MALACP + 15 NADPH -> 15 NADP + C181-ACP + 8 CO2 + 8 ACP

CAC3572	*accB*	biotin carboxyl carrier protein of acetyl-CoA carboxylase	Yes		ACCOA + ATP + HCO3 <-> MALCOA + ADP + Pi					
CAC3573	*fabF*	3-oxoacyl-(acyl-carrier-protein) synthase I	No	2.3.1.179	ACCOA + ACP <-> ACACP + COA	ACACP + 6 MALACP + 12 NADPH -> 12 NADP + C140-ACP + 6 CO2 + 6 ACP	ACACP + 7 MALACP + 14 NADPH -> 14 NADP + C160-ACP + 7 CO2 + 7 ACP	ACACP + 7 MALACP + 13 NADPH -> 13 NADP + C161-ACP + 7 CO2 + 7 ACP	ACACP + 8 MALACP + 16 NADPH -> 16 NADP + C180-ACP + 8 CO2 + 8 ACP	ACACP + 8 MALACP + 15 NADPH -> 15 NADP + C181-ACP + 8 CO2 + 8 ACP
CAC3574	*fabG*	3-ketoacyl-acyl carrier protein reductase	No	1.1.1.100	ACCOA + ACP <-> ACACP + COA	ACACP + 6 MALACP + 12 NADPH -> 12 NADP + C140-ACP + 6 CO2 + 6 ACP	ACACP + 7 MALACP + 14 NADPH -> 14 NADP + C160-ACP + 7 CO2 + 7 ACP	ACACP + 7 MALACP + 13 NADPH -> 13 NADP + C161-ACP + 7 CO2 + 7 ACP	ACACP + 8 MALACP + 16 NADPH -> 16 NADP + C180-ACP + 8 CO2 + 8 ACP	
CAC3575	*fabD*	malonyl CoA-acyl carrier protein transacylase	No	2.3.1.39	MALCOA + ACP <-> MALACP + COA					
CAC3576	*fabK*	trans-2-enoyl-ACP reductase II	No	1.3.1.9	ACACP + 6 MALACP + 12 NADPH -> 12 NADP + C140-ACP + 6 CO2 + 6 ACP	ACACP + 7 MALACP + 14 NADPH -> 14 NADP + C160-ACP + 7 CO2 + 7 ACP	ACACP + 7 MALACP + 13 NADPH -> 13 NADP + C161-ACP + 7 CO2 + 7 ACP	ACACP + 8 MALACP + 16 NADPH -> 16 NADP + C180-ACP + 8 CO2 + 8 ACP	ACACP + 8 MALACP + 15 NADPH -> 15 NADP + C181-ACP + 8 CO2 + 8 ACP	
CAC3578	*fabH*	3-oxoacyl-[acyl-carrier-protein] synthase III	No	2.3.1.180	ACCOA + ACP <-> ACACP + COA	ACACP + 6 MALACP + 12 NADPH -> 12 NADP + C140-ACP + 6 CO2 + 6 ACP	ACACP + 7 MALACP + 14 NADPH -> 14 NADP + C160-ACP + 7 CO2 + 7 ACP	ACACP + 7 MALACP + 13 NADPH -> 13 NADP + C161-ACP + 7 CO2 + 7 ACP	ACACP + 8 MALACP + 16 NADPH -> 16 NADP + C180-ACP + 8 CO2 + 8 ACP	ACACP + 8 MALACP + 15 NADPH -> 15 NADP + C181-ACP + 8 CO2 + 8 ACP
CAC3593	*purA*	adenylosuccinate synthase	No	6.3.4.4	IMP + LASP + GTP -> DCAMP + GDP + Pi					
CAC3596	*pgsA*	phosphatidylglycerophosphate synthase	No	2.7.8.5	CDP-DAG + GLYC3P -> CMP + PGP					
CAC3600	*dapA*	dihydrodipicolinate synthase	No	4.2.1.52	ASPSA + PYR -> 23DHDP					
CAC3604	*ilvD*	dihydroxy-acid dehydratase	No	4.2.1.9	23DHMP -> 3MOP	23DHMB -> 3MOB				
CAC3626	*mtrA*	GTP cyclohydrolase I	No	3.5.4.16	GTP -> FAPTP	FAPTP -> DAPTP + FOR	DAPTP -> DATPTHOPAOP	DATPTHOPAOP -> AHETHPDHPTP		
CAC3652	*alsS*	acetolactate synthase large subunit	Yes	2.2.1.6	THMPP + PYR -> HETHMPP + CO2	HETHMPP + PYR -> ACLAC + THMPP	2OBUT + HETHMPP -> 2AHBUT + THMPP			

CA_P0010	*bglA*	beta-glucosidase	No	3.2.1.21	CLB(Ext) -> 2 bDGLC(Ext)					
CA_P0025	*pdc*	pyruvate decarboxylase	No	4.1.1.1	THMPP + PYR -> HETHMPP + CO2	HETHMPP -> ACAL + THMPP				
CA_P0035	*adhE*	alcohol dehydrogenase / acetaldehyde dehydrogenase	No	1.1.1.1/1.2.1.10	ACAL + NADH <-> ETOH + NAD	GLYC + NAD <-> GLYALD + NADH	ACCOA + NADH <-> ACAL + COA + NAD	BUCOA + NADH <-> BUAL + COA + NAD	BUAL + NADPH <-> BUOH + NADP	BUAL + NADH <-> BUOH + NAD
CA_P0064	*alf*	fructose-bisphosphate aldolase class I	No	4.1.2.13	FDP -> DHAP + GA3P	F1P -> DHAP + GLYALD				
CA_P0066	*ptnA*	mannose-specific phosphotransferase system component IIAB	No	2.7.1.69	MAN(Ext) + PEP -> MAN6P + PYR					
CA_P0067	*many, levF*	mannose/fructose-specific phosphotransferase system component IIC	No	2.7.1.69	MAN(Ext) + PEP -> MAN6P + PYR					
CA_P0068	*ptnD*	mannose-specific phosphotransferase system component IID	No	2.7.1.69	MAN(Ext) + PEP -> MAN6P + PYR					
CA_P0078	*thiL*	acetyl coenzyme A acetyltransferase (thiolase)	No	2.3.1.9	2 ACCOA -> ACTACCOA + COA					
CA_P0088	*abf*	3-oxoacyl-acyl-carrier protein synthase	No	2.3.1.41	ACCOA + ACP <-> ACACP + COA					
CA_P0106	*dxs*	1-deoxyxylulose-5-phosphate synthase, dehydrogenase	No	2.2.1.7	PYR + GA3P -> dXYLU5P + CO2					
CA_P0122		dTDP-4-keto-L-rhamnose reductase	No	1.1.1.133	GDPoRHAM + NADPH -> GDPRHAM + NADP	TDPoRHAM + NADPH -> TDPRHAM + NADP				
CA_P0162	*adhE1*	alcohol dehydrogenase / acetaldehyde dehydrogenase	No	1.1.1.1/1.2.1.10	ACAL + NADH <-> ETOH + NAD	GLYC + NAD <-> GLYALD + NADH	ACCOA + NADH <-> ACAL + COA + NAD	BUCOA + NADH <-> BUAL + COA + NAD	BUAL + NADPH <-> BUOH + NADP	BUAL + NADH <-> BUOH + NAD
CA_P0163	*ctfA*	butyrate-acetoacetate CoA-transferase subunit A	Yes	2.8.3.9	AC + ACTACCOA -> ACCOA + ACTAC	BU + ACTACCOA -> BUCOA + ACTAC	SUCC + ACTACCOA -> ACTAC + SUCCOA			

CA_P0164	*ctfB*	butyrate-acetoacetate CoA-transferase subunit B	Yes	2.8.3.9	AC + ACTACCOA -> ACCOA + ACTAC	BU + ACTACCOA -> BUCOA + ACTAC	SUCC + ACTACCOA -> ACTAC + SUCCOA
CA_P0165	*adc*	acetoacetate decarboxylase	No	4.1.1.4	ACTAC -> ACETONE + CO2		

Incomplete GPR relationships
FUM + Fd(Red) <-> SUCC + Fd(Ox)
DRIB + ATP -> R5P + ADP
G1P <-> G6P
PAPS + TRD(Red) -> PAP + TRD(Ox) + SO3
PAP -> AMP + Pi
SO3 + 3 NADPH -> S + 3 NADP
LASP -> LALA + CO2
2IPSUCC -> 4MOP + CO2
GLU5SA <-> 1PY (spontaneous)
UDP + ATP <-> UTP + ADP
CTP + ADP <-> CDP + ATP
dCTP + ADP <-> dCDP + ATP
dUTP + ADP <-> dUDP + ATP
dUDP + ADP <-> dUMP + ATP
dTMP + ATP <-> dTDP + ADP
dTDP + ATP <-> dTTP + ADP
GLYC3P + 0.073 C140-ACP + 0.521 C160-ACP + 0.065 C161-ACP + 0.036 C180-ACP + 0.102 C181-ACP + 0.022 C17CYC-ACP + 0.181 C19CYC-ACP -> 1-Acyl-GLYC3P + ACP
PGP -> PG + Pi
12 PG -> 12 1,2-Diacyl-GLYC + POLYGP
C161-ACP + AMET -> C17CYC-ACP + AHCYS
C181-ACP + AMET -> C19CYC-ACP + AHCYS
5APRU -> 4R5AU + Pi
RIBFLA -> DMBZID

Metabolite abbreviation

Abbreviation	Full name
10FTHF	10-Formyltetrahydrofolate
12DAG	1,2-diacylglycerol
13DPG	1,3-Bisphospho-D-glycerate
1APROH	(R)-1-Aminopropan-2-ol
1MAG3P	1-acylglycerol-3-phosphate
1PYR5C	1-Pyrroline-5-carboxylate
23DHDP	L-2,3-Dihydrodipicolinate
23DHMB	(R)-2,3-Dihydroxy-3-methylbutanoate
23DHMP	(R)-2,3-Dihydroxy-3-methylpentanoate
25DRAPP	2,5-Diamino-6-(5'-phosphoribosylamino)-4-pyrimidineone
26DAP-LL	LL-2,6-Diaminoheptanedioate
26DAP-M	meso-2,6-Diaminoheptanedioate
2AHBUT	(S)-2-Aceto-2-hydroxybutanoate
2CPR5P	1-(2-Carboxyphenylamino)-1'-deoxy-D-ribulose 5'-phosphate
2DDA7P	2-Dehydro-3-deoxy-D-arabino-heptonate 7-phosphate
2DDG6P	2-Dehydro-3-deoxy-6-phospho-D-gluconate
2DDGLCN	2-Dehydro-3-deoxy-D-gluconate
2DHP	2-Dehydropantoate
2DR1P	2-Deoxy-D-ribose 1-phosphate
2HBUT	2-Hydroxybutyrate
2IPPM	2-Isopropylmaleate
2IPPMAL	(2S)-2-Isopropylmalate
2IPSUCC	(2S)-2-Isopropyl-3-oxosuccinate
2OBUT	2-Oxobutanoate
2PG	2-Phospho-D-glycerate
34HPP	3-(4-Hydroxyphenyl)pyruvate
3DHQ	3-Dehydroquinate
3DHSK	3-Dehydroshikimate
3H3MOB	3-Hydroxy-3-methyl-2-oxobutanoate
3H3MOP	(R)-3-Hydroxy-3-methyl-2-oxopentanoate
3HBCOA	3-Hydroxybutanoyl-CoA
3IG3P	Indoleglycerol phosphate

3IPPMAL	(2R,3S)-3-Isopropylmalate
3MOB	3-Methyl-2-oxobutanoate
3MOP	3-Methyl-2-oxopentanoate
3PG	3-Phospho-D-glycerate
3PHP	3-Phosphonooxypyruvate
3PSME	5-O-(1-Carboxyvinyl)-3-phosphoshikimate
4ABUT	4-Aminobutyrate
4H2KPM	4-Hydroxy-2-ketopimelate
4MOP	4-Methyl-2-oxopentanoate
4PASP	4-Phospho-L-aspartate
4PCYS	N-((R)-Pantothenoyl)-L-cysteine
4PPAN	D-4'-Phosphopantothenate
4PPCYS	(R)-4'-Phosphopantothenoyl-L-cysteine
4R5AU	4-(1-D-Ribitylamino)-5-aminouracil
5AOP	5-Aminolevulinate
5APRBU	5-Amino-6-(5'-phosphoribosylamino)uracil
5APRU	5-Amino-6-(5'-phosphoribitylamino)uracil
5FTHF	5-Formyltetrahydrofolate
5METRIB	5-Methylthio-D-ribose
5MTHF	5-methyltetrahydrofolate
5PRDMBZ	N1-(5-Phospho-alpha-D-ribosyl)-5,6-dimethylbenzimidazole
AC	Acetate
AC(Ext)	Acetate(Extracellular)
ACACP	Acetyl-[acyl-carrier protein]
ACAL	Acetaldehyde
ACBA	Adenosyl cobinamide
ACBAP	Adenosyl cobinamide phosphate
ACBRNDA	Adenosyl cobyrinate a,c diamide
ACBRNHA	Adenosyl cobyrinate hexaamide
ACCOA	Acetyl-CoA
ACETOIN	Acetoin
ACETOIN(Ext)	Acetoin(Extracellular)
ACETONE	Acetone
ACETONE(Ext)	Acetone(Extracellular)
ACGAM(Ext)	N-Acetyl-D-glucosamine(Extracellular)
ACGAM1P	N-Acetyl-D-glucosamine 1-phosphate

ACGAM6P	N-Acetyl-D-glucosamine 6-phosphate
ACGLU	N-Acetyl-L-glutamate
ACGLU5P	N-Acetyl-L-glutamate 5-phosphate
ACGLU5SA	N-Acetyl-L-glutamate 5-semialdehyde
ACHMS	O-Acetyl-L-homoserine
ACLAC	2-Acetolactate
ACORN	N-Acetylornithine
ACP	Acyl-carrier protein
ACSER	O-Acetyl-L-serine
ACTAC	Acetoacetate
ACTACCOA	Acetoacetyl-CoA
ACTP	Acetyl phosphate
ADE	Adenine
ADHHP	Amino-7,8-dihydro-4-hydroxy-6-(diphosphooxymethyl)pteridine
ADN	Adenosine
ADP	Adenosine 5'-diphosphate
ADPGLC	ADP-glucose
AGDPCBA	Adenosine-GDP-cobinamide
AHCYS	S-Adenosyl-L-homocysteine
AHHMDHP	2-Amino-4-hydroxy-6-hydroxymethyl-7,8-dihydropteridine
AHTHDH	2-Amino-4-hydroxy-6-(erythro-1,2,3-trihydroxypropyl)dihydropteridine triphosphate
AICAR	1-(5'-Phosphoribosyl)-5-amino-4-imidazolecarboxamide
AIR	Aminoimidazole ribotide
AKG	2-Oxoglutarate
AMET	S-adenosyl-L-methionine
AMETA	S-Adenosylmethioninamine
AMP	Adenosine 5'-monophosphate
ANTH	Anthranilate
APROHP	D-1-Aminopropan-2-ol O-phosphate
APS	Adenylyl sulfate
ARBZL	N1-(alpha-D-ribosyl)-5,6-dimethylbenzimidazole
ARBZL5P	N1-(5-Phospho-alpha-D-ribosyl)-5,6-dimethylbenzimidazole
ARGSUC	N-(L-Arginino)succinate
ASPSA	L-Aspartate 4-semialdehyde
ATP	Adenosine 5'-triphosphate

bALA	beta-Alanine
bDG1P	beta-D-Glucose 1-phosphate
bDG6P	beta-D-Glucose 6-phosphate
bDGLC	beta-D-Glucose
bDGLC(Ext)	beta-D-Glucose(Extracellular)
BIOMASS	Biomass
BU	Butyrate
BU(Ext)	Butyrate(Extracellular)
BUAL	Butyraldehyde
BUCOA	Butyryl-CoA
BUOH	1-Butanol
BUOH(Ext)	1-Butanol(Extracellular)
BUP	Butyryl phosphate
C140-ACP	C14:0-[acyl-carrier protein]
C160-ACP	C16:0-[acyl-carrier protein]
C161-ACP	C16:1-[acyl-carrier protein]
C17CYC-ACP	C17:cyclic-[acyl-carrier protein]
C180-ACP	C18:0-[acyl-carrier protein]
C181-ACP	C18:1-[acyl-carrier protein]
C19CYC-ACP	C19:cyclic-[acyl-carrier protein]
CACO	Cobamide coenzyme
CARBO	Carbohydrate
CBASP	N-Carbamoyl-L-aspartate
CBP	Carbamoyl phosphate
CBRN	Cobyrinate
CBRNDA	Cob(II)yrinate a,c diamide
CDHPRCR6	Cobalt-precorrin 6B
CDL	Cardiolipin
CDP	Cytidine 5'-diphosphate
CDP-DAG	CDP-Diacylglycerol
CDPMERY2P	2-Phospho-4-(cytidine 5'-diphospho)-2-C-methyl-D-erythritol
CDPMERYTH	4-(Cytidine 5'-diphospho)-2-C-methyl-D-erythritol
CDV	Cadverine
CHOR	Chorismate
CIT	Citrate
CLB(Ext)	Cellobiose(Extracellular)
CMP	Cytidine-5'-monophosphate
CO2	Carbon dioxide
CO2(Ext)	Carbon dioxide(Extracellular)
COA	Coenzyme A
COBALT	Cobalt ion
CORR	Corrinoid
CPPPG3	Coproporphyrinogen III
CPRCR2	Cobalt-precorrin 2
CPRCR3	Cobalt-precorrin 3
CPRCR4	Cobalt-precorrin 4

CPRCR5A	Cobalt-precorrin 5A
CPRCR5B	Cobalt-precorrin 5B
CPRCR6	Cobalt-precorrin 6
CPRCR7	Cobalt-precorrin 7
CPRCR8	Cobalt-precorrin 8
CRTCOA	Crotonoyl-CoA
CTP	Cytidine 5'-triphosphate
CYST	Cystathionine
dADN	Deoxyadenosine
dADP	2'-Deoxyadenosine 5'-diphosphate
DALA	D-Alanine
DALADALA	D-Alanyl-D-Alanine
dAMP	2'-Deoxyadenosine 5'-phosphate
DAPTP	2,5-Diaminopyrimidine nucleoside triphosphate
DATHAO	2,5-Diamino-6-(5'-triphosphoryl-3',4'-trihydroxy-2'-oxopentyl)-amino-4-oxopyrimidine
dATP	2'-Deoxyadenosine 5'-triphosphate
DB4P	3,4-Dihydroxy-2-butanone 4-phosphate
DCAMP	N6-(1,2-Dicarboxyethyl)-AMP
dCDP	2'-Deoxycytidine 5'-diphosphate
dCMP	2'-Deoxycytidine 5'-monophosphate
dCTP	2'-Deoxycytidine 5'-triphosphate
dGDP	2'-Deoxyguanosine 5'-diphosphate
DGLU	D-Glutamate
dGTP	2'-Deoxyguanosine 5'-triphosphate
DHAP	Dihydroxyacetone phosphate
DHF	Dihydrofolate
DHNP	2-Amino-4-hydroxy-6-(D-erythro-1,2,3-trihydroxypropyl)-7,8-dihydropteridine
DHNPP	Dihydroneopterin phosphate
DHOR-S	(S)-Dihydroorotate
DHPT	Dihydropteroate
dINS	Deoxyinosine
DMBZID	Dimethylbenzimidazole
DMLZ	6,7-Dimethyl-8-(1-D-ribityl)lumazine

DMMOHCOA	2,6-Dimethyl-5-methylene-3-oxo-heptanoyl-CoA
DMPP	Dimethylallyl diphosphate
DNA	DNA
DNAD	Deamino-NAD+
DPCOA	Dephospho-CoA
DPHE	D-Phenylalanine
DRIB	D-Ribose
DRU5P	D-Ribulose 5-phosphate
dTDP	Deoxythymidine 5'-diphosphate
dTMP	Deoxythymidine 5'-phosphate
dTTP	Deoxythymidine 5'-triphosphate
dUDP	2'-Deoxyuridine 5'-diphosphate
dUMP	2'-Deoxyuridine 5'-phosphate
dUTP	2'-Deoxycytidine 5'-triphosphate
DXU5P	D-Xylulose 5-phosphate
DXYL	D-Xylose
DXYL(Ext)	D-Xylose(Extracellular)
DXYLU	D-Xylulose
dXYLU5P	1-Deoxy-D-xylulose 5-phosphate
E4P	D-Erythrose 4-phosphate
EIG3P	D-erythro-1-(Imidazol-4-yl)glycerol 3-phosphate
ETOH	Ethanol
ETOH(Ext)	Ethanol(Extracellular)
F1P	D-Fructose 1-phosphate
F6P	beta-D-Fructose 6-phosphate
FAD	Flavin adenine dinucleotide
FAPTP	Formamidopyrimidine nucleoside triphosphate
Fd(Ox)	Oxidized ferredoxin
Fd(Red)	Reduced ferredoxin
FDP	beta-D-Fructose 1,6-bisphosphate
Fe2	Ferrous ion
FGAM	2-(Formamido)-N1-(5'-phosphoribosyl)acetamidine
FGAR	5'-Phosphoribosyl-N-formylglycinamide
FMN	Flavin mononucleotide
FOL	Folate
FORM	Formate
FORM(Ext)	Formate(Extracellular)
FPRICA	1-(5'-Phosphoribosyl)-5-formamido-4-imidazolecarboxamide

FRDP	trans,trans-Farnesyl diphosphate
FRU	D-Fructose
FRU(Ext)	D-Fructose(Extracellular)
FUM	Fumarate
G1P	alpha-D-Glucose 1-phosphate
G6P	alpha-D-Glucose 6-phosphate
GA3P	D-Glyceraldehyde 3-phosphate
GAL	D-Galactose
GAL(Ext)	D-Galactose(Extracellular)
GAL1P	alpha-D-Galactose 1-phosphate
GAL6P	D-Galactose 6-phosphate
GAM1P	D-Glucosamine 1-phosphate
GAM6P	D-Glucosamine 6-phosphate
GAR	5'-Phosphoribosylglycinamide
GDP	Guanosine 5'-diphosphate
GDPoRHAM	GDP-4-dehydro-6-deoxy-L-mannose
GDPRHAM	GDP-6-deoxy-L-mannose
GGRDP	Geranylgeranyl diphosphate
GLC	Alpha-D-glucose
GLC(Ext)	Alpha-D-glucose(Extracellular)
GLU1SA	L-Glutamate 1-semialdehyde
GLU5P	L-Glutamyl 5-phosphate
GLU5SA	L-Glutamate 5-semialdehyde
GLY	Glycine
GLY(Ext)	Glycine(Extracellular)
GLYALD	D-Glyceraldehyde
GLYC	Glycerol
GLYC(Ext)	Glycerol(Extracellular)
GLYC3P	sn-Glycerol 3-phosphate
GLYCAC	D-Glycerate
GLYCALD	Glycolaldehyde
GLYCALD(Ext)	Glycolaldehyde(Extracellular)
Glycogen	Glycogen
GMP	Guanosine 5'-phosphate
GRDP	Geranyl diphosphate
GTH(Ox)	Glutathione disulfide
GTH(Red)	Glutathione
GTP	Guanosine 5'-triphosphate
GUA	Guanine
H2	Hydrogen
H2(Ext)	Hydrogen(Extracellular)
H2O2	Hydrogen Peroxide
H2O2(Ext)	Hydrogen Peroxide(Extracellular)
HCO3	Bicarbonate

HDMHCOA	3-Hydroxy-2,6-dimethyl-5-methylene-heptanoyl-CoA
HETHMPP	2-(alpha-Hydroxyethyl)thiamine diphosphate
HGBRN	Hydrogenobyrinate
HIPCOA	2-Hydroxy-4-isopropenylcyclohexane-1-carboxyl-CoA
HISP	L-Histidinol phosphate
HISTD	L-Histidinol
HISTDAL	L-Histidinal
HMB4DP	1-Hydroxy-2-methyl-2-butenyl 4-diphosphate
HMBIL	Hydroxymethylbilane
HOR	Hordenine
HPYR	Hydroxypyruvate
HXAN	Hypoxanthine
ICIT	Isocitrate
IMACP	3-(Imidazol-4-yl)-2-oxopropyl phosphate
IMP	Inosine 5'-monophosphate
INDOLE	Indole
INS	Inosine
IPCHCCOA	4-Isopropenyl-2-oxy-cyclohexanecarboxyl-CoA
IPDP	Isopentenyl diphosphate
LAC	(S)-Lactate
LAC(Ext)	(S)-Lactate(Extracellular)
LALA	L-Alanine
LALA(Ext)	L-Alanine
LARAB	L-Arabinose
LARAB(Ext)	L-Arabinose(Extracellular)
LARG	L-Arginine
LARG(Ext)	L-Arginine(Extracellular)
LASN	L-Asparagine
LASN(Ext)	L-Asparagine(Extracellular)
LASP	L-Aspartate
LASP(Ext)	L-Aspartate(Extracellular)
LCITR	L-Citrulline
LCTS(Ext)	Lactose(Extracellular)
LCTS6P	Lactose 6-phosphate
LCYS	L-Cysteine
LCYS(Ext)	L-Cysteine(Extracellular)
LGLN	L-Glutamine
LGLN(Ext)	L-Glutamine(Extracellular)
LGLU	L-Glutamate
LGLU(Ext)	L-Glutamate(Extracellular)
LHCYS	L-Homocysteine
LHIS	L-Histidine

LHIS(Ext)	L-Histidine(Extracellular)
LHMS	L-Homoserine
LILE	L-Isoleucine
LILE(Ext)	L-Isoleucine(Extracellular)
LLEU	L-Leucine
LLEU(Ext)	L-Leucine(Extracellular)
LLYS	L-Lysine
LLYS(Ext)	L-Lysine(Extracellular)
LMET	L-Methionine
LMET(Ext)	L-Methionine(Extracellular)
LORN	L-Ornithine
LPHE	L-Phenylalanine
LPHE(Ext)	L-Phenylalanine(Extracellular)
LPRO	L-Proline
LPRO(Ext)	L-Proline(Extracellular)
LPSER	O-Phospho-L-serine
LRBL	L-Ribulose
LRU5P	L-Ribulose 5-phosphate
LSER	L-Serine
LSER(Ext)	L-Serine(Extracellular)
LTHR	L-Threonine
LTHR(Ext)	L-Threonin(Extracellular)
LTRP	L-Tryptophan
LTRP(Ext)	L-Tryptophan(Extracellular)
LTYR	L-Tyrosine
LTYR(Ext)	L-Tyrosine(Extracellular)
LVAL	L-Valine
LVAL(Ext)	L-Valine(Extracellular)
MAL	(S)-Malate
MAL(Ext)	(S)-Malate(Extracelllular)
MALACP	Malonyl-[acyl-carrier protein]
MALCOA	Malonyl-CoA
MALT	Maltose
MALT(Ext)	Maltose(Extracellular)
MALT6P	Maltose 6'-phosphate
MAN(Ext)	D-Mannose(Extracellular)
MAN6P	D-Mannose 6-phosphate
MECORR	Methylcorrinoid
MERYcDP	2-C-Methyl-D-erythritol 2,4-cyclodiphosphate
MERYTH4P	2-C-Methyl-D-erythritol 4-phosphate
METADN	5'-Methylthioadenosine
METHF	5,10-Methenyltetrahydrofolate
MLHIS	N(pi)-Methyl-L-histidine
MLTHF	5,10-Methylenetetrahydrofolate
MNL(Ext)	Mannitol
MNL1P	D-Mannitol 1-phosphate
MTNAL	Myrtenal

MTNOL	Myrtenol
MTYRAM	N-Methyltyramine
N2	Nitrogen
N2(Ext)	Nitrogen(Extracellular)
NA	Nicotinic acid
NA(Ext)	Nicotinic acid(Extracellular)
NAD	NAD+
NADH	NADH
NADP	NADP+
NADPH	NADPH
NAMN	Nicotinate D-ribonucleotide
NAMNs	Nicotinate D-ribonucleoside
NH3	Ammonium ion
NH3(Ext)	Ammonium ion(Extracellular)
NMN	Nicotinamide D-ribonucleotide
NO2	Nitrite
NO2(Ext)	Nitrite(Extracellular)
O2	Oxygen
OAA	Oxaloacetate
OROT	Orotate
OROT5P	Orotidine 5'-phosphate
PA	Phosphatidate
PABA	4-Aminobenzoate
PABA(Ext)	4-Aminobenzoate(Extracellular)
PAN	Pantetheine
PAN4P	Pantetheine 4'-phosphate
PANT	(R)-Pantoate
PAP	Adenosine 3',5'-bisphosphate
PAPS	3'-Phosphoadenylyl sulfate
PE	Phosphatidylethanolamine
PEP	Phosphoenolpyruvate
PEPTIDO	Peptidoglycan
PG	Phosphatidylglycerol
PGP	Phosphatidylglycerophosphate
PHOM	O-Phospho-L-homoserine
PHPYR	Phenylpyruvate
Pi	Inorganic phosphate
Pi(Ext)	Inorganic phosphate(Extracellular)
PLIPID	Phospholipid
PNTO	Pantothenate
POLYGP	Polyglycerol phosphate
PPBNG	Porphobilinogen
PPHN	Prephenate
PPi	Pyrophosphate
PPPG9	Protoporphyrinogen IX
PRAIC	1-(5-Phospho-D-ribosyl)-5-amino-4-imidazolecarboxylate
PRAM	5-Phosphoribosylamine

PRAN	N-(5-Phospho-D-ribosyl)anthranilate
PRBAMP	Phosphoribosyl-AMP
PRBATP	Phosphoribosyl-ATP
PRCR2	Precorrin 2
PRCR3B	Precorrin 3B
PRCR4	Precorrin 4
PRCR5	Precorrin 5
PRCR6A	Precorrin 6A
PRCR6B	Precorrin 6B
PRCR8	Precorrin 8
PRFP	5-(5-Phospho-D-ribosylaminoformimino)-1-(5-phosphoribosyl)-imidazole-4-carboxamide
PRLP	N-(5'-Phospho-D-1'-ribulosylformimino)-5-amino-1-(5''-phospho-D-ribosyl)-4-imidazolecarboxamide
PROCOA	Propionyl-CoA
ProDS	Protein disulfide
ProDTH	Protein dithiol
PROP	Propionate
PROP(Ext)	Propionate(Extracellular)
PROPP	Propionyl phosphate
PROTEIN	Protein
PRPP	5-Phospho-alpha-D-ribose 1-diphosphate
PS	Phosphatidylserine
PTRC	Putrecine
PYR	Pyruvate
PYR(Ext)	Pyruvate(Extracellular)
QULN	Pyridine-2,3-dicarboxylate
R1P	D-Ribose 1-phosphate
R5P	D-Ribose 5-phosphate
RHCYS	S-(5-deoxy-D-ribos-5-yl)-L-homocysteine
RIBFLA	Riboflavin
RNA	RNA
S	Sulfide
S7P	D-Sedoheptulose 7-phosphate
SAICAR	1-(5'-Phosphoribosyl)-5-amino-4-(N-succinocarboxamide)-imidazole
SHCL	Sirohydrochlorin
SHEME	Siroheme
SKM	Shikimate
SKM3P	Shikimate 3-phosphate

SL26DA	N-Succinyl-LL-2,6-diaminoheptanedioate
SL2A6O	N-Succinyl-2-L-amino-6-oxoheptanedioate
SO3	Sulfite
SO4	Sulfate
SO4(Ext)	Sulfate(Extracellular)
SPERMD	Spermidine
SUC6P	Sucrose 6-phosphate
SUCC	Succinate
SUCCOA	Succinyl-CoA
SUCCSA	Succinate semialdehyde
SUCHMS	O-Succinyl-L-homoserine
SUCR(Ext)	Sucrose(Extracellular)
TAG6P	D-Tagatose 6-phosphate
TAGDP	D-Tagatose 1,6-bisphosphate
TDPDHdGLC	dTDP-4-dehydro-6-deoxy-alpha-D-glucose
TDPGAL	dTDP-galactose
TDPGLC	dTDP-glucose
TDPoRHAM	dTDP-4-dehydro-6-deoxy-L-mannose
TDPRHAM	dTDP-6-deoxy-L-mannose
TEICH	Teichoic acid
THDP	2,3,4,5-Tetrahydrodipicolinate
THF	Tetrahydrofolate
THMPP	Thiamin pyrophosphate
TRACE	Trace components
TRD(Ox)	Oxidized thioredoxin
TRD(Red)	Reduced thioredoxin
UACCG	UDP-N-acetyl-3-(1-carboxyvinyl)-D-glucosamine
UACGAM	UDP-N-acetyl-D-glucosamine
UAMR	UDP-N-acetylmuramate
UDCPDP	Undecaprenyl diphosphate
UDP	Uridine 5'-diphosphate
UDPGAL	UDP-D-galactose
UDPGLC	UDP-D-glucose
UMP	Uridine 5'-monophosphate
UPPG3	Uroporphyrinogen III
UREA	Urea
UREA(Ext)	Urea(Ext)
UTP	Uridine 5'-triphosphate
XAN	Xanthine
XANT	Xanthosine
XMP	Xanthosine 5'-phosphate
XOL	Xylitol

Whole reaction set of *Cac* MBEL502

↔ Reversible reaction
→ Irreversible reaction

Reaction #	Name	Reaction
R001	GLCpts	GLC(Ext) + PEP → G6P + PYR
R002	FRUpts	FRU(Ext) + PEP → PYR + F1P
R003	MNLpts	MNL(Ext) + PEP → MNL1P + PYR
R004	MANpts	MAN(Ext) + PEP → MAN6P + PYR
R005	LACTpts	LCTS(Ext) + PEP → LCTS6P + PYR
R006	SUCRpts	SUCR(Ext) + PEP → SUC6P + PYR
R007	MALTpts	MALT(Ext) + PEP → MALT6P + PYR
R008	ACGAMpts	ACGAM(Ext) + PEP → ACGAM6P + PYR
R009	XYLt	DXYL(Ext) ↔ DXYL
R010	ARABt	LARAB(Ext) ↔ LARAB
R011	GALt	GAL(Ext) ↔ GAL
R012	NO2t	NO2(Ext) → NO2
R013	N2t	N2(Ext) → N2
R014	CO2t	CO2(Ext) ↔ CO2
R015	H2t	H2 → H2(Ext)
R016	NH3t	NH3(Ext) → NH3
R017	ETOHt	ETOH → ETOH(Ext)
R018	BUOHt	BUOH → BUOH(Ext)
R019	ACETONEt	ACETONE → ACETONE(Ext)
R020	ACETOINt	ACETOIN → ACETOIN(Ext)
R021	SULFATEt	SO4(Ext) + ATP → SO4 + ADP + Pi
R022	Pi_t	Pi(Ext) → Pi
R023	Pi_abc	Pi(Ext) + ATP → ADP + 2 Pi
R024	GLYt	GLY(Ext) ↔ GLY
R025	LALAt	LALA(Ext) ↔ LALA
R026	LVALt	LVAL(Ext) ↔ LVAL
R027	LLEUt	LLEU(Ext) ↔ LLEU
R028	LILEt	LILE(Ext) ↔ LILE
R029	LGLUt	LGLU(Ext) ↔ LGLU
R030	LGLNt	LGLN(Ext) ↔ LGLN
R031	LMETt	LMET(Ext) ↔ LMET
R032	LCYSt	LCYS(Ext) ↔ LCYS
R033	LASPt	LASP(Ext) ↔ LASP
R034	LASNt	LASN(Ext) ↔ LASN
R035	LPROt	LPRO(Ext) ↔ LPRO
R036	LTRPt	LTRP(Ext) ↔ LTRP
R037	LTYRt	LTYR(Ext) ↔ LTYR
R038	LHISt	LHIS(Ext) ↔ LHIS
R039	LPHEt	LPHE(Ext) ↔ LPHE
R040	LSERt	LSER(Ext) ↔ LSER
R041	LTHRt	LTHR(Ext) ↔ LTHR
R042	LLYSt	LLYS(Ext) ↔ LLYS

R043	LARGt	LARG(Ext) ↔ LARG
R044	LALAabc	LALA(Ext) + ATP → LALA + ADP + Pi
R045	LVALabc	LVAL(Ext) + ATP → LVAL + ADP + Pi
R046	LTHRabc	LTHR(Ext) + ATP → LTHR + ADP + Pi
R047	LCYSabc	LCYS(Ext) + ATP → LCYS + ADP + Pi
R048	LILEabc	LILE(Ext) + ATP → LILE + ADP + Pi
R049	LASNabc	LASN(Ext) + ATP → LASN + ADP + Pi
R050	LASPabc	LASP(Ext) + ATP → LASP + ADP + Pi
R051	LGLNabc	LGLN(Ext) + ATP → LGLN + ADP + Pi
R052	LGLUabc	LGLU(Ext) + ATP → LGLU + ADP + Pi
R053	LARGabc	LARG(Ext) + ATP → LARG + ADP + Pi
R054	LHISabc	LHIS(Ext) + ATP → LHIS + ADP + Pi
R055	LLYSabc	LLYS(Ext) + ATP → LLYS + ADP + Pi
R056	LPROabc	LPRO(Ext) + ATP → LPRO + ADP + Pi
R057	LMETabc	LMET(Ext) + ATP → LMET + ADP + Pi
R058	ACt	AC ↔ AC(Ext)
R059	BUt	BU ↔ BU(Ext)
R060	PROPt	PROP ↔ PROP(Ext)
R061	MALt	MAL ↔ MAL(Ext)
R062	UREAt	UREA → UREA(Ext)
R063	NAt	NA ↔ NA(Ext)
R064	LACt	LAC ↔ LAC(Ext)
R065	PYRt	PYR ↔ PYR(Ext)
R066	GLYCALDt	GLYCALD → GLYCALD(Ext)
R067	PABAt	PABA(Ext) → PABA
R068	GLYCt	[GLYC] ↔ [GLYC(Ext)]
R069	FORMt	FORM ↔ FORM(Ext)
R070	bDGLC	bDGLC(Ext) ↔ GLC(Ext)
R071	HCO3	CO2 ↔ HCO3
R072	H2O2t	H2O2 ↔ H2O2(Ext)
R073	PPi	PPi → 2 Pi
R074	ATP	ATP → ADP + Pi
R075	EMP1	[bDG6P] + [ADP] ↔ [ATP] + [bDGLC]
R076	EMP2	bDGLC ↔ GLC
R077	EMP3	GLC + ATP ↔ G6P + ADP
R078	EMP4	G6P ↔ F6P
R079	EMP5	bDG6P ↔ F6P
R080	EMP6	F6P + ATP → FDP + ADP
R081	EMP7	FDP → F6P + Pi
R082	EMP8	FDP → DHAP + GA3P
R083	EMP9	DHAP ↔ GA3P
R084	EMP10	GA3P + Pi + NAD ↔ 13DPG + NADH
R085	EMP11	13DPG + ADP ↔ 3PG + ATP
R086	EMP12	13DPG → 3PG + Pi
R087	EMP13	3PG ↔ 2PG
R088	EMP14	2PG ↔ PEP
R089	EMP15	PEP + ADP → PYR + ATP
R090	EMP16	PYR + NADH ↔ LAC + NAD
R091	EMP17	PYR + COA + Fd(Ox) → ACCOA + CO2 + Fd(Red)

R092	EMP18	Fd(Red) + NAD ↔ Fd(Ox) + NADH
R093	EMP19	Fd(Red) + NADP → Fd(Ox) + NADPH
R094	EMP20	Fd(Red) → Fd(Ox) + H_2
R095	PROPAN1	2HBUT + NAD ↔ 2OBUT + NADH
R096	PROPAN2	2OBUT + COA → PROCOA + FORM
R097	PROPAN3	PROCOA + Pi → PROPP + COA
R098	PROPAN4	PROPP + ADP → PROP + ATP
R099	BUTAN1	ACCOA + NADH ↔ ACAL + COA + NAD
R100	BUTAN2	ACAL + NADH ↔ ETOH + NAD
R101	BUTAN3	ACCOA + Pi ↔ ACTP + COA
R102	BUTAN4	ACTP + ADP ↔ AC + ATP
R103	BUTAN5	2 ACCOA → ACTACCOA + COA
R104	BUTAN6	ACTACCOA + NADH → 3HBCOA + NAD
R105	BUTAN7	3HBCOA → CRTCOA
R106	BUTAN8	CRTCOA + 2 NADH + Fd(Ox) → BUCOA + 2 NAD + Fd(Red)
R107	BUTAN9	BUCOA + Pi → BUP + COA
R108	BUTAN10	BUP + ADP → BU + ATP
R109	BUTAN11	BUCOA + NADH ↔ BUAL + COA + NAD
R110	BUTAN12	BUAL + NADH ↔ BUOH + NAD
R111	BUTAN13	ACTAC → ACETONE + CO_2
R112	BUTAN14	THMPP + PYR → HETHMPP + CO_2
R113	BUTAN15	HETHMPP + PYR → ACLAC + THMPP
R114	BUTAN16	ACLAC → ACETOIN + CO_2
R115	BUTAN17	AC + ACTACCOA → ACCOA + ACTAC
R116	BUTAN18	BU + ACTACCOA → BUCOA + ACTAC
R117	TCA1	PYR + ATP + HCO_3 → ADP + Pi + OAA
R118	TCA2	OAA + NADH ↔ MAL + NAD
R119	TCA3	MAL ↔ FUM
R120	TCA4	FUM + Fd(Red) ↔ SUCC + Fd(Ox)
R121	TCA5	SUCC + ACTACCOA → ACTAC + SUCCOA
R122	TCA6	CIT ↔ ICIT
R123	TCA7	ICIT + NAD ↔ AKG + CO_2 + NADH
R124	TCA8	Fd(Ox) + AKG + COA ↔ Fd(Red) + SUCCOA + CO_2
R125	PPP1	F6P + GA3P ↔ E4P + DXU5P
R126	PPP2	DXU5P ↔ DRU5P
R127	PPP3	R5P ↔ DRU5P
R128	PPP4	R5P + DXU5P ↔ S7P + GA3P
R129	PPP5	S7P + GA3P ↔ E4P + F6P
R130	PPP6	R5P + ATP → PRPP + AMP
R131	PPP7	2DDGLCN + ATP → 2DDG6P + ADP
R132	PPP8	2DDG6P ↔ GA3P + PYR
R133	PPP9	DRIB + ATP → R5P + ADP
R134	PPP10	R5P ↔ R1P
R135	PI1	DXYL + NADPH ↔ XOL + NADP
R136	PI1-2	XOL + NAD ↔ DXYLU + NADH
R137	PI2	DXYLU + ATP ↔ DXU5P + ADP
R138	PI3	LARAB ↔ LRBL
R139	PI4	LRBL + ATP ↔ LRU5P + ADP

R140	PI5	LRU5P ↔ DXU5P
R141	FM1	F1P + ATP ↔ FDP + ADP
R142	FM2	F1P → DHAP + GLYALD
R143	FM3	MNL1P + NAD ↔ F6P + NADH
R144	FM4	MAN6P ↔ F6P
R145	FM5	FRU + ATP → F6P + ADP
R146	GAL1	LCTS6P ↔ GLC + GAL6P
R147	GAL2	GAL6P ↔ TAG6P
R148	GAL3	TAG6P + ATP ↔ TAGDP + ADP
R149	GAL4	TAGDP ↔ DHAP + GA3P
R150	GAL5	GAL + ATP → GAL1P + ADP
R151	GAL6	UDPGLC + GAL1P ↔ G1P + UDPGAL
R152	GAL7	UDPGAL ↔ UDPGLC
R153	GAL8	G1P + UTP → UDPGLC + PPi
R154	GAL9	G1P ↔ G6P
R155	SUCR1	SUC6P → FRU + G6P
R156	SUCR2	MALT6P → GLC + G6P
R157	SUCR3	MALT → 2 GLC
R158	SUCR4	MALT + Pi → bDGLC + bDG1P
R159	SUCR5	bDG1P ↔ bDG6P
R160	SUCR6	G1P + ATP → ADPGLC + PPi
R161	SUCR7	ADPGLC → ADP + Glycogen
R162	SUCR8	Glycogen + Pi → G1P
R163	SUCR9	CLB(Ext) → 2 bDGLC(Ext)
R164	AMSU1	F6P + LGLN → GAM6P + LGLU
R165	AMSU2	F6P + NH3 ↔ GAM6P
R166	AMSU3	GAM6P + AC ↔ ACGAM6P
R167	AMSU4	GAM6P → GAM1P
R168	AMSU5	GAM1P + ACCOA → ACGAM1P + COA
R169	AMSU6	ACGAM1P + UTP ↔ UACGAM + PPi
R170	AMSU7	UACGAM + PEP ↔ UACCG + Pi
R171	AMSU8	UACCG + NADPH → UAMR + NADP
R172	NUSU1	dTTP + G1P → TDPGLC + PPi
R173	NUSU2	TDPGLC ↔ TDPGAL
R174	NUSU3	TDPGLC → TDPDHdGLC
R175	NUSU4	TDPDHdGLC → GDPoRHAM
R176	NUSU5	TDPDHdGLC → TDPoRHAM
R177	NUSU6	GDPoRHAM + NADPH → GDPRHAM + NADP
R178	NUSU7	TDPoRHAM + NADPH → TDPRHAM + NADP
R179	PYR1	PYR + ATP → PEP + AMP + Pi
R180	PYR2	MAL + NAD → PYR + CO2 + NADH
R181	PYR3	MAL + NADP → PYR + CO2 + NADPH
R182	PYR4	ACCOA + ATP + HCO3 ↔ MALCOA + ADP + Pi
R183	PYR5	ACCOA + 3MOB → 2IPPMAL + COA
R184	PYR6	PYR + COA → ACCOA + FORM
R185	METHANE1	CO2 + MECORR → ACCOA + CORR
R186	NITROGEN1	NO2 + 6 Fd(Red) → NH3 + 6 Fd(Ox)

R187	NITROGEN2	N2 + 16 ATP + 8 Fd(Red) → 16 Pi + 16 ADP + 8 Fd(Ox) + 2 NH3 + H2
R188	NITROGEN3	LGLU + ATP + NH3 → LGLN + ADP + Pi
R189	NITROGEN4	LGLN + AKG + NADPH → 2 LGLU + NADP
R190	NITROGEN5	LASP + LGLN + ATP → LASN + LGLU + AMP + PPi
R191	NITROGEN6	AKG + NH3 + NADPH ↔ LGLU + NADP
R192	NITROGEN7	LASP → FUM + NH3
R193	NITROGEN8	LASN → LASP + NH3
R194	NITROGEN9	CYST → LHCYS + NH3 + PYR
R195	SULFUR1	SO4 + ATP → APS + PPi
R196	SULFUR2	APS + ATP → PAPS + ADP
R197	SULFUR3	PAPS + TRD(Red) → PAP + TRD(Ox) + SO3
R198	SULFUR4	PAP → AMP + Pi
R199	SULFUR5	SO3 + 3 NADPH → S + 3 NADP
R200	SULFUR6	LSER + ACCOA → ACSER + COA
R201	SULFUR7	S + ACSER → LCYS + AC
R202	SULFUR8	LHMS + SUCCOA → SUCHMS + COA
R203	SULFUR9	SUCHMS + LCYS → CYST + SUCC
R204	GLU1	AKG + LASP ↔ OAA + LGLU
R205	GLU2	LGLU ↔ DGLU
R206	GLU3	LGLN + ATP + DNAD → LGLU + AMP + PPi + NAD
R207	GLU4	4ABUT + AKG ↔ SUCCSA + LGLU
R208	ASPALA1	LASP + ATP + LCITR → AMP + PPi + ARGSUC
R209	ASPALA2	ARGSUC → FUM + LARG
R210	ASPALA3	LASP → bALA + CO2
R211	ASPALA4	PYR + LGLU ↔ LALA + AKG
R212	ASPALA5	LALA ↔ DALA
R213	ASPALA6	LASP + O2 → OAA + NH3 + H2O2
R214	ASPALA7	2 DALA + ATP → DALADALA + ADP + Pi
R215	ASPALA8	PYR + DGLU ↔ AKG + DALA
R216	GST1	LASP + ATP → 4PASP + ADP
R217	GST2	4PASP + NADPH → ASPSA + Pi + NADP
R218	GST3	ASPSA + NADPH ↔ LHMS + NADP
R219	GST4	LHMS + ATP → PHOM + ADP
R220	GST5	PHOM → LTHR + Pi
R221	GST6	LTHR ↔ GLY + ACAL
R222	GST7	GLY + MLTHF ↔ THF + LSER
R223	GST8	LSER → PYR + NH3
R224	GST9	3PG + NAD → 3PHP + NADH
R225	GST10	3PHP + LGLU → LPSER + AKG
R226	GST11	LPSER → LSER + Pi
R227	GST12	GLYCAC + NAD ↔ HPYR + NADH
R228	MET1	SUCHMS ↔ 2OBUT + SUCC + NH3
R229	MET2	CYST + AC ↔ ACHMS + LCYS
R230	MET3	ACHMS + S → LHCYS + AC
R231	MET4	SUCHMS + S → LHCYS + SUCC
R232	MET5	LHMS + AMET → LMET + AHCYS
R233	MET6	LHCYS + 5MTHF → LMET + THF
R234	MET7	AHCYS → RHCYS + ADE

R235	MET8	RHCYS → LHCYS + DRIB
R236	MET9	ATP + LMET → AMET + Pi + PPi
R237	MET10	AMET → AMETA + CO2
R238	MET11	AMETA + PTRC → METADN + SPERMD
R239	MET12	METADN → ADE + 5METRIB
R240	CYS1	S + PYR + NH3 → LCYS
R241	VLI0	LTHR → 2OBUT + NH3
R242	VLI1	2OBUT + HETHMPP → 2AHBUT + THMPP
R243	VLI2	2AHBUT ↔ 3H3MOP
R244	VLI3	3H3MOP + NADPH ↔ 23DHMP + NADP
R245	VLI4	23DHMP → 3MOP
R246	VLI5	3MOP + LGLU ↔ LILE + AKG
R247	VLI6	ACLAC ↔ 3H3MOB
R248	VLI7	3H3MOB + NADPH ↔ 23DHMB + NADP
R249	VLI8	23DHMB → 3MOB
R250	VLI9	3MOB + LGLU ↔ LVAL + AKG
R251	VLI10	2IPPMAL ↔ 2IPPM
R252	VLI11	2IPPM ↔ 3IPPMAL
R253	VLI12	3IPPMAL + NAD ↔ 2IPSUCC + NADH
R254	VLI13	2IPSUCC → 4MOP + CO2
R255	VLI14	4MOP + LGLU ↔ LLEU + AKG
R256	LYS1	ASPSA + PYR → 23DHDP
R257	LYS2	23DHDP + NADPH ↔ THDP + NADP
R258	LYS3	THDP + SUCCOA → SL2A6O + COA
R259	LYS4	SL2A6O + LGLU ↔ SL26DA + AKG
R260	LYS5	SL26DA → SUCC + 26DAP-LL
R261	LYS6	26DAP-LL ↔ 26DAP-M
R262	LYS7	26DAP-M → LLYS + CO2
R263	LYS8	LLYS → CDV + CO2
R264	PRO1	LGLU + ATP → GLU5P + ADP
R265	PRO2	GLU5P + NADPH ↔ GLU5SA + NADP + Pi
R266	PRO3	GLU5SA ↔ 1PYR5C
R267	PRO4	1PYR5C + NADPH ↔ LPRO + NADP
R268	PRO5	LORN + AKG ↔ GLU5SA + LGLU
R269	ARG1	LARG → LORN + UREA
R270	ARG2	CBP + LORN ↔ LCITR + Pi
R271	HIS1	PRPP + ATP → PRBATP + PPi
R272	HIS2	PRBATP → PRBAMP + PPi
R273	HIS3	PRBAMP → PRFP
R274	HIS4	PRFP → PRLP
R275	HIS5	PRLP + LGLN → AICAR + LGLU + EIG3P
R276	HIS6	EIG3P → IMACP
R277	HIS7	IMACP + LGLU ↔ HISP + AKG
R278	HIS8	HISP → HISTD + Pi
R279	HIS9	HISTD + NAD → HISTDAL + NADH
R280	HIS10	HISTDAL + NAD → LHIS + NADH
R281	HIS11	LHIS + AMET → MLHIS + AHCYS
R282	PTT1	PEP + E4P → 2DDA7P + Pi

R283	PTT2	2DDA7P → 3DHQ + Pi
R284	PTT3	3DHQ ↔ 3DHSK
R285	PTT4	3DHSK + NADPH ↔ SKM + NADP
R286	PTT5	SKM + ATP → SKM3P + ADP
R287	PTT6	SKM3P + PEP ↔ 3PSME + Pi
R288	PTT7	3PSME → CHOR + Pi
R289	PTT8	CHOR + LGLN → ANTH + PYR + LGLU
R290	PTT9	ANTH + PRPP → PRAN + PPi
R291	PTT10	PRAN ↔ 2CPR5P
R292	PTT11	2CPR5P → 3IG3P + CO2
R293	PTT12	3IG3P → INDOLE + GA3P
R294	PTT13	LSER + INDOLE → LTRP
R295	PTT14	CHOR ↔ PPHN
R296	PTT15	PPHN → PHPYR + CO2
R297	PTT16	PHPYR + LGLU ↔ LPHE + AKG
R298	PTT17	PHPYR + DGLU ↔ DPHE + AKG
R299	PTT18	PPHN + NAD → 34HPP + CO2 + NADH
R300	PTT19	34HPP + LGLU ↔ LTYR + AKG
R301	TYR1	MTYRAM + AMET → HOR + AHCYS
R302	TYR2	LTYR + AKG ↔ 34HPP + LGLU
R303	TYR3	4H2KPM → SUCCSA + PYR
R304	UREA1	LGLU + ACCOA → ACGLU + COA
R305	UREA2	ACGLU + ATP → ACGLU5P + ADP
R306	UREA3	ACGLU5P + NADPH → ACGLU5SA + Pi + NADP
R307	UREA4	ACGLU5SA + LGLU ↔ ACORN + AKG
R308	UREA5	ACORN + LGLU ↔ LORN + ACGLU
R309	GTH1	H2O2 + 2 GTH(Red) → GTH(Ox)
R310	PUR1	PRPP + LGLN → PRAM + PPi + LGLU
R311	PUR2	PRAM + GLY + ATP → GAR + ADP + Pi
R312	PUR3	GAR + 10FTHF → FGAR + THF
R313	PUR4	FGAR + LGLN + ATP → FGAM + LGLU + ADP + Pi
R314	PUR5	FGAM + ATP → AIR + ADP + Pi
R315	PUR6	AIR + HCO3 ↔ PRAIC
R316	PUR7	PRAIC + LASP + ATP → SAICAR + ADP + Pi
R317	PUR8	SAICAR ↔ FUM + AICAR
R318	PUR9	AICAR + 10FTHF → FPRICA + THF
R319	PUR10	FPRICA ↔ IMP
R320	PUR11	IMP + LASP + GTP → DCAMP + GDP + Pi
R321	PUR12	DCAMP → AMP + FUM
R322	PUR13	AMP + ATP ↔ 2 ADP
R323	PUR14	ATP + TRD(Red) → dATP + TRD(Ox)
R324	PUR15	dATP + PYR → dADP + PEP
R325	PUR16	ADP + TRD(Red) → dADP + TRD(Ox)
R326	PUR17	dADP + ADP ↔ dAMP + ATP
R327	PUR18	AMP + PPi ↔ ADE + PRPP
R328	PUR19	ADE + 2DR1P ↔ dADN + Pi
R329	PUR20	dADN → dINS + NH3
R330	PUR21	dINS + Pi ↔ HXAN + 2DR1P

R331	PUR22	HXAN + R1P ↔ INS + Pi
R332	PUR23	ADN → INS + NH3
R333	PUR24	ADN + Pi ↔ ADE + R1P
R334	PUR25	ADE → HXAN + NH3
R335	PUR26	HXAN + PRPP ↔ IMP + PPi
R336	PUR27	IMP + NAD → XMP + NADH
R337	PUR28	XMP + NH3 + ATP → GMP + PPi + AMP
R338	PUR29	XMP + LGLN + ATP → GMP + PPi + LGLU + AMP
R339	PUR30	GMP + NADPH → IMP + NH3 + NADP
R340	PUR31	GMP + PPi ↔ GUA + PRPP
R341	PUR32	GUA → XAN + NH3
R342	PUR33	XAN + PRPP ↔ XMP + PPi
R343	PUR34	XANT + Pi ↔ XAN + R1P
R344	PUR35	GMP + ATP ↔ GDP + ADP
R345	PUR36	GDP + PEP ↔ GTP + PYR
R346	PUR37	GTP + TRD(Red) → dGTP + TRD(Ox)
R347	PUR38	dGTP + PYR ↔ dGDP + PEP
R348	PUR39	GDP + TRD(Red) → dGDP + TRD(Ox)
R349	PYRM1	LGLN + 2 ATP + HCO3 → LGLU + CBP + 2 ADP + Pi
R350	PYRM2	CBP + LASP → CBASP + Pi
R351	PYRM3	CBASP ↔ DHOR-S
R352	PYRM4	DHOR-S + NAD ↔ OROT + NADH
R353	PYRM5	OROT + PRPP → OROT5P + PPi
R354	PYRM6	OROT5P → UMP + CO2
R355	PYRM7	UMP + ATP ↔ UDP + ADP
R356	PYRM8	UDP + ATP ↔ UTP + ADP
R357	PYRM9	UTP + NH3 + ATP → CTP + ADP + Pi
R358	PYRM10	UTP + LGLN + ATP → CTP + LGLU + ADP + Pi
R359	PYRM11	CTP → UTP + NH3
R360	PYRM12	CTP + ADP ↔ CDP + ATP
R361	PYRM13	CDP + ADP ↔ CMP + ATP
R362	PYRM14	CTP + TRD(Red) → dCTP + TRD(Ox)
R363	PYRM15	CDP + TRD(Red) → dCDP + TRD(Ox)
R364	PYRM16	TRD(Ox) + NADPH → TRD(Red) + NADP
R365	PYRM17	dCTP + ADP ↔ dCDP + ATP
R366	PYRM18	dCDP + ADP ↔ dCMP + ATP
R367	PYRM19	dCMP → dUMP + NH3
R368	PYRM20	UTP + TRD(Red) → dUTP + TRD(Ox)
R369	PYRM21	dCTP → dUTP + NH3
R370	PYRM22	dUTP → dUMP + PPi
R371	PYRM23	dUTP + ADP ↔ dUDP + ATP
R372	PYRM24	dUDP + ADP ↔ dUMP + ATP
R373	PYRM25	UDP + TRD(Red) → dUDP + TRD(Ox)
R374	PYRM26	dUMP + MLTHF → dTMP + DHF
R375	PYRM27	dTMP + ATP ↔ dTDP + ADP
R376	PYRM28	dTDP + ATP ↔ dTTP + ADP
R377	PL1	ATP + GLYCAC → ADP + 3PG
R378	PL2	ATP + GLYC → ADP + GLYC3P

R379	PL3	GLYC3P + 0.073 C140-ACP + 0.521 C160-ACP + 0.065 C161-ACP + 0.036 C180-ACP + 0.102 C181-ACP + 0.022 C17CYC-ACP + 0.181 C19CYC-ACP → 1MAG + ACP
R380	PL4	1MAG + 0.073 C140-ACP + 0.521 C160-ACP + 0.065 C161-ACP + 0.036 C180-ACP + 0.102 C181-ACP + 0.022 C17CYC-ACP + 0.181 C19CYC-ACP → PA + ACP
R381	PL5	ATP + 12DAG → ADP + PA
R382	PL6	GLYC3P + NAD ↔ DHAP + NADH
R383	PL7	GLYC3P + NADP ↔ DHAP + NADPH
R384	PL8	PA + CTP → CDP-DAG + PPi
R385	PL9	CDP-DAG + GLYC3P → CMP + PGP
R386	PL10	PGP → PG + Pi
R387	PL11	2 PG → CDL + GLYC
R388	PL12	PG + CDP-DAG → CDL + CMP
R389	PL13	CDP-DAG + LSER → CMP + PS
R390	PL14	PS → PE + CO2
R391	PL15	12 PG → 12 12DAG + POLYGP
R392	FAS1	ACCOA + ACP ↔ ACACP + COA
R393	FAS2	MALCOA + ACP ↔ MALACP + COA
R394	FAS3	ACACP + 6 MALACP + 12 NADPH → 12 NADP + C140-ACP + 6 CO2 + 6 ACP
R395	FAS4	ACACP + 7 MALACP + 14 NADPH → 14 NADP + C160-ACP + 7 CO2 + 7 ACP
R396	FAS5	ACACP + 7 MALACP + 13 NADPH → 13 NADP + C161-ACP + 7 CO2 + 7 ACP
R397	FAS6	ACACP + 8 MALACP + 16 NADPH → 16 NADP + C180-ACP + 8 CO2 + 8 ACP
R398	FAS7	ACACP + 8 MALACP + 15 NADPH → 15 NADP + C181-ACP + 8 CO2 + 8 ACP
R399	FAS8	C161-ACP + AMET → C17CYC-ACP + AHCYS
R400	FAS9	C181-ACP + AMET → C19CYC-ACP + AHCYS
R401	STERO1	DMPP + IPDP → GRDP + PPi
R402	STERO2	GRDP + IPDP → FRDP + PPi
R403	STERO3	FRDP + IPDP → GGRDP + PPi
R404	STERO4	GGRDP + 7 IPDP → UDCPDP + 7 PPi
R405	STERO5	PYR + GA3P → dXYLU5P + CO2
R406	STERO6	dXYLU5P + NADPH → MERYTH4P + NADP
R407	STERO7	MERYTH4P + CTP → CDPMERYTH + PPi
R408	STERO8	CDPMERYTH + ATP → CDPMERY2P + ADP
R409	STERO9	CDPMERY2P → MERYcDP + CMP
R410	STERO10	MERYcDP + ProDTH → HMB4DP + ProDS
R411	NAD1	LASP + FORM + ACCOA → QULN
R412	NAD2	QULN + PRPP → NAMN + PPi + CO2
R413	NAD3	ATP + NAMN → PPi + DNAD
R414	NAD4	ATP + NAD ↔ ADP + NADP
R415	NAD5	NA + PRPP → NAMN + PPi
R416	NAD6	ATP + NMN → PPi + NAD
R417	NAD7	NAMNs + Pi ↔ NA + R1P
R418	PANCOA1	3MOB + MLTHF → THF + 2DHP

R419	PANCOA2	2DHP + NADPH → PANT + NADP
R420	PANCOA3	ATP + PANT + bALA → AMP + PPi + PNTO
R421	PANCOA4	ATP + PNTO → ADP + 4PPAN
R422	PANCOA5	ATP + 4PPAN + LCYS → ADP + Pi + 4PPCYS
R423	PANCOA6	CTP + 4PPAN + LCYS → CDP + Pi + 4PPCYS
R424	PANCOA7	4PPCYS → PAN4P + CO2
R425	PANCOA8	ATP + PAN4P → PPi + DPCOA
R426	PANCOA9	ATP + DPCOA → ADP + COA
R427	PANCOA10	ATP + 4PCYS → ADP + 4PPCYS
R428	PANCOA11	ATP + PAN → ADP + PAN4P
R429	RIBFLA1	DRU5P → DB4P + FORM
R430	RIBFLA2	4R5AU + DB4P → DMLZ + Pi
R431	RIBFLA3	GTP → FORM + 25DRAPP + PPi
R432	RIBFLA4	25DRAPP → 5APRBU + NH3
R433	RIBFLA6	5APRBU + NADP → 5APRU + NADPH
R434	RIBFLA7	5APRU → 4R5AU + Pi
R435	RIBFLA8	2 DMLZ → RIBFLA + 4R5AU
R436	RIBFLA9	ATP + RIBFLA → ADP + FMN
R437	RIBFLA10	ATP + FMN → PPi + FAD
R438	RIBFLA11	RIBFLA → DMBZID
R439	RIBFLA12	NAMN + DMBZID → NA + 5PRDMBZ
R440	FOLATE1	GTP → FAPTP
R441	FOLATE2	FAPTP → DAPTP + FORM
R442	FOLATE3	DAPTP → DATHAO
R443	FOLATE4	DATHAO → AHTHDH
R444	FOLATE5	AHTHDH → DHNPP + PPi
R445	FOLATE6	DHNPP → DHNP + Pi
R446	FOLATE7	DHNP → GLYCALD + AHHMDHP
R447	FOLATE8	ATP + AHHMDHP → AMP + ADHHP
R448	FOLATE9	ADHHP + PABA → PPi + DHPT
R449	FOLATE10	AHHMDHP + PABA → DHPT
R450	FOLATE11	ATP + DHPT + LGLU → ADP + Pi + DHF
R451	FOLATE12	DHF + NADP ↔ FOL + NADPH
R452	FOLATE13	THF + NADP ↔ DHF + NADPH
R453	FOLATE14	ATP + 5FTHF → ADP + Pi + METHF
R454	FOLATE15	METHF → 5FTHF
R455	FOLATE16	MLTHF + NADH → 5MTHF + NAD
R456	FOLATE17	10FTHF ↔ METHF
R457	FOLATE18	MLTHF + NADP ↔ METHF + NADPH
R458	FOLATE19	THF + FORM + ATP → ADP + Pi + 10FTHF
R459	PORCHL1	LGLU + NADPH + ATP → GLU1SA + AMP + NADP + PPi
R460	PORCHL2	GLU1SA → 5AOP
R461	PORCHL3	2 5AOP → PPBNG
R462	PORCHL4	4 PPBNG → HMBIL + 4 NH3
R463	PORCHL5	HMBIL → UPPG3
R464	PORCHL6	CPPPG3 + 2 AMET → PPPG9 + 2 CO2 + 2 LMET + 2 dADN
R465	PORCHL7	2 AMET + UPPG3 → 2 AHCYS + PRCR2
R466	PORCHL8	PRCR2 + NAD → SHCL + NADH
R467	PORCHL9	Fe2 + SHCL → SHEME

R468	PORCHL10	SHCL + COBALT → CPRCR2
R469	PORCHL11	CPRCR2 + AMET → CPRCR3 + AHCYS
R470	PORCHL12	CPRCR3 + AMET → CPRCR4 + AHCYS
R471	PORCHL13	CPRCR4 + AMET → CPRCR5A + AHCYS
R472	PORCHL14	CPRCR5A → CPRCR5B + ACAL
R473	PORCHL15	CPRCR5B + AMET → CPRCR6 + AHCYS
R474	PORCHL16	CPRCR6 + NADPH → CDHPRCR6 + NADP
R475	PORCHL17	CDHPRCR6 + AMET → CPRCR7 + AHCYS
R476	PORCHL18	CPRCR7 + AMET → CPRCR8 + AHCYS + CO2
R477	PORCHL19	CPRCR8 → CBRN
R478	PORCHL20	CBRN + 2 LGLN + 2 ATP → CBRNDA + 2 LGLU + 2 ADP + 2 Pi
R479	PORCHL21	AMET + PRCR3B → AHCYS + PRCR4
R480	PORCHL22	AMET + PRCR4 → AHCYS + PRCR5
R481	PORCHL23	PRCR6A + NADPH → PRCR6B + NADP
R482	PORCHL24	PRCR8 → HGBRN
R483	PORCHL25	ACBRNDA + 4 LGLN + 4 ATP → ACBRNHA + 4 LGLU + 4 Pi + 4 ADP
R484	PORCHL26	ATP + ACBRNHA + 1APROH → ADP + Pi + ACBA
R485	PORCHL27	ACBA + ATP → ACBAP + ADP
R486	PORCHL28	ACBA + GTP → ACBAP + GDP
R487	PORCHL29	ACBRNHA + APROHP + ATP → ACBAP + ADP + Pi
R488	PORCHL30	ACBAP + GTP → AGDPCBA + PPi
R489	PORCHL31	AGDPCBA + ARBZL → CACO + GMP
R490	PORCHL32	ARBZL5P → ARBZL + Pi
R491	LIMPIN1	HIPCOA + NAD → IPCHCCOA + NADH
R492	LIMPIN2	MTNOL + O2 + NAD → MTNAL + NADH
R493	LIMPIN3	HDMHCOA + NAD → DMMOHCOA + NADH
R494	DNA	1.118 dATP + 0.501 dCTP + 1.118 dTTP + 0.501 dGTP + 4.403 ATP → 4.403 ADP + 4.403 Pi + 3.236 PPi + DNA
R495	RNA	1.05 ATP + 1.124 CTP + 0.873 UTP + 0.832 GTP → 1.554 ADP + 1.554 Pi + 3.879 PPi + RNA
R496	PROTEIN	0.775 LALA + 0.133 LARG + 0.156 LASN + 0.156 LASP + 1.216 LCYS + 0.127 LGLN + 0.127 LGLU + 1.078 GLY + 0.146 LHIS + 0.436 LILE + 0.429 LLEU + 0.336 LLYS + 0.783 LMET + 0.185 LPHE + 0.457 LPRO + 0.427 LSER + 0.41 LTHR + 0.043 LTRP + 0.801 LTYR + 1.172 LVAL + 37.195 ATP → 37.195 ADP + 37.195 Pi + PROTEIN
R497	PLIPID	0.8 PE + 0.397 PG + 0.109 CDL → PLIPID
R498	TEICHOIC	0.518 POLYGP + 0.129 LLYS + 0.129 UACGAM + 0.129 ATP → TEICH + 0.129 UDP + 0.129 ADP + 0.129 Pi
R499	TRACE	0.215 NAD + 0.192 NADP + 0.199 COA + 0.321 THF + 0.313 FMN + 0.182 FAD → TRACE
R500	PEPTIDO	1.064 UAMR + 1.064 UACGAM + 1.106 LALA + 1.106 LGLU + 1.106 DALADALA + 1.106 26DAP-M + 4.425 ATP → PEPTIDO + 1.106 DALA + 1.106 UDP + 1.106 UMP + 4.425 ADP + 4.425 Pi
R501	CARBO	2.058 UDPGLC + 4.115 UDPGAL → 6.173 UDP + CARBO

R502	BIOMASS	0.5284 PROTEIN + 0.0655 RNA + 0.026 DNA + 0.076 PLIPID + 0.1009 PEPTIDO + 0.08 TEICH + 0.0432 CARBO + 0.0494 TRACE + 40 ATP → BIOMASS + 40 ADP + 40 Pi

References

Number	Authors	Year	issue;volume;page	Publisher	Remark
1	Ali, M.K., Rudolph, F.B., and Bennett, G.N.	2004	31(5):229-234	J. Ind. Microbiol. Biotechnol.	
2	Andersch, W., Bahl, H., and Gottschalk, G.	1983	18:327-332	Eur. J. Appl. Microbiol. Biotechnol.	DSM 1732 Strain
3	Annous, B.A., and Blaschek, H.P.	1994	13:10-16	J. Ind. Microbiol.	
4	Behrens, S., Mitchell, W., and Bahl, H.	2001	147:75-86	Microbiology.	
5	Belouski, E., Watson, D.E., and Bennett, G.N.	1998	37(1):17-22	Curr. Microbiol.	
6	Boynton, Z.L., Bennett, G.N., and Rudolph, F.B.	1996	62(8):2758-2766	Appl Environ Microbiol.	
7	Boynton, Z.L., Bennett, G.N., and Rudolph, F.B.	1996	178(11):3015-3024	J. Bacteriol.	
8	Cary, J.W., Petersen, D.J., Papoutsakis, E.T., and Bennett, G.N.	1990	56(6):1576-1583	Appl. Environ. Microbiol.	
9	Cary, J.W., Petersen, D.J., Papoutsakis, E.T., and Bennett, G.N.	1988	170(10):4613-4618	J. Bacteriol.	
10	Chen, J.S., Toth, J., and Kasap, M.	2001	27(5):281-286	J. Ind. Microbiol. Biotechnol.	
11	Contag, P.R., Williams, M.G., and Rogers, P.	1990	56(12):3760-3765	Appl. Environ. Microbiol.	B643 Strain
12	Damiano, V.B., Ward, R., Gomes, E., Alves-Prado, H.F., and Da Silva, R.	2006	129-132:289-302	Appl. Biochem. Biotechnol.	
13	Durre, P. (Ed.)	2005	920 pages	CRC Press, Taylor & Francis Group	
14	Edberg, S.C	1991	Unpublished	U.S. Enviromental Protection Agency	http://www.epa.gov/oppt/biotech/pubs/fra/fra003.htm
15	Fischer, R.J., Oehmcke, S., Meyer, U., Mix, M., Schwarz, K., Fiedler, T., and Bahl, H.	2006	188(15):5469-5478	J. Bacteriol.	
16	Fontaine, L., Meynial-Salles, I., Girbal, L., Yang, X., Croux, C., and Soucaille, P.	2002	184(3):821-830	J. Bacteriol.	
17	Gerischer, U., and Durre, P.	1990	172(12):6907-6918	J. Bacteriol.	DSM 792 Strain
18	Gorwa, M.F., Croux, C., and Soucaille, P.	1996	178(9):2668-2675	J. Bacteriol.	
19	Huang, K., Rudolph, F.B., and Bennett, G.N.	1999	65(7):3244-3247	Appl. Environ. Microbiol.	
20	Iddar, A., Valverde, F., Serrano, A., and Soukri, A.	2002	25(3):519-526	Protein. Expr. Purif.	
21	Jones, D.T., and Woods, D.R.	1986	50(4):484-524	Microbiol. Rev.	
22	Lee, S.F., and Forsberg, C.W.	1987	53(4):651-654	Appl. Environ. Microbiol.	
23	Lee, S.F., Forsberg, C.W., and Gibbins, L.N.	1985	50(2):220-228	Appl. Environ. Microbiol.	
24	López-Contreras, A.M., Gabor, K., Martens, A.A., Renckens, B.A., Claassen, P.A., Van Der Oost, J., and De Vos, W.M.	2004	70(9):5238-5243	Appl. Environ. Microbiol.	
25	Meinecke, B., Bertram, J., and Gottschalk, G.	1989	152(3):244-250	Arch. Microbiol.	
26	Mes-Hartree, M., and Saddler, J.N.	1982	4(4):247-252	Biotechnol. Lett.	
27	Nair, R.V., Bennett, G.N., and Papoutsakis, E.T.	1994	176(3):871-885	J. Bacteriol.	
28	Ounine, K., Petitdemange, H., Raval, G., and Gay, R.	1983	5(9):605-610	Biotechnol. Lett.	
29	Paquet, V., Croux, C., Goma, G., and Soucaille, P.	1991	57(1):212–218	Appl. Environ. Microbiol.	
30	Petersen, D.J., and Bennett, G.N.	1991	57(9):2735-2741	Appl. Environ. Microbiol.	
31	Sabathe, F., Belaich, A., and Soucaille, P.	2002	217(1):15-22	FEMS Microbiol Lett.	
32	Schreiber, W., and Durre, P.	1999	145(8):1839-1847	Microbiology.	DSM 792 Strain
33	Tangney, M., and Mitchell, W.J.	2007	74(2):398-405	Appl. Microbiol. Biotechnol.	
34	Tangney, M., and Mitchell, W.J.	2000	2(1):71-80	J. Mol. Microbiol. Biotechnol.	
35	Tangney, M., Winters, G.T., and Mitchell, W.J.	2001	27(5):298-306	J. Ind. Microbiol. Biotechnol.	
36	Usdin, K.P., Zappe, H., Jones, D.T., and Woods, D.R.	1986	52(3):413-419	Appl. Environ. Microbiol.	P262 Strain
37	Walter, K.A., Bennett, G.N., and Papoutsakis, E.T.	1992	174(22):7149-7158	J. Bacteriol.	
38	Waterson, R.M., Castellino, F.J., Hass, G.M., and Hill, R.L.	1972	247(16):5266-5271	J. Biol. Chem.	
39	Wiesenborn, D.P., Rudolph, F.B., and Papoutsakis, E.T.	1989	5(2):323-329	Appl. Environ. Microbiol.	
40	Winzer, K., Lorenz, K., and Durre, P.	1997	143(10):3279-3286	Microbiology.	DSM 1731 Strain
41	Yu, Y., Tangney, M., Aass, H.C., and Mitchell, W.J.	2007	73(6):1842-50	Appl. Environ. Microbiol.	
