# Supplementary material for: Oxidoreduction potential controlling for increasing the fermentability of enzymatically hydrolyzed steam-exploded corn stover for butanol production
Source: Microb Cell Fact. 2022 Jun 27;21:130. doi: 10.1186/s12934-022-01824-2 (PMC9238237; doi:10.1186/s12934-022-01824-2)
Supplement: Supplementary file 2 — Additional file 2. Low molecular weight compounds released from lignin due to degradation. [file 12934_2022_1824_MOESM2_ESM.docx]

**Additional file S2**

Gas chromatography–mass spectrometry (GC–MS) is considered because it has been proven to be a very suitable technique to analyze low molecular weight compounds released from lignin due to degradation

*Measurement method*

Sample preparation was followed the method of [Fenske et al. (1998)](#_ENREF_1). Bis-(trimethylsilyl)trifluoroacetamide (BSTFA) was used for silyl derivatization. The inhibitors in the hydrolysate were analyzed by a gas chromatography/mass spectrometry (GC–MS) (6890 N-5973, Agilent Cooperation, USA). The GC is equipped with column of 30 m × 0.25 mm DB and N_2_ at a flow rate of 1 ml/min as carrier gas. Mass spectra were obtained at 70 eV.

*Measurement result*





Fig. A.1. The GC-MS spectrum of enzymatically hydrolyzed steam-exploded corn stover

Table B.1 Compounds identified in enzymatically in hydrolyzed steam-exploded corn stover

| No. | Compound |
| --- | --- |
| 1 | 3 -metlioxy-4-hydroxy-benzaldehyde ( vanillin) |
| 2 | 4-hydroxy-benzaldehyde |
| 3 | 2-phenyl-1,2-bishydroxy-propane |
| 4 | 3-methoxy-4-hydroxy-phenyl acetic acid |
| 5 | 4-hydroxy-phenyl acetic acid |
| 6 | 4-(4-metlioxyphenyl) -2-butanone |
| 7 | 4-methoxy-phenyl acetic acid |
| 8 | 3-methoxy-4-hydroxy benzoic acid(vanillic acid) |
| 9 | 4-hydroxybenzoic acid |
| 10 | 3'5-dimetlioxy-4-hydroxy benozic acid ( syringic acid) |
| 11 | 3，5-dimethoxy-4-hydroxy-benzaldehyde( syringaldehyde) |
| 12 | 2-hydroxy-phenyl ethanol |
| 13 | Catechol |
| 14 | 2-Methoxy-4-vinylphenol |

*Reference*

Fenske, J., Griffin, D., Penner, M. 1998. Comparison of aromatic monomers in lignocellulosic biomass prehydrolysates. *Journal of Industrial Microbiology and Biotechnology*, **20**(6), 364-368.
