# Supplementary material for: Oxidoreduction potential controlling for increasing the fermentability of enzymatically hydrolyzed steam-exploded corn stover for butanol production
Source: Microb Cell Fact. 2022 Jun 27;21:130. doi: 10.1186/s12934-022-01824-2 (PMC9238237; doi:10.1186/s12934-022-01824-2)
Supplement: Supplementary file 3 — Additional file 3. The fermentation profiles of C. acetobutylicum ATCC 824 under different ORP levels which started at 0 h. [file 12934_2022_1824_MOESM3_ESM.docx]

**Additional file 3**

The fermentation profiles of *C. acetobutylicum* ATCC 824 under different ORP levels which started at 0 h.


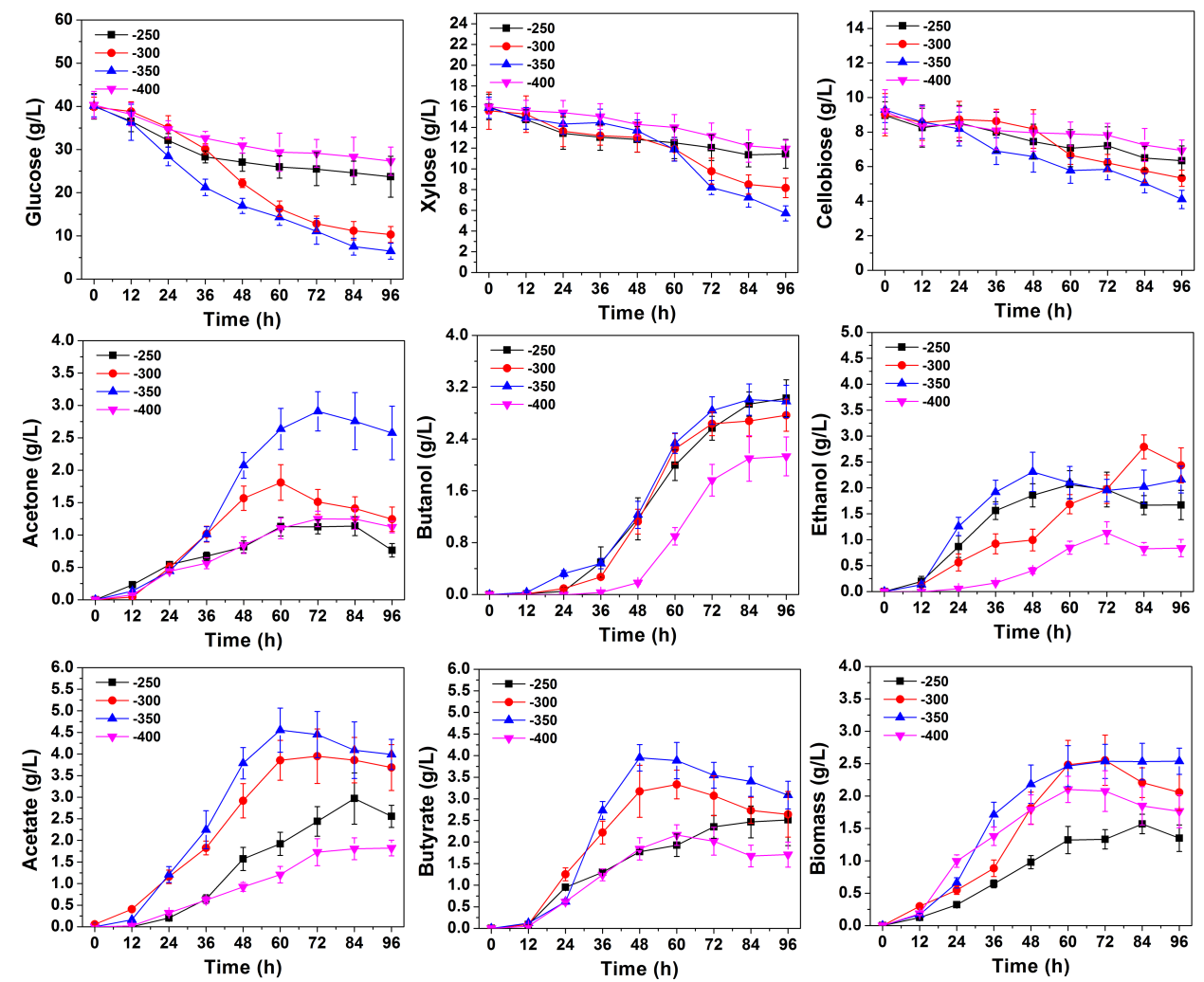


**Fig. C.1.** The fermentation profiles of *C. acetobutylicum* ATCC 824 under different ORP levels, which were started at the beginning (0 h) of the fermentation.
