# Supplementary material for: Oxidoreduction potential controlling for increasing the fermentability of enzymatically hydrolyzed steam-exploded corn stover for butanol production
Source: Microb Cell Fact. 2022 Jun 27;21:130. doi: 10.1186/s12934-022-01824-2 (PMC9238237; doi:10.1186/s12934-022-01824-2)
Supplement: Supplementary file 4 — Additional file 4. The gas production profiles of C. acetobutylicum ATCC 824 under different ORP levels. [file 12934_2022_1824_MOESM4_ESM.docx]

**Additional file 4**

The gas production profiles of *C. acetobutylicum* ATCC 824 under different ORP levels.


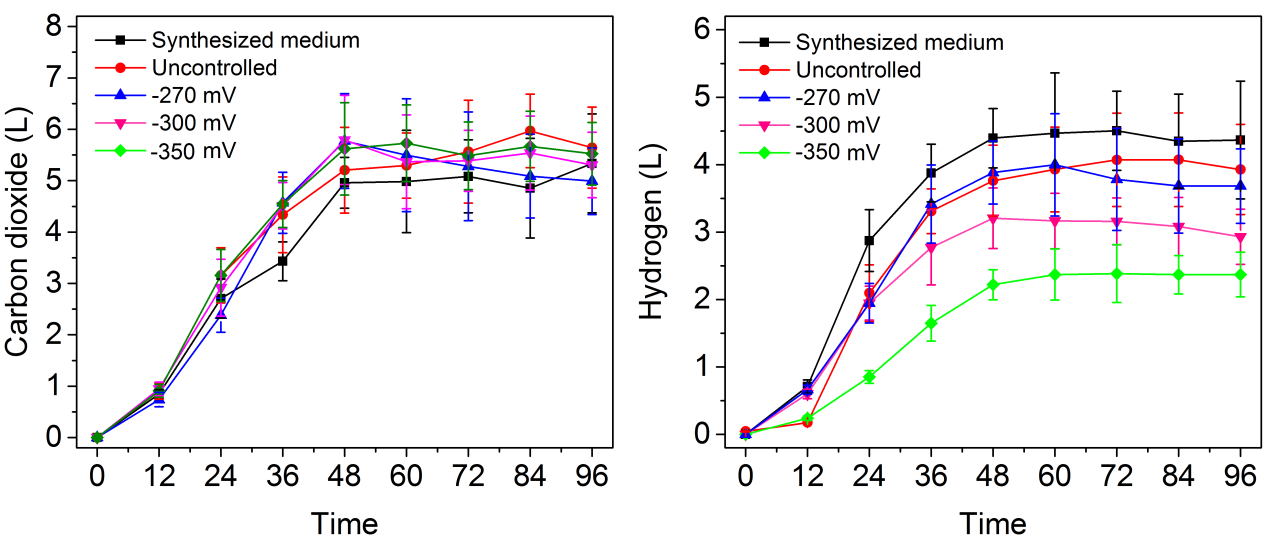


**Fig. E.1.** The gas production profiles of *C. acetobutylicum* ATCC 824 under different ORP levels. The ORP controlling were started at the beginning (24 h) at -270 mV, -300 mV and -350 mV. *Uncontrolled* represents the enzymatically hydrolyzed steam-exploded corn stover (SECS) medium without ORP control.
