# Supplementary material for: Oxidoreduction potential controlling for increasing the fermentability of enzymatically hydrolyzed steam-exploded corn stover for butanol production
Source: Microb Cell Fact. 2022 Jun 27;21:130. doi: 10.1186/s12934-022-01824-2 (PMC9238237; doi:10.1186/s12934-022-01824-2)
Supplement: Supplementary file 5 — Additional file 5. Calculation process of the specific growth rates (μ), specific substrate consumption rates, and specific product secretion rates. [file 12934_2022_1824_MOESM5_ESM.docx]

**Additional file 5**

**The calculation process**

The *Boltzmann* function was used for data fitting of biomass and metabolite molar concentrations, followed by a numerical differentiation to compute the changing rates. Then the specific growth rates (*µ*), specific substrate consumption rates, and specific product secretion rates were calculated as following.

$$\mu\text{=}\frac{d_{Biamoss}}{dt}$$

$$r_{substrate or product}=\frac{dc_{substrate \text{or} product}}{dt\bullet biomass}$$

**The fitted results of biomass of OGG and UCG**


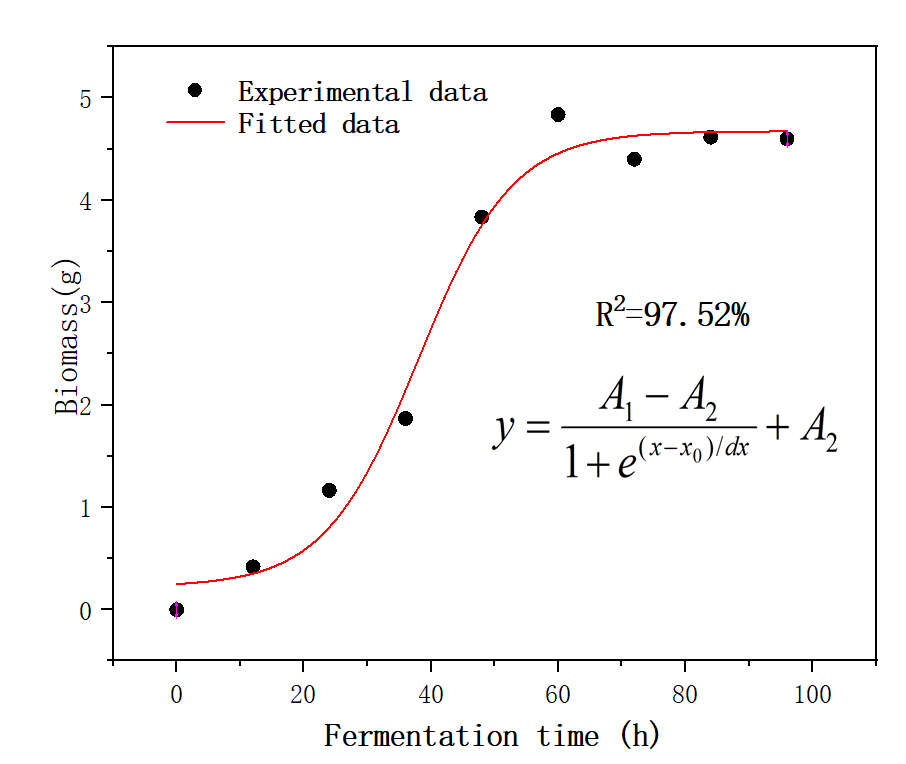


**Figure E.1 the fitted result of biomass from OCG with the *Boltzmann* model**


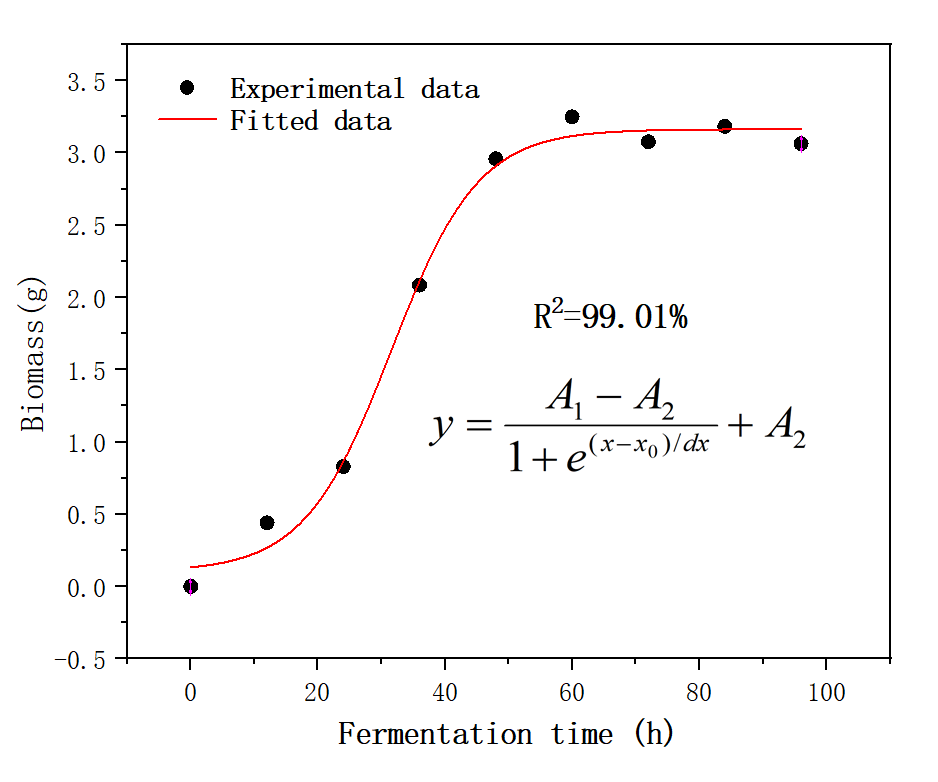


**Figure E.2 the fitted result of biomass from UCG with the *Boltzmann* model**

**The model-fitting code using MATLAB script**

*function [f_self,f_diff] = Boltzmann_fit(x,y,beta0)*

*% x represents the time points*

*% y represents the biomass data*

*% beta0: Parameters potential initial Values*

*% f_self is the function handle of fitted biomass*

*% f_diff is the function handle of the specific growth rates*

*f=@(A,x)(A(2) + (A(1)-A(2))./(1 + exp((x-A(3))./A(4))));*

*beta = nlinfit(x,y,f,beta0);*

*f_self=@(t)f(beta,t);*

*syms x;*

*f2=f(beta,x);*

*f3=diff(f2,x);*

*f_diff=matlabFunction(f3);*

*end*
