# Supplementary material for: Oxidoreduction potential controlling for increasing the fermentability of enzymatically hydrolyzed steam-exploded corn stover for butanol production
Source: Microb Cell Fact. 2022 Jun 27;21:130. doi: 10.1186/s12934-022-01824-2 (PMC9238237; doi:10.1186/s12934-022-01824-2)
Supplement: Supplementary file 9 — Additional file 9. The changes of cell wall of C. acetobutylicum ATCC 824 under different culture conditions. [file 12934_2022_1824_MOESM9_ESM.docx]

**Additional file 9**

The changes of cell wall of *C. acetobutylicum* ATCC 824 under different culture conditions captured by scanning electron microscopy (SEM).

**Methods**

For observation by SEM, biological specimens are conventionally fixed with glutaraldehyde and dehydrated in 30%, 50%, 70%, 90%, and 100% ethanol. The dried C.acetobutylicum mounted on a double sided tape on aluminum stubs and were sputter coated with gold using the fine coat ion sputter and then micrographs were recorded using the scanning electron microscope (FEI_Apreo, America).

**Results**


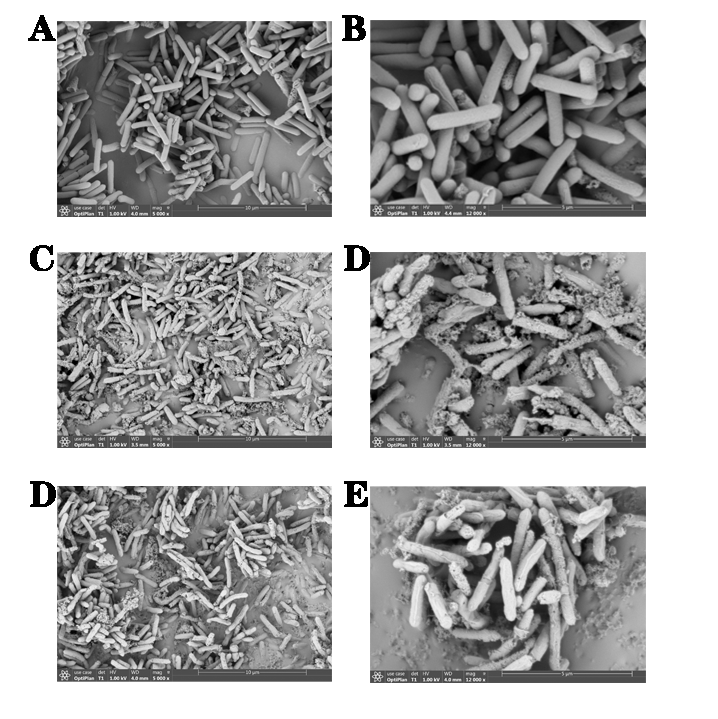


**Fig. I.1.** A and B are electron microscope photos of the *Clostridium* strains from the synthetic medium (SG) under the magnification of 5000 and 12000 times, respectively; C and D are electron microscope photos of the *Clostridium* strains from the detoxicated SECS medium without ORP control (UCG) under the magnification of 5000 and 12000 times, respectively; E and F are electron microscope photos of the *Clostridium* strains growing in the ORP of the detoxicated SECS at -350 mV (OCG) under the magnification of 5000 and 12000 times, respectively.

As the results show, the worst microstructural damage on the cell wall can be seen clearly with the cells grown in hydrolysate broth, followed by those under ORP control and synthetic media.
